# Machine learning approaches for crystallographic classification from synthetic 2D X-ray diffraction data

**DOI:** 10.1107/S1600576726000099

**Published:** 2026-02-01

**Authors:** Ayoub Shahnazari, Zeliang Zhang, Sachith E. Dissanayake, Chenliang Xu, Niaz Abdolrahim

**Affiliations:** a Department of Mechanical Engineering University of Rochester Rochester New York14627 USA; b Department of Computer Science University of Rochester Rochester New York14627 USA; c Department of Physics and Astronomy James Madison University Harrisonburg Virginia22807 USA; d Material Science Program University of Rochester Rochester New York14627 USA; e Laboratory for Laser Energetics (LLE) University of Rochester Rochester New York14627 USA; DESY, Hamburg, Germany

**Keywords:** synthetic 2D X-ray diffraction patterns, convolutional neural networks, CNNs, crystal system classification, space group classification, Auto Diffraction Pipeline, crystallographic information files, CIFs

## Abstract

Crystallographic structure identification is crucial for understanding material properties; however, current methodologies often depend on labor-intensive and time-consuming analyses of 2D X-ray diffraction (XRD) patterns. To address these limitations, this study employs synthetic 2D XRD patterns combined with deep learning (DL) techniques to enable automated and high-throughput classification of the seven crystal systems and 230 space groups. We introduce the novel Auto Diffraction Pipeline, designed to generate synthetic 2D XRD spot patterns from crystallographic information files under diverse conditions, including varying zone axes, atomic substitution, atomic depletion and mechanical loading. These conditions enhance the realism of synthetic data, mitigating the scarcity of experimental datasets and enabling the creation of large representative training sets. Convolutional neural networks were trained and validated on these synthetic datasets to classify crystallographic structures across multiple scenarios. Our results demonstrate that integrating synthetic 2D XRD patterns with DL facilitates rapid, accurate and automated crystallographic classification, promoting the wider adoption of data-driven approaches in materials science.

## Abstract 2

This study introduces the novel Auto Diffraction Pipeline to generate large-scale synthetic 2D X-ray diffraction datasets from crystallographic information files. By training convolutional neural networks on these diverse datasets, the authors achieve rapid and automated classification across all seven crystal systems and 230 space groups.

## 1. Introduction

Identifying and classifying space groups and crystal systems are essential for understanding crystal structures, as they define the symmetry and atomic arrangement (Kittel & McEuen, 2018 ▸; Chakraborty & Sharma, 2022 ▸; Thomas *et al.*, 2021 ▸). The classification of crystal symmetry begins with the seven crystal systems and can be further divided into 230 space groups through the 32 crystal classes, which align with the 32 point groups according to their specific symmetries (Ra *et al.*, 2021 ▸; Fu *et al.*, 2024 ▸; Müller & de la Flor, 2024 ▸). This inherent symmetry plays a crucial role in determining material properties, including mechanical strength, thermal and electrical conductivity, chemical reactivity, and ferroelectric phase transitions (Nye, 1985 ▸; Li *et al.*, 2022 ▸; Liu *et al.*, 2022 ▸; Rachwalski, 2023 ▸; Mohammadi & Singh, 2025 ▸).

X-ray diffraction (XRD) is a powerful, non-destructive technique widely used in materials science and crystallography for identifying and characterizing crystalline materials (Awan, 2020 ▸; Alderton & Elias, 2021 ▸; Ali *et al.*, 2022 ▸). When an X-ray beam strikes a crystal, it interacts with atomic planes and, if Bragg’s law is satisfied, constructive interference produces a diffracted beam (Cullity & Smoluchowski, 1957 ▸; Chapuis, 2021 ▸). For a 1D XRD pattern, the intensity of the diffracted beam is measured as a function of diffraction angle (2θ) using a point or line detector (Harrington & Santiso, 2021 ▸; Burns *et al.*, 2023 ▸). In contrast, a 2D XRD pattern is obtained with an area detector, which records diffracted X-rays across a 2D surface, providing a detailed map of intensity distribution. Single crystals typically exhibit spot patterns on the detector, whereas polycrystalline samples produce circular Debye–Scherrer rings (Cullity & Smoluchowski, 1957 ▸; He, 2018 ▸).

Extracting crystallographic information from diffraction data necessitates profound expertise in crystallography, along with substantial experience and analytical skills (Degen *et al.*, 2014 ▸; Guo *et al.*, 2024 ▸). Significant progress has been made in computational tools for the visualization, calibration and reduction of diffraction data, exemplified by software packages such as *VESTA* (Momma & Izumi, 2011 ▸), *pyFAI* (Ashiotis *et al.*, 2015 ▸) and *DENNIS* (Brown *et al.*, 2024 ▸), alongside established tools like *DIOPTAS* (Prescher & Prakapenka, 2015 ▸). Despite advancements in software and measurement tools, the analysis of diffraction data for materials characterization remains a complex, time-intensive and error-prone process, even for expert crystallographers, particularly when handling large or intricate datasets (Maffettone *et al.*, 2021 ▸; Yamashita *et al.*, 2021 ▸). Recent progress in computational approaches, machine learning and deep learning, particularly convolutional neural networks (CNNs), has demonstrated remarkable efficacy in overcoming the challenges associated with analyzing 1D and 2D diffraction patterns, such as crystal structure classification (Tiong *et al.*, 2020 ▸; Zaloga *et al.*, 2020 ▸; Li *et al.*, 2021 ▸), phase identification and quantification (Lee *et al.*, 2020 ▸; Lee *et al.*, 2021 ▸; Uryu *et al.*, 2024 ▸; Yue, Mehdi *et al.*, 2024 ▸; Yue, Tripathi *et al.*, 2024 ▸), crystallite size determination (Sang & Quoc Thi, 2024 ▸), strain analysis (Munshi *et al.*, 2022 ▸), texture characterization (Wanni *et al.*, 2024 ▸), and indexing (Ding *et al.*, 2020 ▸).

Several CNN models have been developed for classifying crystal systems and space groups on the basis of 1D diffraction patterns (Suzuki *et al.*, 2020 ▸; Venkatraman & Carvalho, 2024 ▸). Park *et al.* (2017 ▸) designed three CNN models to classify space groups, extinction groups and crystal systems using 150000 simulated powder 1D XRD patterns and achieved high accuracy on the simulated patterns. Vecsei *et al.* (2019 ▸) trained both a dense neural network (DNN) and a CNN, evaluating their performance on the experimentally indexed RRUFF dataset (Downs, 2015 ▸). While their CNN achieved 76% accuracy on synthetic patterns, this dropped to 42% on RRUFF data. They also found the DNN to outperform the CNN in some cases. Liu *et al.* (2019 ▸) developed a CNN model using the atomic pair distribution function to classify the 45 most frequently represented space groups, achieving an accuracy of 70%. Salgado *et al.* (2023 ▸) employed deep learning to automate XRD pattern classification in simulated and experimental settings, evaluating multiple models. Their approach achieved 86% accuracy in classifying the seven crystal systems and 77% accuracy in identifying the 230 space groups tested using the RRUFF dataset, outperforming the results reported by Vecsei *et al.* (2019 ▸).

2D diffraction patterns provide more detailed information than their 1D counterparts. While 1D integration collapses the intensity distribution onto a single axis, 2D patterns preserve spatial features such as spot positions and symmetry relationships that are essential for distinguishing complex structural variations. Consequently, researchers have increasingly utilized 2D diffraction patterns, including X-ray and electron diffraction, for crystal structure classification (Matinyan *et al.*, 2023 ▸; Yoo *et al.*, 2024 ▸; Gong *et al.*, 2025 ▸). Compared with a multi-layer perceptron, CNNs are more effective in extracting features from 2D image data (Potluri *et al.*, 2011 ▸; Driss *et al.*, 2017 ▸), motivating more work to use CNNs to study crystal structure classification. Ziletti *et al.* (2018 ▸) developed a deep learning approach to classify crystal structures by analyzing their simulated 2D diffraction patterns. They utilized a dataset comprising more than 100000 structures, including both pristine crystals and those with defects such as vacancies and atomic displacements. These structures were categorized into distinct crystal classes, each corresponding to specific space group Nos. 229, 227, 225, 221, 194, 166, 141 and 139. Limited to these specific space groups rather than all 230 space groups, they used a CNN model and achieved high classification accuracy on the test set. Chen *et al.* (2023 ▸) developed a machine-learning-based framework for the automated identification of seven crystal systems, not the 230 space groups, using approximately 500000 simulated electron diffraction patterns. They designed a custom CNN to capture physical parameters embedded in the diffraction patterns. They tested its predictive performance with one, two or three images or vector maps as inputs (corresponding to one, two or three diffraction patterns per case), observing that accuracy improves significantly when two or three diffraction patterns are input to the CNN compared with one diffraction pattern. The framework achieved high accuracy under noisy conditions and a 94% accuracy on experimental data from AuCu nanoparticle libraries comprising 17 datasets with cubic crystal systems.

While these prior studies have advanced crystallographic classification using machine learning techniques, several limitations persist. Methods relying on 1D diffraction patterns sacrifice the rich spatial and symmetry details inherent in 2D patterns, often exhibiting reduced accuracy on experimental datasets and lacking adaptability to structural complexities such as defects or deformations. Conversely, 2D diffraction patterns offer greater detail but are hindered by restricted orientation sampling (zone axis), or a narrow focus on crystal systems rather than the full 230 space groups. Our work bridges these gaps by leveraging synthetic 2D XRD patterns generated via the novel Auto Diffraction Pipeline, enabling classification across all seven crystal systems and 230 space groups with extensive orientation diversity. To investigate the effects of various zone axes (both observed and unobserved during training), isotropic lattice scaling, atomic substitutions and structural defects, our approach delivers a versatile, high-throughput framework for automated crystallographic analysis, minimizing the gap with experimental patterns and advancing materials discovery.

While the model is trained on specific zone axes, the primary objective is automated structure classification rather than orientation determination. Consequently, in an experimental setting where the structure and orientation are unknown, the user is not required to know or input the zone axis; the model implicitly learns to recognize crystallographic structures across various orientations.

## 2. Methodology for synthetic XRD dataset generation and deep learning classification

### 2.1. Data preparation

The dataset used in this study comprises crystallographic information files (CIFs) obtained from the Inorganic Crystal Structure Database (ICSD) (Belsky & Hellenbrandt, 2002 ▸). To ensure high data quality, incomplete or duplicated structures were removed, resulting in a total of 177432 CIFs. These CIFs were then categorized according to their symmetry operations into 230 distinct space groups and subsequently organized into seven crystal systems: cubic, hexagonal, trigonal, tetragonal, orthorhombic, monoclinic and triclinic. The distribution of CIFs across both the 230 space groups and the seven crystal systems is illustrated in the supporting information (Fig. A).

It was observed that the number of CIFs varied significantly among the 230 space groups, with some containing many more files than others. To achieve a more balanced distribution and reduce computational overhead, a threshold of 500 CIFs per space group was established. Specifically, for any space group with more than 500 files, a randomly sampled subset of 500 CIFs was selected, thereby reducing the overall dataset from 177432 to 52191 CIFs. Although some imbalance persists, this approach yields a more balanced representation compared with the original dataset. Furthermore, since natural distributions of crystal structures are inherently imbalanced, the resulting dataset remains computationally efficient for the deep learning model training and serves as a representative sample of the 230 space groups. To use CIFs for training and testing deep learning models while maintaining balanced representation across each space group, we partitioned the CIFs in every group into 70% for training, 20% for validation and 10% for testing. As a result, from the total of 52191 CIFs, we allocated 36462 for training, 10367 for validation and 5362 for testing.

**Figure 1:**
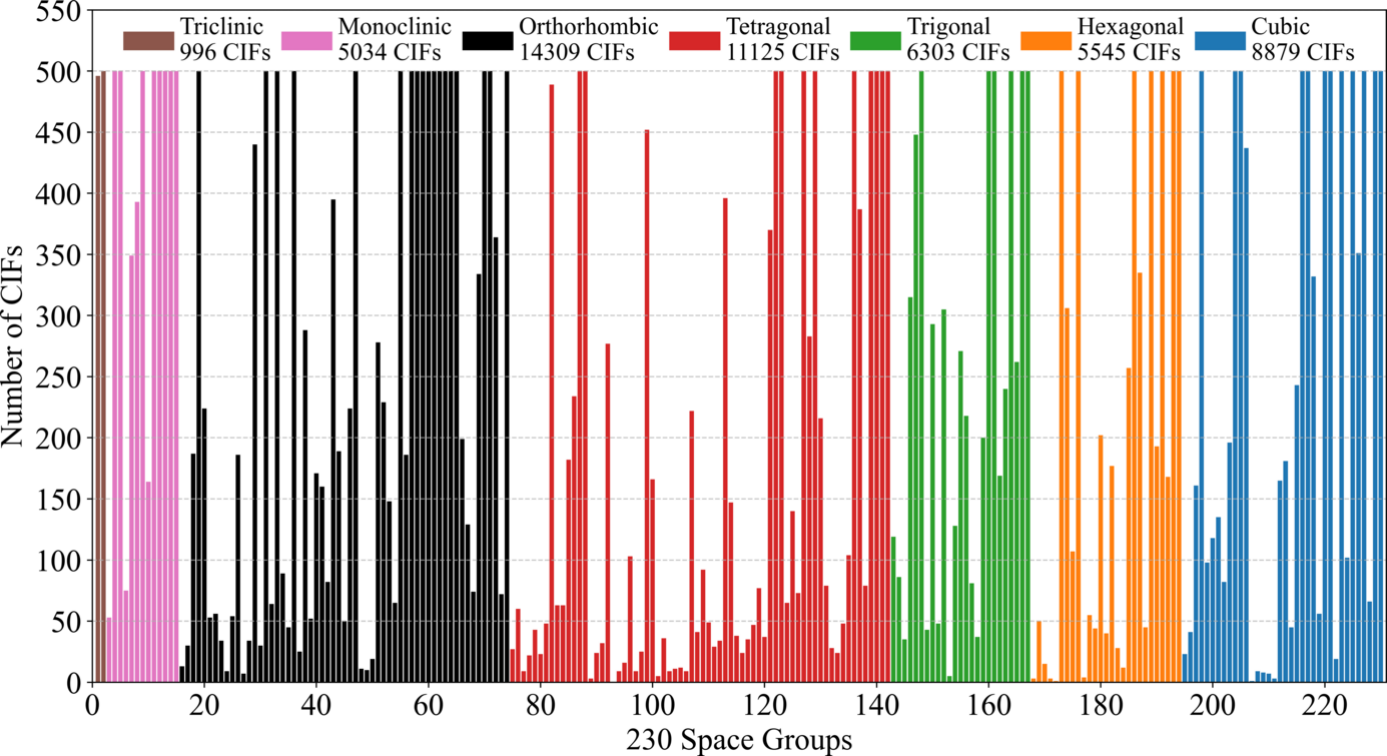
The distribution of 52191 CIFs, referred to as the ‘52k dataset’, across 230 space groups after applying a threshold of 500 files per space group. Different colors are used to represent the seven crystal systems, and the legend provides the number of CIFs associated with each crystal system.

Fig. 1 ▸ illustrates the distribution of these 52191 CIFs across the 230 space groups, with each of the seven crystal systems represented by a distinct color. Since this dataset, comprising 52191 CIFs, will be frequently referenced, we will refer to it as the ‘52k dataset’ for simplicity.

#### 2.1.1. Zonal axes and their role in 2D XRD pattern generation

In order to prepare datasets of simulated 2D XRD patterns for training deep learning models, after preparing the CIFs, the next step is to define the zone axes. In a crystal, every plane in real space corresponds to a vector in reciprocal space. A 2D diffraction pattern represents a plane in reciprocal space, encompassing all reciprocal-lattice vectors perpendicular to a specific direction, defined as the zone axis [*uvw*] (Cullity & Smoluchowski, 1957 ▸; He, 2018 ▸). The zone axis is fundamental because it determines which lattice planes contribute to the diffraction pattern, directly influencing the interpretation of the crystallographic data. The relevant equations and mathematical details are explained in Section 2.4. Our selection of zone axes was purposefully restricted to integers *u*, *v*, *w* in [0, 3] rather than employing uniform Euler angle sampling. This approach is motivated by the fact that XRD intensity and spot density are maximized along low-index zone axes, which correspond to planes with high atomic density. In contrast, arbitrary Euler angles frequently align with high-index irrational directions, resulting in sparse or weak diffraction patterns that are less effective for training symmetry classifiers (Williams & Carter, 1978 ▸; Cullity, 1986 ▸; Wheeler, 2006 ▸).

We imposed four primary constraints on these axes. First, we restricted the indices *u*, *v*, *w* to integer values in the range of 0 to 3 and randomly generated zone axes within this interval. Second, we excluded the zone axis [000] because it does not represent a valid crystallographic direction. Third, we selected only nonnegative values of *u*, *v*, *w*. Since [*uvw*] and its negative [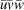] correspond to the same crystallographic axis, restriction to nonnegative values avoids duplication and ensures a simpler, more uniform dataset without sacrificing generality. Fourth, to further avoid generating identical 2D XRD patterns, only one representative zone axis was selected from sets of axes sharing the same direction, such as [001], [002] and [003], or [111], [222] and [333] *etc*.

Table 1 ▸ summarizes the zone axes selected for simulating 2D XRD patterns. Initially, we employed a single zone axis and trained a model based on this axis (model-1). Next, we expanded our approach to incorporate four zone axes (model-2), then ten zone axes (model-3) and finally 20 zone axes (model-4). By increasing the number of zone axes, we expanded the diversity and size of our dataset, potentially improving both model robustness and predictive performance. For instance, our 52k dataset contains 52191 CIFs. When employing 20 different zone axes, each CIF yields 20 distinct 2D XRD patterns. Consequently, this procedure produces 52191 × 20 = 1043820 synthetic 2D XRD patterns in total.

To validate the coverage of this approach, we analyzed the geometric distribution of the 20 selected zone axes (model-4). The analysis revealed an average nearest-neighbor angular separation of 13.03° and a global average separation of 44.32°. This confirms that our set achieves a local sampling resolution comparable to a dense 15° grid (approximately 216 orientations), but does so with significantly greater computational efficiency. While a dense 216-orientation grid would require processing over 11 million images, our strategic selection of 20 high-symmetry axes maintains the dataset at a manageable 1 million patterns without sacrificing the capture of crystallographically significant features. The angular relationships are visualized in Fig. B (supporting information).

### 2.2. Development of a Python-based pipeline for generating synthetic 2D XRD datasets

After preparing CIFs and identifying the zone axes, the next step is to generate synthetic 2D XRD patterns. To achieve this objective, we developed a Python-based framework called the Auto Diffraction Pipeline (ADP), which uses CIFs as input and produces synthetic 2D XRD spot patterns characteristic of single-crystal samples. This approach provides a reliable framework for overcoming the scarcity of experimental data, thereby facilitating more robust and effective machine learning applications in crystallography.

The pipeline employs the Cu *K*α emission line (λ = 1.5406 Å), and by transforming real-space coordinates into reciprocal space, as detailed in Section 2.4, it generates synthetic 2D XRD patterns under various conditions. These conditions include unrestricted zone axes, adjustable intensity profiles, isotropic lattice scaling, atomic substitutions and the presence of defects. The pipeline can also simultaneously produce 1D and 2D XRD datasets. It offers user-friendly operation and open-access availability via GitHub.

Additionally, the ADP is capable of generating 2D XRD patterns rotated in plane around the origin (*i.e.* the center of the diffraction pattern), independent of the *x* and *y* axes, at either random or specified angles. This rotation simulates variations in experimental setups or sample orientations, which often cause the diffraction pattern to rotate around the center point. To demonstrate the model’s robustness to such rotations, we conducted an experiment, the results of which are presented in Section 3.

All simulated diffraction patterns were exported at a fixed resolution of 321 × 322 pixels (100 d.p.i.) to maintain a uniform tensor size for CNN input. This standardization simplifies model design and training but limits adaptability to detectors with different pixel densities or geometries. In the present implementation, diffraction patterns are generated as ideal, noise-free, reciprocal-space projections to isolate the intrinsic representational capacity of the simulated data. Experimental artifacts such as background intensity, detector noise or beam non-uniformity are intentionally excluded; we plan to incorporate them in future extensions of the ADP.

**Figure 2:**
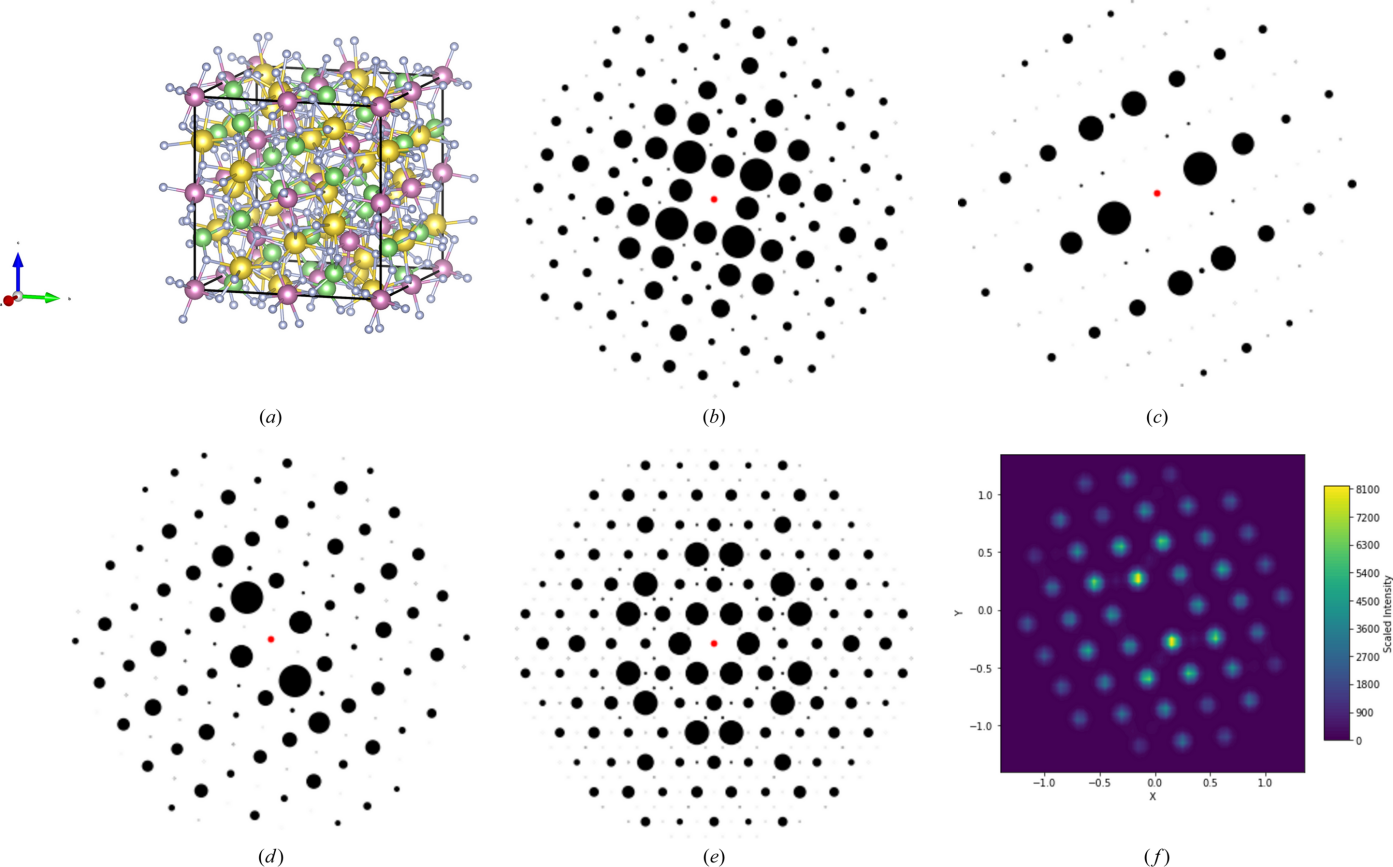
(*a*) A randomly selected ICSD CIF (ICSD_416927) with the chemical composition F_12_In_2_Li_3_Na_3_ possesses the cubic space group *Ia*3*d*. (*b*–*e*) Representative 2D XRD spot patterns generated for different zone axes using the ADP for the selected structure. (*f*) A Gaussian distribution intensity pattern of the structure for a random zone axis. (*b*) [100], (*c*) [023], (*d*) [101], (*e*) [111], (*f*) [210].

Fig. 2 ▸ presents 2D XRD patterns generated from a random CIF for multiple zone axes in the ADP. Fig. 2 ▸(*a*) depicts the atomic structure within a unit cell, Figs. 2 ▸(*b*)–2 ▸(*e*) show 2D XRD patterns with solid spot intensity profiles for different zone axes of the structure, and Fig. 2 ▸(*f*) illustrates a Gaussian intensity distribution. Only the solid spot patterns were used for training and testing the deep learning models described in this work.

### 2.3. CNN architecture and training

**Figure 3:**
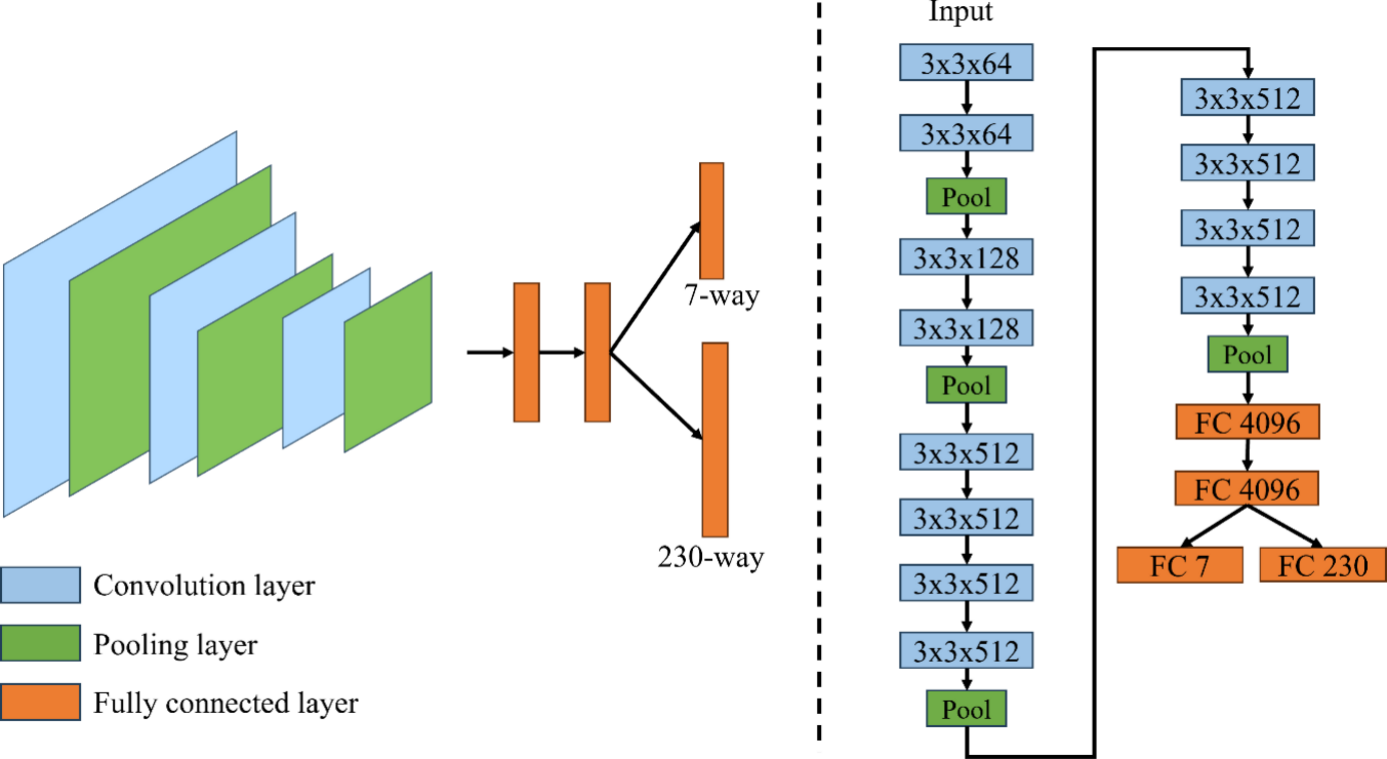
Architecture of the CNN used for 2D XRD pattern classification. The model consists of three main components: (1) a convolutional backbone with four blocks for feature extraction, each containing multiple convolutional layers and max pooling, (2) fully connected layers with 4096 hidden neurons for further feature refinement, and (3) classification heads for 230-way and 7-way crystal system classification. The model is trained using a dataset split in a 7:2:1 ratio for training, validation and testing, optimized with the Adam optimizer and with a learning rate of 0.0001 over 100 epochs.

We leverage the CNN to learn from the 2D XRD data. As shown in Fig. 3 ▸, the model consists of three parts, including the convolution + max pool backbone for feature extraction, fully connected layers for further extracting the information, and the classification layer for 230-way and 7-way classification.

In the convolution backbone, there are four blocks: each block consists of multiple convolution layers and a max pooling layer. We use a kernel with a size of 3 × 3 and increase the number of channels consistently from 64 to 512. There is a pooling layer after each convolution block for important feature selection. In the fully connected layer, we use two layers for feature extraction, each of which consists of 4096 hidden neurons. We have two classification heads, one for 230-way classification and another for 7-way classification.

We set the batch size as 128 and the number of workers (parallel subprocesses for data loading) as 8 to accelerate the data loading during the training process. We use Cross-Entropy as the loss function with the Adam optimizer to optimize our model and set the learning rate as 0.0001. The number of epochs for training our models is set as 100.

### 2.4. Mathematical modeling of XRD patterns

The diffraction of X-rays by a crystal is commonly explained using Bragg’s law. This principle states that when X-rays strike the crystal planes at an incident angle θ and are reflected at the same angle, a diffraction peak occurs if the Bragg condition is satisfied. The condition is expressed as (He, 2018 ▸)
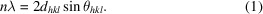
In this equation, λ represents the wavelength of the X-rays, 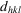 denotes the spacing between adjacent crystal planes (*d* spacing) calculated from the Miller indices (*hkl*) and the lattice parameters (*a*, *b*, *c*, α, β, γ), 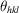 is the Bragg angle, and *n* corresponds to the order of reflection (Salgado *et al.*, 2023 ▸).

A zone is a collection of non-parallel planes within a crystal lattice that share a common parallel axis, known as the zone axis. The Miller indices of all planes in a given zone satisfy the following relationship (He, 2018 ▸):
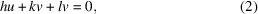
where [*uvw*] represents the direction of the zone axis and (*hkl*) denotes the Miller indices of the planes within the zone. This relationship mathematically links the orientation of the planes to the direction of the zone axis.

To generate 2D XRD patterns, it is necessary to transform the crystal lattice from real space into reciprocal space. The dimensions and geometry of a unit cell in reciprocal space are defined by three vectors, 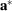, 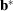 and 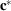, which are calculated as (He, 2018 ▸)
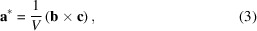

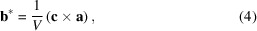

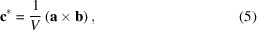
where *V* represents the volume of the crystal unit cell in real space, defined as
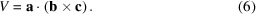
During XRD, each point in the reciprocal lattice corresponds to a reciprocal-lattice vector **H**, which describes the deviation between the incident and diffracted wavevectors. This vector is expressed as (He, 2018 ▸)
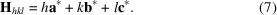
Thus, each point (*hkl*) in the reciprocal lattice corresponds to a set of lattice planes (*hkl*) in the real-space lattice, linking the diffraction phenomenon directly to the crystal’s geometric structure.

The next step involves calculating the Ewald sphere, also referred to as the reflection sphere. This geometrical construction visually represents the orientation of Bragg planes that satisfy the conditions for diffraction. The radius of the Ewald sphere is defined as 1/λ (He, 2018 ▸).

The subsequent step involves calculating the diffraction intensity at each reciprocal-lattice point for X-ray radiation by determining the structure factor, *F*(**H**), which depends on the atomic positions, 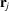. The structure factor is given by (Coleman *et al.*, 2013 ▸)
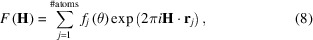
where 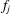 is the atomic scattering factor. 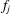 is computed using analytical approximations, parameterized for the specific atom type (Coleman *et al.*, 2013 ▸):
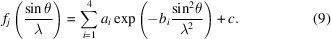
The diffraction intensity, 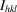, is derived from the product of the structure factor and its complex conjugate, 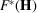, normalized by the number of atoms, *N*, in the simulation. Additionally, a Lorentz–polarization factor, Lp(θ), is applied to correct for the distribution of reciprocal-lattice points and the variation in scattered intensity due to non-polarized incident radiation. This factor is given by (Coleman *et al.*, 2013 ▸)
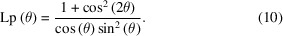
Thus, the XRD intensity is computed as (Coleman *et al.*, 2013 ▸)
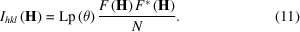
For the 2D diffraction simulations, reciprocal-lattice points satisfying the Ewald sphere condition were projected onto the detector plane defined by the selected zone axis. For each crystallographic direction 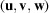, an orthogonal coordinate frame 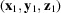 was established, where 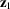 is parallel to the zone axis, and 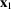 and 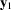 are generated as perpendicular unit vectors through cross-product operations. The reciprocal-lattice coordinates were transformed into this frame using the rotation matrix **R** to ensure consistent image orientation across all projections. Each diffracted reflection was rendered as a filled circular spot whose radius was proportional to the normalized reflection intensity, 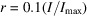, where 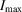 denotes the strongest reflection and 0.1 serves as a scale factor used to map relative intensities to visually interpretable spot sizes. Therefore, the rendered spot size serves strictly as a visual proxy for the diffraction intensity (structure-factor magnitude) and does not represent physical beam broadening or detector point-spread functions. A small red spot at the origin marks the direct beam position. No background, noise or instrumental broadening was added; consequently, the signal-to-noise ratio is effectively infinite. Because the input CIFs describe mathematically perfect structures, the generated patterns display discrete, sharply localized reciprocal-lattice points rather than the continuous intensity distributions typical of experimentally imperfect samples. These patterns provide analytical, noise-free visualizations of reciprocal-space geometry and intensity contrast determined purely by the crystal structure.

## 3. Results (space group and crystal system classification by deep learning)

### 3.1. Classification performance of model-1 trained on a single zone axis

Model-1 is our initial deep learning model, trained exclusively on 2D XRD patterns derived from a single zone axis, [100]. We used the 52k dataset of CIFs (70% for training, 20% for validation and 10% for testing), yielding a total of 52191 synthetic 2D XRD patterns (52191 CIFs × 1 zone axis). To evaluate model-1’s generalization, we generated additional 2D XRD patterns along the [010], [001] and [111] zone axes using the 10% test subset of CIFs, testing the model’s ability to handle orientations unseen during its [100] zone axis training.

Table 2 ▸ presents the weighted accuracy and top-3 accuracy of model-1 for classifying the seven crystal systems and 230 space groups. Weighted accuracy accounts for the varying number of 2D XRD patterns across the seven crystal systems and 230 space groups, assigning higher weight to systems or groups with more patterns to reflect their representation in the dataset. Top-3 accuracy measures the percentage of cases where the correct crystal system or space group is among the model’s top three predicted classes.

**Figure 4:**
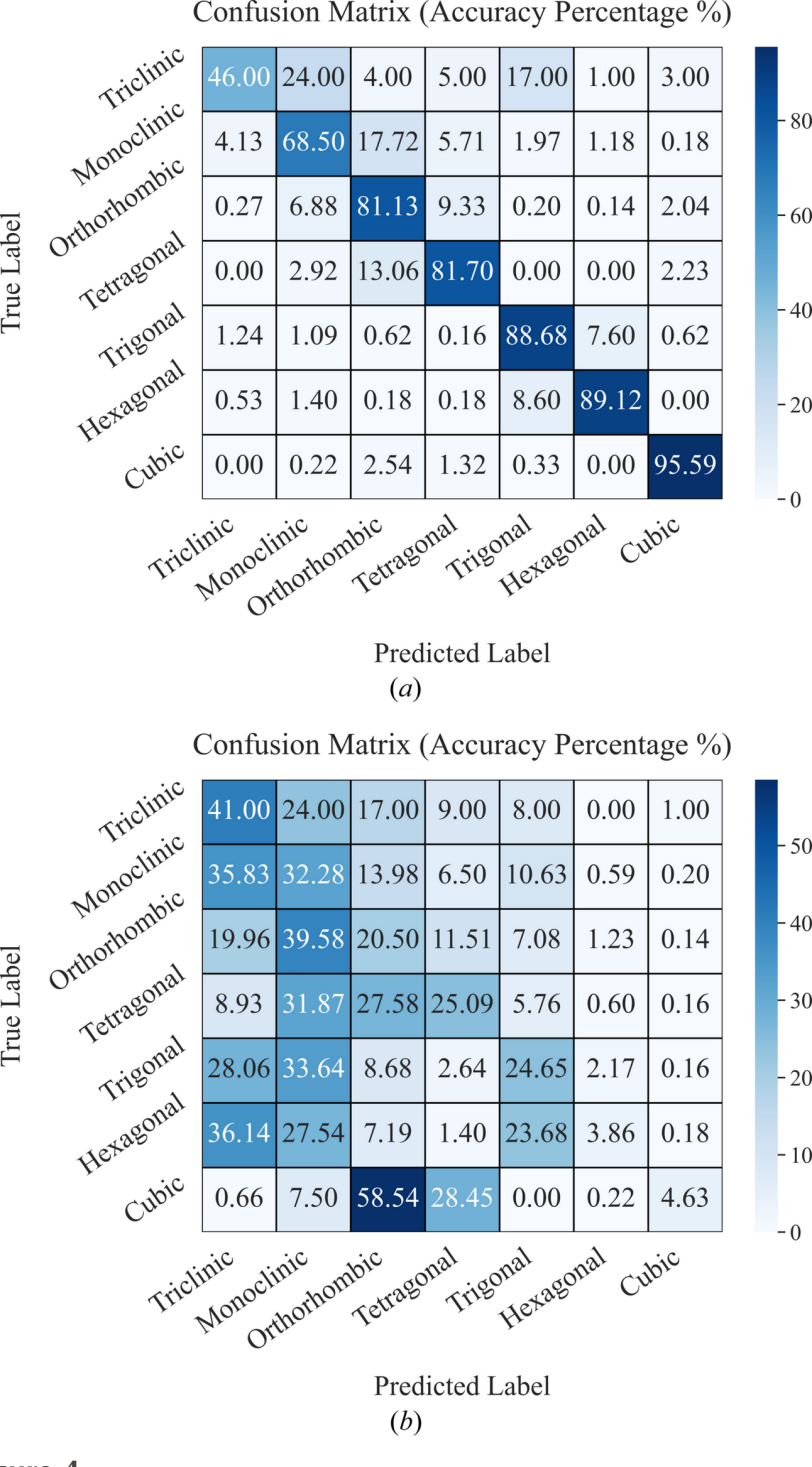
Confusion matrices illustrating model-1’s performance in classifying the seven crystal systems for (*a*) the [100] zone axis and (*b*) the [111] zone axis.

Model-1 was trained using 2D XRD patterns from the [100] zone axis and then tested on the [111], [100], [010] and [001] zone axes. As expected, Table 2 ▸ shows that model-1 achieved high accuracy for both crystal system and space group classification when tested on [100] zone axis patterns, as the training and testing used the same [100] zone axis. Fig. 4 ▸(*a*) displays the confusion matrix for classifying the seven crystal systems, illustrating that higher-symmetry crystals tend to yield higher classification accuracy. Notably, the model attains 95.59% accuracy for cubic systems. In the confusion matrix, each row represents the true category of the samples, while each column represents the category predicted by the model. The cells along the diagonal show the percentage of correct predictions, while the off-diagonal cells display the percentage of misclassifications.

Table 2 ▸ reveals that model-1 demonstrates lower testing accuracy in descending order for the [010], [001] and [111] zone axes compared with the [100] zone axis when classifying seven crystal systems and 230 space groups. This trend arises because the tested zone axis [010] and the trained zone axis [100] are crystallographically equivalent in cubic and tetragonal crystal systems, resulting in more 2D XRD patterns with similar symmetry in the test dataset. In contrast, the tested zone axis [001] and the trained zone axis [100] are equivalent only in the cubic system, while the tested zone axis [111] has no crystallographic equivalence with the trained zone axis [100], leading to the lowest accuracy. This trend is illustrated in Fig. C (supporting information), which presents the confusion matrices for model-1 evaluated on the trained zone axis [100] and the tested zone axes [010], [001] and [111]. Model-1 achieves the lowest testing accuracy for the [111] zone axis because the [100] and [111] zone axes belong to distinct crystallographic zone families and are not equivalent across the seven crystal systems. Fig. 4 ▸(*b*) illustrates the confusion matrix for the seven crystal systems’ classification when model-1 is tested on the [111] zone axis, clearly highlighting the occurrence of misclassifications.

Another insight derived from Table 2 ▸ is that the accuracy of classifying the seven crystal systems consistently exceeds that of classifying the 230 space groups. This difference can be attributed to the fact that predicting among seven classes (crystal systems) is inherently less challenging than predicting among 230 classes (space groups). Figs. C and D (supporting information) illustrate model-1’s performance in classifying the seven crystal systems, as evaluated through confusion matrices based on accuracy percentages and the number of correct predictions. Additionally, Fig. E (supporting information) depicts the model’s performance in classifying the 230 space groups, with accuracy reported for each space group.

### 3.2. Classification performance of models-2, -3 and -4 trained on four, ten and 20 zone axes

To enhance the generalization, performance and robustness of deep learning models in predicting the classification of the 230 space groups and seven crystal systems, we trained the models on larger synthetic 2D XRD datasets. Specifically, model-2 was trained using four zone axes, model-3 was trained on ten zone axes and model-4 was trained on 20 zone axes. These models were developed using the 52k dataset, split in a 7:2:1 ratio (training: validation: test), consistent with the approach used for model-1, as detailed in Table 1 ▸. Consequently, three distinct test datasets were created, containing four, ten or 20 zone axes corresponding to the training zone axes. Each trained model was evaluated on all three test sets to comprehensively assess its performance in classifying the 230 space groups and seven crystal systems.

Table 3 ▸ presents the weighted accuracy and top-3 accuracy for model-2, model-3 and model-4, evaluating their performance in classifying (*a*) the seven crystal systems and (*b*) the 230 space groups. Consistent with the findings for model-1 (Table 2 ▸), the classification accuracy for the seven crystal systems surpasses that for the 230 space groups.

The results presented in Table 3 ▸ demonstrate that when a model, such as model-2, is trained on a limited number of zone axes (in this case, four) it achieves high accuracy in classifying the seven crystal systems and 230 space groups when tested on the same zone axes as used for training. However, model-2’s accuracy decreases when evaluated on test sets containing a broader range of zone axes, such as ten or 20. This reduction in performance is anticipated as the model encounters unfamiliar 2D XRD patterns derived from zone axes not seen during training. In contrast, model-4 demonstrates a similar accuracy range when evaluated on test datasets containing four, ten and 20 zone axes, achieving a weighted accuracy of approximately 83% for classifying the seven crystal systems and 60% for the 230 space groups. This performance is attributed to the diversity of model-4, which is trained on a dataset encompassing 20 zone axes, including those present in the four-zone and ten-zone datasets.

Several factors contribute to the lower accuracy in classifying the 230 space groups compared with the seven crystal systems. One key reason is Friedel’s law. This law states that the intensities of reflections *hkl* and their Friedel mates 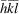 are equal, as they depend on the square of the structure factor’s magnitude (De Graef & McHenry, 2012 ▸). Consequently, this equality obscures whether a crystal possesses an inversion center, making it challenging to distinguish centrosymmetric from non-centrosymmetric structures solely on the basis of their diffraction patterns (Ziletti *et al.*, 2018 ▸). To examine how inversion symmetry influences space group classification, we divided the 230 space groups into two disjoint sets: centrosymmetric (CS, 92 space groups) and non-centrosymmetric (non-CS, 138 space groups) (Tückmantel, 2021 ▸). We then evaluated the classification accuracy of model-4 when tested across 20 distinct zone axes.

The CS groups correspond to indices [1, 3–9, 16–46, 75–82, 89–122, 143–146, 149–161, 168–174, 177–190, 195–199, 207–220], and the remaining indices correspond to non-CS groups. For each model prediction, both the predicted and true labels were mapped to these categories, yielding a reduced 2 × 2 confusion matrix that isolates errors related specifically to inversion symmetry. In this reduced matrix, the diagonal elements represent predictions where the model correctly identifies whether a structure is CS or non-CS. The off-diagonal elements, in contrast, correspond to cases where the model incorrectly assigns centrosymmetry, for example, predicting a non-CS structure as CS, or vice versa. Because this reduction evaluates only the presence or absence of an inversion center, any misclassification that still preserves the correct inversion property (for instance, predicting one CS group instead of another) remains counted as correct in this simplified evaluation.

**Figure 5:**
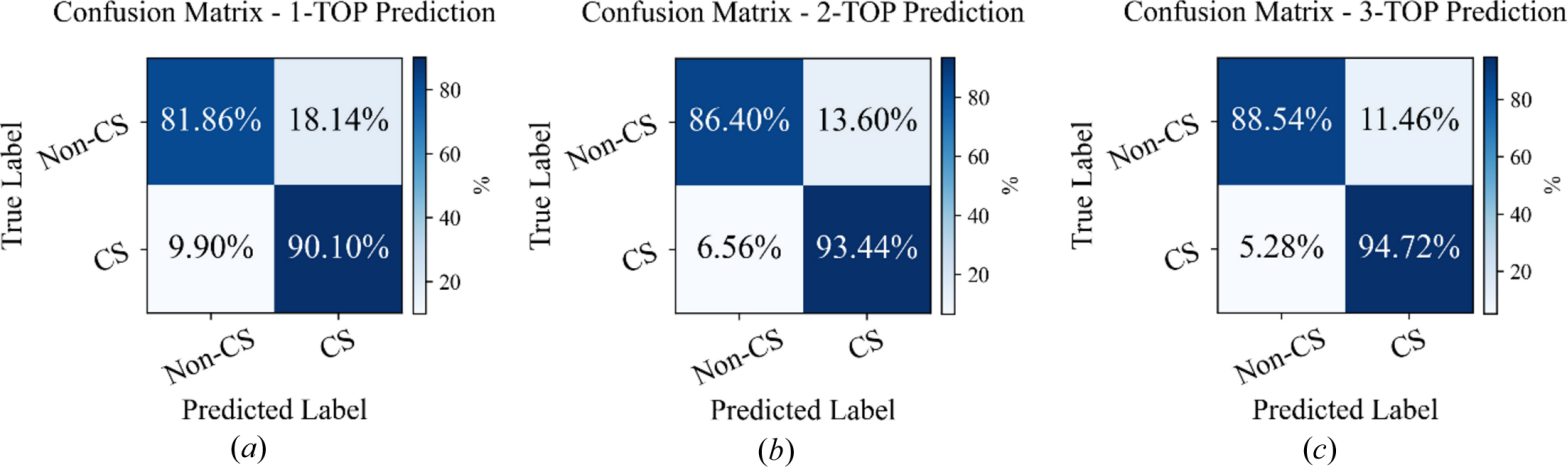
Confusion matrices of the mean accuracy of classifying centrosymmetric and non-centrosymmetric space groups using (*a*) top-1 prediction, (*b*) top-2 prediction and (*c*) top-3 prediction. Each 2 × 2 matrix is derived by reducing the full 230 × 230 space group confusion matrix into centrosymmetry categories. The 230 space groups were grouped into CS (92 groups) and non-CS (138 groups) classes according to *International Tables for Crystallography* (Tückmantel, 2021 ▸). The figure presents these reduced matrices for top-1, top-2 and top-3 predictions, revealing that most misclassifications stem from confusion across the CS/non-CS boundary. This indicates that inversion symmetry ambiguity constitutes the primary source of error in the model’s space group predictions.

Fig. 5 ▸ presents the confusion matrices for the two categories, CS and non-CS crystals, based on mean accuracy for (*a*) top-1, (*b*) top-2 and (*c*) top-3 prediction. The off-diagonal elements indicate misclassifications between the two categories. It is evident that the misclassification rate for the top-1 prediction [Fig. 5 ▸(*a*)] exceeds that for the top-2 and top-3 predictions [Figs. 5 ▸(*b*) and 5 ▸(*c*)]. This suggests that, under the influence of Friedel’s law, the model struggles with the initial prediction but improves in the second and third predictions. To address such challenges, two strategies can be proposed: first, considering multiple predictions (beyond the top-1) to enhance accuracy, and second, analyzing diffraction data from multiple zone axes for each material, rather than relying on a single zone axis, to improve crystal classification, as demonstrated by Chen *et al.* (2023 ▸).

Another factor contributing to the confusion in deep learning models classifying the 230 space groups is the presence of enantiomorphic space groups, comprising 11 pairs (22 space groups) that represent mirror images of each other, like left and right hands. These groups are chiral, lacking inversion centers or mirror planes, and form a subset of the non-CS space groups (Ha & Allewell, 1997 ▸). In 2D XRD, their patterns appear identical due to Friedel’s law (Goodman & Secomb, 1977 ▸; Flack & Bernardinelli, 2008 ▸).

**Figure 6:**
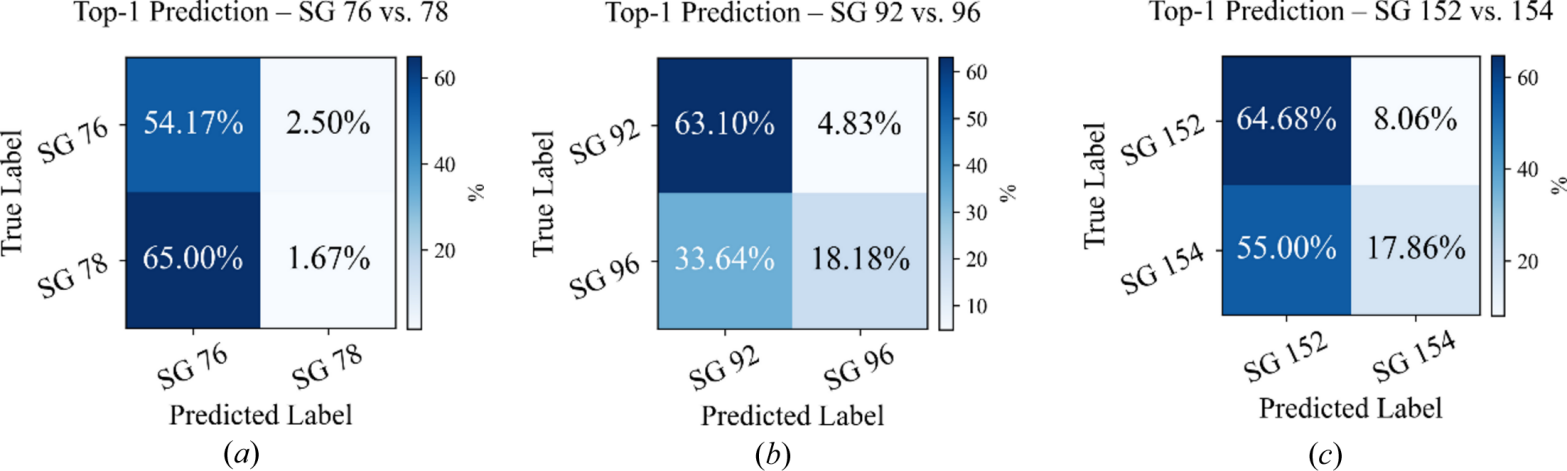
Confusion matrices of model-4 performance for three enantiomorphic space group pairs, (*a*) space groups 76 and 78, (*b*) space groups 92 and 96, and (*c*) space groups 152 and 154, highlighting misclassification challenges due to chirality.

In Fig. 6 ▸, the confusion matrices presented for three of the 11 enantiomorphic pair groups, 76 and 78, 92 and 96, and 152 and 154 [with the remaining pairs available in Fig. F (supporting information)], clearly illustrate the impact of enantiomorphism on the performance of model-4 when tested on 20 zone axes. For these three pairs, the matrices reveal a noticeable percentage of misclassification between the enantiomorphic counterparts. This effect is evident in the off-diagonal elements of the matrices; for example, misclassification rates between space groups 76 and 78 reach 2.5% and 65%. Such misclassifications are due to the inherent similarity in the symmetry features of enantiomorphic pairs. These cases exemplify the geometric ambiguity inherent to single-projection diffraction: distinct space groups may yield nearly identical 2D spot distributions under specific orientations, emphasizing that a single zone axis observation cannot uniquely constrain the full space group symmetry.

**Figure 7:**
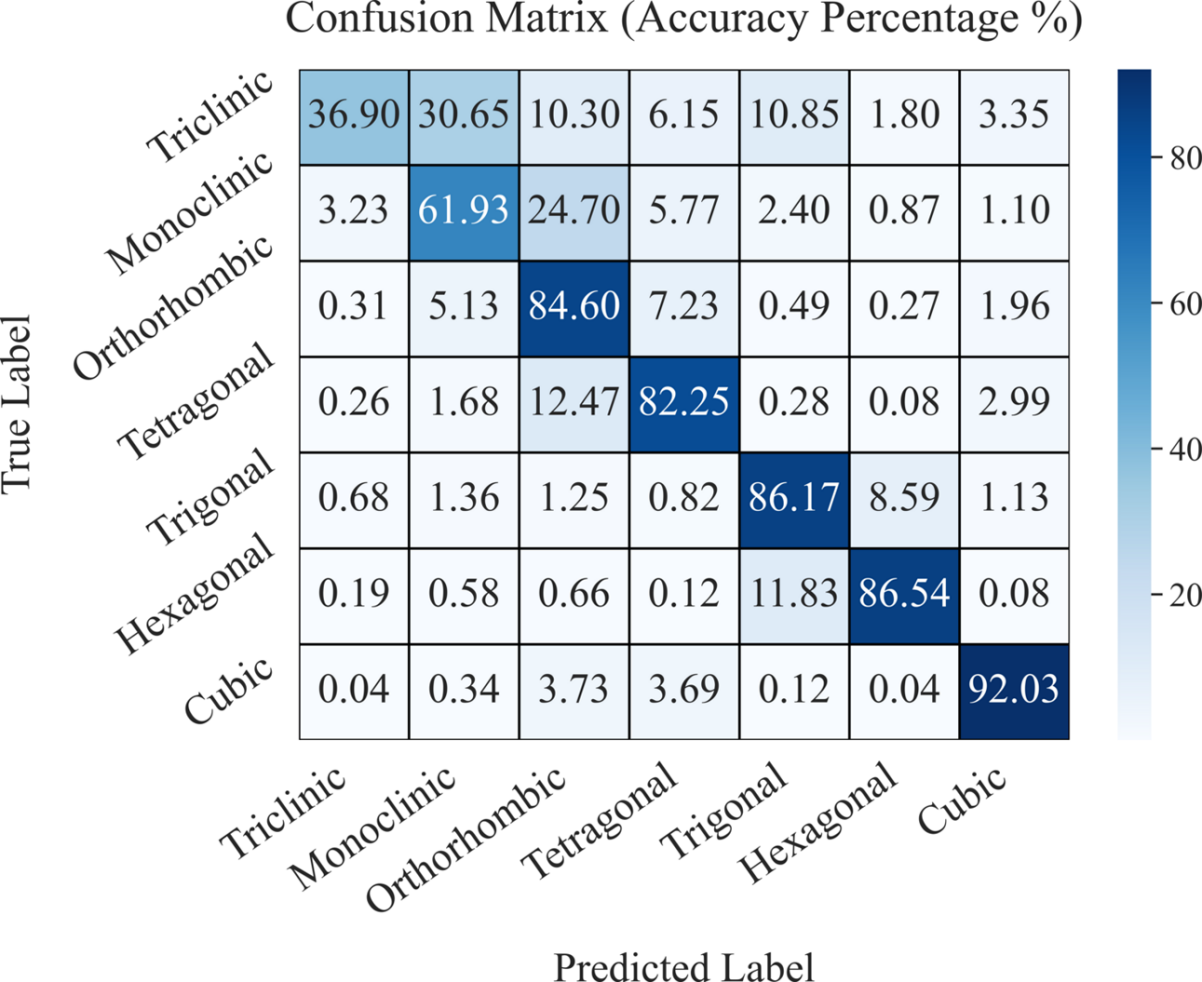
Confusion matrix illustrating the percentage accuracy for predicting seven crystal systems with the model trained on 20 zone axes and tested on the corresponding 20 zone axes dataset.

Fig. 7 ▸ presents the confusion matrix depicting the percentage accuracy of classifying the seven crystal systems using model-4, tested on the corresponding 20-zone test dataset. Model-4 demonstrates the highest classification performance for the cubic crystal system, achieving an accuracy of 92.03%. This superior performance can be attributed to the high symmetry of cubic crystals compared with other crystal systems. Following the cubic system, the hexagonal, trigonal, orthorhombic and tetragonal systems exhibit classification accuracies ranging from approximately 82% to 87%.

In contrast, the monoclinic and triclinic systems, which have lower crystallographic symmetry, yield the lowest accuracies at 61.93% and 36.9%, respectively. The relatively poor performance for the triclinic system can be further explained by the limited number of training samples, as it comprises only two out of the 230 space groups, leading to insufficient data for effective model training and validation. Additional confusion matrices illustrating the classification performance of the seven crystal systems across all test datasets and trained models are provided in Figs. H(1–9) and I(1–9) (supporting information).

**Figure 8:**
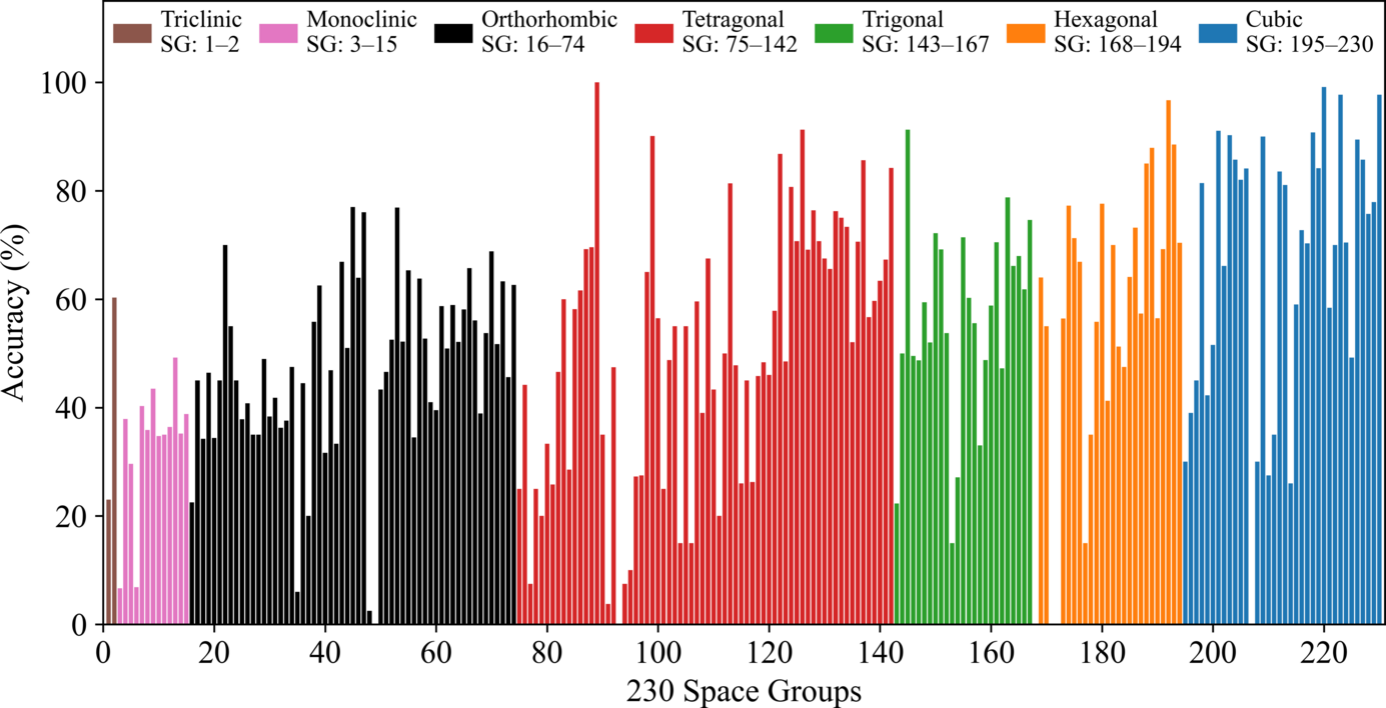
Classification accuracy for each of the 230 space groups using model-4 trained and tested on 20 zone axes.

Fig. 8 ▸ presents the classification accuracy for each of the 230 space groups when model-4 was tested on the corresponding 20-zone-axis test dataset. The *x* axis lists the space groups numerically from 1 to 230, while the *y* axis shows the percentage accuracy for each space group. Each bar is color-coded to reflect its corresponding crystal system among the seven crystal systems. A closer look at the prediction results shows that certain space groups exhibit notably lower accuracy. One primary reason for this outcome is the very low number of CIFs available for these groups, which significantly restricts the model’s ability to learn their distinct characteristics. To support this idea, Tables A and B (supporting information) indicate that 70 space groups are represented by between 0 and 50 CIFs, with an average accuracy of 37.32%. In contrast, 66 space groups have between 450 and 500 CIFs, achieving an average accuracy of 62.37%. This clearly demonstrates that, in general, a higher number of CIFs per space group leads to greater classification accuracy.

However, in this study, as some space groups in nature have very few CIFs while others have a significantly higher number, we applied a threshold of 500 CIFs for each space group to maintain better balance across space groups. We did not perform any augmentation to increase the number of CIFs in space groups with fewer than 500 CIFs. In future work, we plan to introduce noise and implement augmentation tech­niques to explore their effects on classification performance. Moreover, the classification accuracy for each of the 230 space groups, when models-2, -3 and -4 were tested on the different zone axis test datasets, is presented in Fig. J(1–9) (supporting information).

As previously discussed, the diffraction pattern can undergo in-plane rotation around the origin due to variations in experimental setups or sample orientations. To ensure that our deep learning models are robust to such rotations, we randomly selected 10000 CIFs from the training dataset and applied random rotations to their corresponding 2D XRD patterns for the [100] zone axis. We compared the classification accuracy of model-4 on these rotated patterns with that of their original patterns used in training. The model maintained consistent prediction accuracy regardless of rotation angle, as shown in Table C (supporting information).

### 3.3. Classification performance on unseen zone axes

To examine the trained models’ ability to generalize beyond training data and assess their performance on novel input scenarios, we conducted a new experiment. In this test, we introduced four unseen zone axes [131], [211], [011] and [120], which were not included in the datasets used for training models. For this experiment, we employed model-4, which was trained on the most diverse dataset. A test dataset of unseen 2D XRD patterns was generated from the test subset of the 52k dataset by applying the four unseen zone axes. This resulted in a total of 21448 unseen 2D XRD patterns, calculated as 5362 test CIFs × 4 unseen zone axes.

Table 4 ▸ presents the weighted accuracy and top-3 accuracy achieved by model-4. These results show the classification performance for seven crystal systems and 230 space groups. At first glance, it is evident that the accuracy for classifying the seven crystal systems is higher than that for classifying the 230 space groups. This is because predicting among seven options is easier than among 230 options, as previously discussed.

According to Table 4 ▸, the weighted accuracy for the unseen zone axes [211], [011] and [120] falls within a consistent range of approximately 60% to 65% (with top-3 accuracy ranging from 81% to 84%) for classifying the seven crystal systems, and 37% to 39% (with top-3 accuracy from 54% to 58%) for classifying the 230 space groups. Consequently, it is evident that the classification accuracy for unseen zone axes is lower than that for seen zone axes, as the tested unseen zone axes are not represented in the training dataset. This suggests that model accuracy could be further improved in future work by incorporating a broader range of zone axes into the training dataset.

In contrast, the accuracy for the unseen zone axis [131] is markedly lower than that observed for the other axes. The reason is that, among the 20 zone axes used to train model-4, there are no symmetrically equivalent directions (*i.e.* no same zone families) that correspond to the unseen zone axis [131], while the unseen zone axes [211], [011] and [120] have equivalent zone families present in the training set. Therefore, it can be concluded that a training dataset encompassing a broader range of zone axes may enhance classification accuracy for unseen zone axes, as it increases the likelihood that equivalent zone families are present in the training data. Table D (supporting information) provides a detailed overview of these symmetrically equivalent zone families.

**Figure 9:**
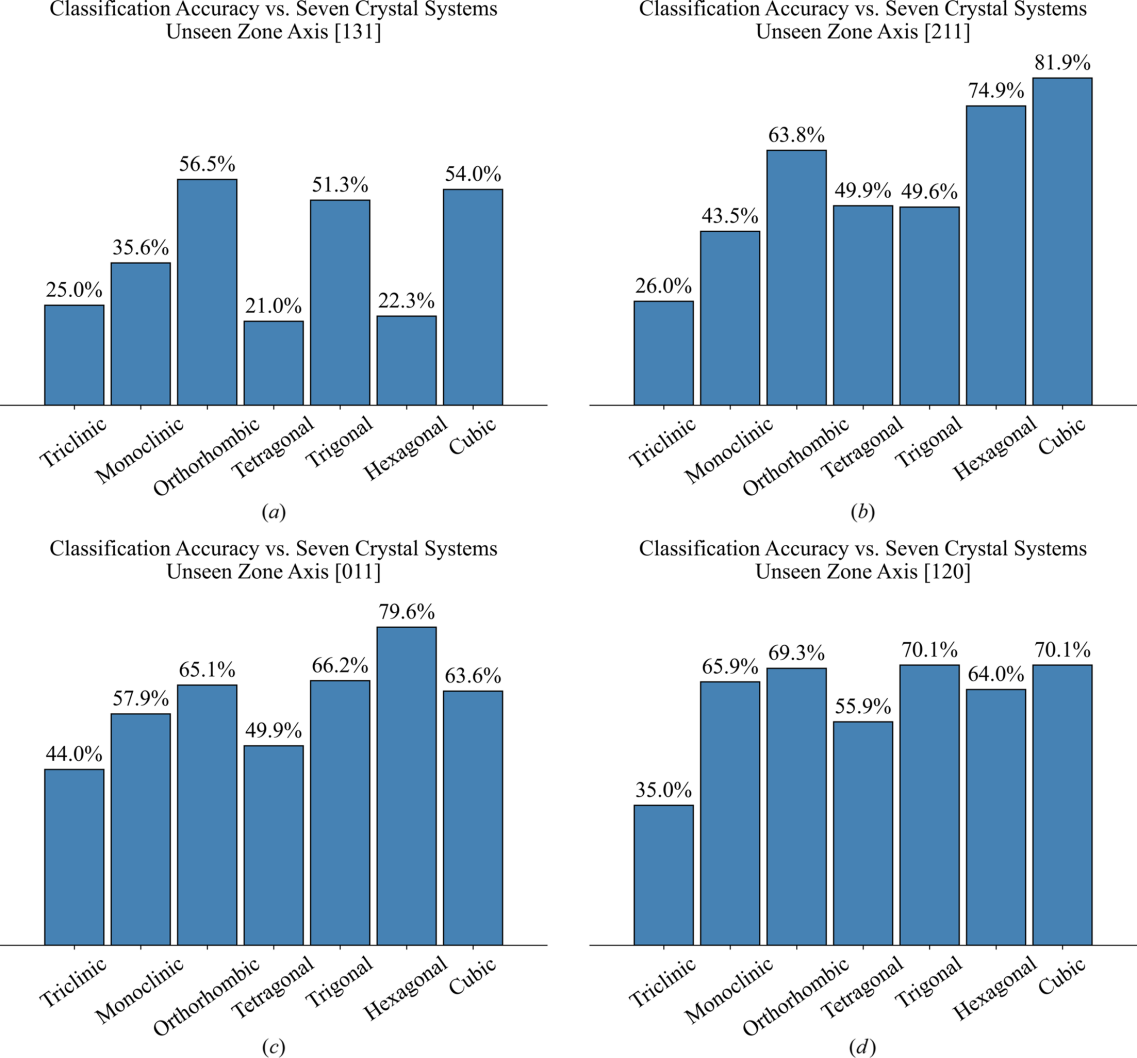
Classification accuracy of the proposed model-4 for each crystal system, evaluated on unseen zone axes: (*a*) [131], (*b*) [211], (*c*) [011] and (*d*) [120].

Fig. 9 ▸ presents model-4’s classification accuracy for seven crystal systems evaluated on unseen zone axes (*a*) [131], (*b*) [211], (*c*) [011] and (*d*) [120]. The triclinic system exhibits the lowest accuracy, due to its inclusion of only two space groups and consequently the fewest CIFs in the training dataset. In the unseen zone [131], we observe relatively low accuracy for the tetragonal, trigonal, hexagonal and cubic systems. However, in the other unseen zone axes [211], [011] and [120], these four crystal systems have the same zone families in the training dataset [refer to Table D (supporting information)], which in turn markedly increases their prediction accuracy, as illustrated in Figs. 9 ▸(*b*), 9 ▸(*c*) and 9 ▸(*d*). In addition, Fig. K (supporting information) presents the classification accuracy for each of the 230 space groups under the unseen zone axes [211], [011], [120] and [131]. These findings underscore the influence of having the same family of zone axes on the classification performance and highlight the importance of training data diversity in enhancing the robustness of the models. Each of the seven crystal systems has its distinct set of symmetry operations and equivalent planes. For example, the cubic system, characterized by the highest symmetry among the seven crystal systems, has the greatest number of equivalent orientations. Consequently, when model-4 is evaluated using unseen zone axes, cubic systems inherently offer the highest likelihood of encountering similar diffraction patterns across equivalent orientations.

### 3.4. Classification performance under isotropic lattice scaling conditions for cubic crystals

In the next phase of the study, the robustness of model-4 was evaluated under isotropic lattice scaling conditions to assess its sensitivity to changes in unit-cell size. These conditions involved scaling the lattice constants of cubic unit cells, which induced positional shifts in diffraction spots within 2D XRD patterns, to ensure the model learns the relative positions of diffraction spots in the 2D patterns rather than merely memorizing the absolute *x*–*y* coordinates from the training dataset. In this manner, uniform isotropic scaling was applied to the lattice vectors by multiplying the lattice parameters *a*, *b* and *c* by a common factor, thereby simulating proportional volumetric expansion or contraction with strain levels of +5% and +10% (expansive), 0% (neutral), and −5% and −10% (compressive). The unit cells, extracted from the training dataset, were cubic structures. 2D XRD patterns were simulated for two zone axes, [100] and [111], across these five isotropic lattice scaling conditions. The test dataset consisting of these simulated patterns was then evaluated using model-4 to classify the seven crystal systems and 230 space groups. In total, 60380 test patterns were generated, calculated as 6038 cubic structures from the training dataset × 2 zone axes × 5 strain conditions. The compressive strain conditions, in particular, serve to mimic the volumetric contraction observed in materials subjected to high-pressure environments.

**Figure 10:**
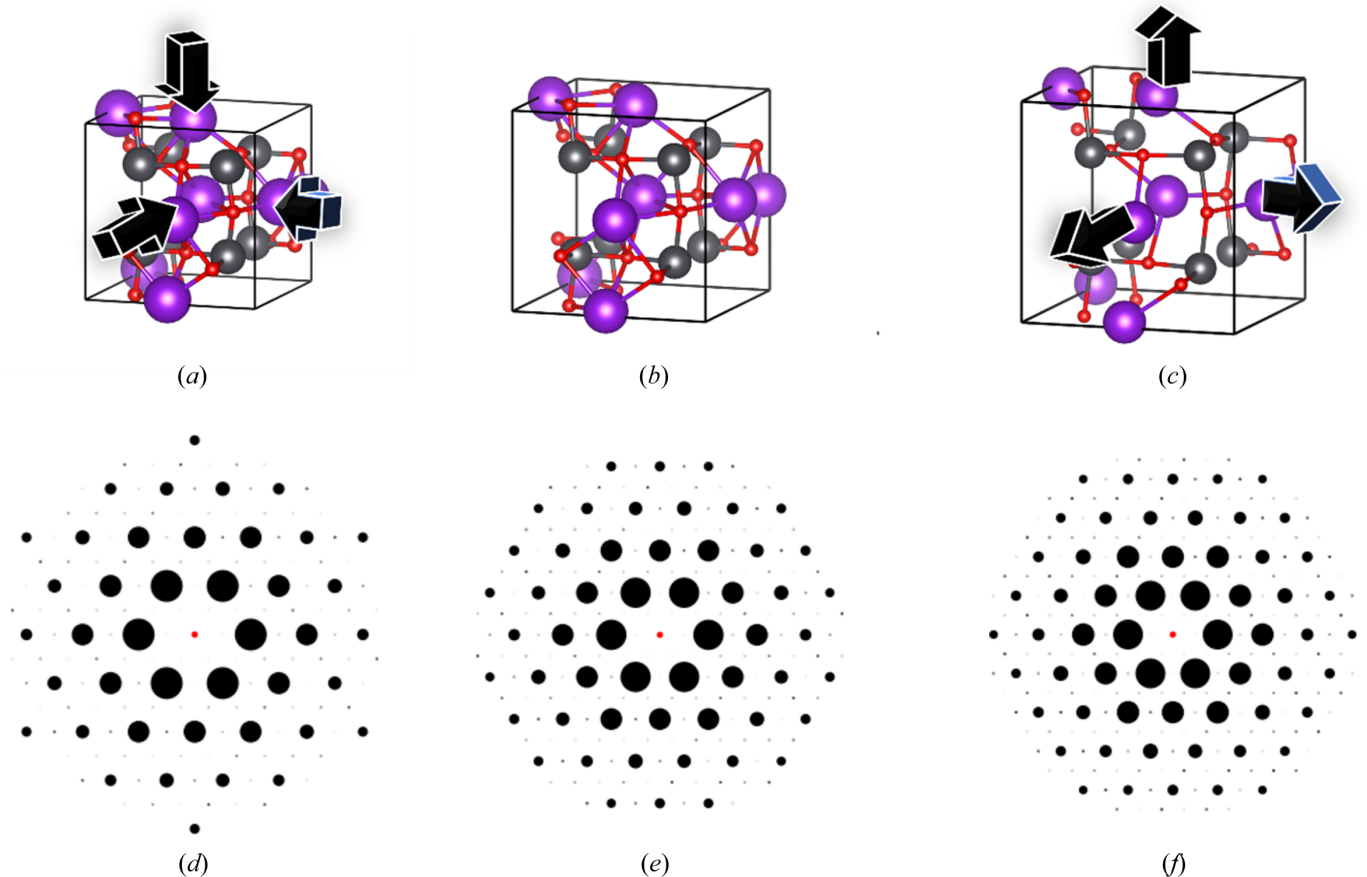
Isotropic lattice scaling along the **x**, **y** and **z** directions for ICSD_001412, demonstrating strains of (*a*) −10%, (*b*) 0% and (*c*) +10%. (*d*–*f*) Corresponding 2D XRD patterns for strains of −10%, 0% and +10% in the same material, along zone axis [111].

Under isotropic lattice scaling, deformation occurs equally in all three directions. Consequently, the symmetry remains unchanged, meaning that after applying isotropic lattice scaling the crystal system remains constant (cubic), and the space group remains identical to its state prior to loading. Fig. 10 ▸ illustrates the translation in the diffraction spot patterns under 0% loading, −10% compression and +10% expansion for the zone axis [111]. The 2D XRD patterns exhibit an expansion when subjected to compressive loading and a contraction under expansive loading. This behavior is explained by the inverse relationship between real space and reciprocal space, which is discussed in detail in Section 2.4.

As shown in Table 5 ▸, model-4 achieved high classification accuracy for both the seven crystal systems and the 230 space groups under isotropic lattice scaling. However, as the degree of expansion or compression increases, the classification accuracy for both crystal systems and space groups gradually decreases. For example, along the [100] zone axis, without isotropic lattice scaling, the weighted accuracy is 98.97% for crystal system classification and 94.25% for space group classification. Under −10% compression, these values drop to 94.35% and 76.60%, respectively, while under +10% expansion, the accuracy reaches 95.36% and 81.02% for crystal system and space group classification, respectively.

Isotropic lattice scaling applied to unit cells in real space induces radial translations in the positions of diffraction spots within 2D XRD patterns. In these radial translations, the relative ratios of spot positions remain constant, which explains the high classification accuracy reported in Table 5 ▸ across various percentages of expansion and compression. However, as the magnitude of expansion or compression increases, the classification accuracy for the seven crystal systems and 230 space groups gradually decreases. This decline occurs because greater strains cause the diffraction spot patterns to deviate further from those in the training dataset, leading to increased misclassification as the model struggles to generalize to these altered patterns.

### 3.5. Classification performance on unit cells with atomic substitutions

**Figure 11:**
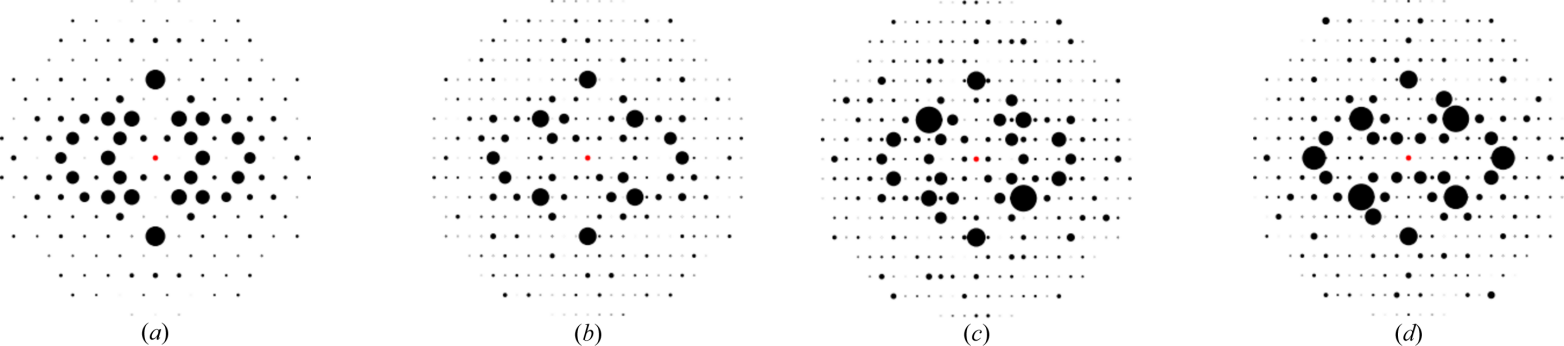
2D XRD patterns for the K_8_P_8_Ti_8_O_4O_ sample under (*b*) 10%, (*c*) 25% and (*d*) 50% atomic substitution [(*a*) 0% substitution]. The chemical compounds after substitution are (*b*) K_8_V_1_Ge_1_Ce_1_As_1_Rb_1_P_7_F_1_Ti_8_O_34_, (*c*) Ag_1_K_4_Co_1_Xe_2_Ca_1_Nd_1_Mn_1_Rh_1_Ar_1_F_1_Cr_1_Y_1_I_1_Si_1_Be_1_V_1_P_6_Ti_8_O_24_, (*d*) H_1_He_2_Co_4_K_4_Be_1_O_16_Ca_1_Zr_2_P_4_Mg_1_Ga_3_Ge_2_Mo_2_Na_1_Zn_1_Kr_2_Rh_1_Xe_1_Cu_1_Se_1_Ar_1_Ne_1_Ti_4_. While the lattice constants remain unchanged, the intensity of the diffraction spots varies with increasing substitution.

In this section, we evaluated the robustness of model-4, trained on the dataset with the highest diversity, under conditions of atomic substitution within the unit cell. These substitutions affect the structure factor of the crystal planes, consequently adjusting the intensity of the diffraction spots (brightness) and introducing a form of noise or perturbation in the 2D XRD patterns. The purpose of this evaluation is to verify that the trained model learns structural information from diffraction spot positions and arrangements, rather than relying solely on the intensity of the spots, which can change due to atomic substitutions, and to assess the impact of noise on the model’s predictive performance. As illustrated in Fig. 11 ▸, substituting 0%, 10%, 25% and 50% of the atoms in a sample unit cell significantly influences the intensity of diffraction spots.

To prepare the test datasets for this experiment, we established a criterion ensuring that, for a substitution of 1% of the atoms in the unit cell, the unit cell comprised at least 100 atoms. On the basis of this requirement, we extracted 6305 CIFs from the training subset of the 52k dataset, which includes all classes. Subsequently, we performed random substitutions of 0%, 1%, 2%, 5%, 10%, 25% and 50% of the atoms in these selected structures, selecting replacement elements from the periodic table. The ADP was then employed to generate 2D XRD patterns along the [100] and [111] zone axes for each substituted structure. This process yielded a total of 88270 synthetic 2D XRD patterns for testing, calculated as 6305 CIFs × 2 zone axes × 7 substitution levels.

We used model-4 to test the generated test dataset. Table 6 ▸(*a*) presents the classification accuracy for the seven crystal systems and 230 space groups, under varying levels of atomic substitutions (0%, 1%, 2%, 5%, 10%, 25% and 50%). For the crystal system classification for zone axis [100], the weighted accuracy at 0% atomic substitution is notably high at 87.66%, indicating robust performance of the model in distinguishing crystal systems in the absence of substitutions. However, as atomic substitutions are introduced, the weighted accuracy declines, dropping to 62.55% at 1% substitution and further to 57.32% at 50% substitution. The top-3 accuracy for crystal systems remains relatively stable, starting at 99.64% at 0% substitution and decreasing to 91.53% at 50% substitution. This suggests that, despite the absence of noise in the training datasets, the model maintains high accuracy in classifying the seven crystal systems among its top-3 predictions, even as noise and perturbations increase in the 2D XRD patterns.

The classification of crystal systems relies on the model’s ability to discern the relationship between lattice constants in real space and the corresponding spot positions in reciprocal space within 2D XRD patterns. Although increasing atomic substitutions introduce intensity variations, noise and perturbations in these patterns, the model robustly identifies the connection between lattice constants and spot positions to classify the seven crystal systems. Even when noise prevents accurate identification in the top prediction, the model often correctly recognizes this relationship in its second or third prediction.

For the space group classification for zone axis [100] [Table 6 ▸(*a*)], the impact of atomic substitutions is even more challenging for the model. At 0% substitution, the weighted accuracy is 74.72%, but it falls to 17.40% at 1% substitution and drops to 9.74% at 50% substitution, showing a sharp decline in model performance. The top-3 accuracy for space groups also drops, going from 88.25% at 0% to 18.35% at 50%. The same trend is evident in Table 6 ▸(*b*) for the accuracy classification for zone axis [111]. The perturbations caused by noise and intensity variations in 2D XRD patterns significantly impair the model’s ability to detect symmetry in spot patterns, posing substantial challenges for accurate classification of the 230 space groups. To address this, incorporating 2D XRD patterns with random atomic substitutions into the training dataset can enhance the model’s robustness. By training on such perturbed patterns, the model can better learn to recognize symmetry-related features, thereby improving its performance in classifying the 230 space groups, even under high levels of noise and perturbations.

### 3.6. Classification performance of defective structures

In real-world materials, experimental XRD patterns often exhibit noise and imperfections arising from intrinsic defects, impurities or measurement limitations (Lee *et al.*, 2023 ▸). To explore this, we conducted a new experiment to investigate the influence of systematically depleting atoms from the unit cell. To do this we created two phase transition processes: a non-body-centered cubic (non-b.c.c.) structure transforming to a b.c.c. and a non-face-centered cubic (non-f.c.c.) structure transforming to f.c.c.. We then analyzed simulated 2D XRD patterns step by step for these transitions.

In this section, the terms ‘f.c.c.’ and ‘b.c.c.’ specifically denote the fully ordered *F*-cubic and *I*-cubic Bravais lattices that each transition ultimately reaches, rather than generic centering labels applicable to lower-symmetry systems. The lattice constants were kept equal (*a* = *b* = *c*; α = β = γ = 90°) to maintain a cubic metric, but as atoms are progressively removed, some cubic symmetry operations are lost and the intermediate structures may temporarily display lower symmetry before the full cubic order is restored at complete depletion.

We randomly selected CIFs from non-b.c.c. and non-f.c.c. structures based on the 14 Bravais lattices from the training dataset. The initial structures were chosen to include the characteristic fractional atomic positions of the target sublattice: (0, 0, 0) and (½, ½, ½) for b.c.c., and (0, 0, 0), (½, ½, 0), (½, 0, ½) and (0, ½, ½) for f.c.c. These coordinates were held fixed throughout the depletion process so that, once all other atoms were removed, the structures naturally converged to the ideal b.c.c. or f.c.c. lattices. To keep the procedure consistent, each non-f.c.c. starting structure contained at least eight atoms and each non-b.c.c. structure contained at least six atoms prior to depletion. Crucially, the dataset includes a broad range of unit-cell sizes, including chemically complex structures with over 100 atoms [as detailed in Figs. N to U, and in Tables E to L (supporting information)], ensuring that atomic depletion functions as a random vacancy test rather than creating artificial long-range correlations typical of small-cell repetitions.

No structural relaxation was performed following atomic depletion. This choice is intentional and arises from the scientific purpose of the present work. Our study is not a physics-based simulation of defect energetics or structural response, but a controlled synthetic test designed to probe whether a neural network trained on ideal reciprocal-space projections can recognize symmetry-breaking perturbations introduced in a simplified manner. Introducing relaxation would conflate the direct effect of atomic removal with secondary structural adjustments governed by the energetics of the specific material system, thereby obscuring the controlled symmetry perturbation that we intend to study. By retaining fixed atomic coordinates, we establish a controlled benchmark for representational capacity, consistent with prior deep learning studies on crystal defects (Ziletti *et al.*, 2018 ▸).

While keeping these fractional coordinates fixed in the selected CIFs, we systematically depleted atoms from the unit cells at incremental steps of 0%, 25%, 50%, 75% and 100%. Through this process, we ensured that at 100%, the resulting structures corresponded precisely to b.c.c. or f.c.c. lattices. Thus, we could effectively track the phase transition from non-b.c.c. to b.c.c. and from non-f.c.c. to f.c.c. structures. This controlled depletion pathway provides a well defined progression towards fully ordered cubic structures, allowing us to observe how the diffraction symmetry changes at each stage of atomic removal.

**Figure 12:**
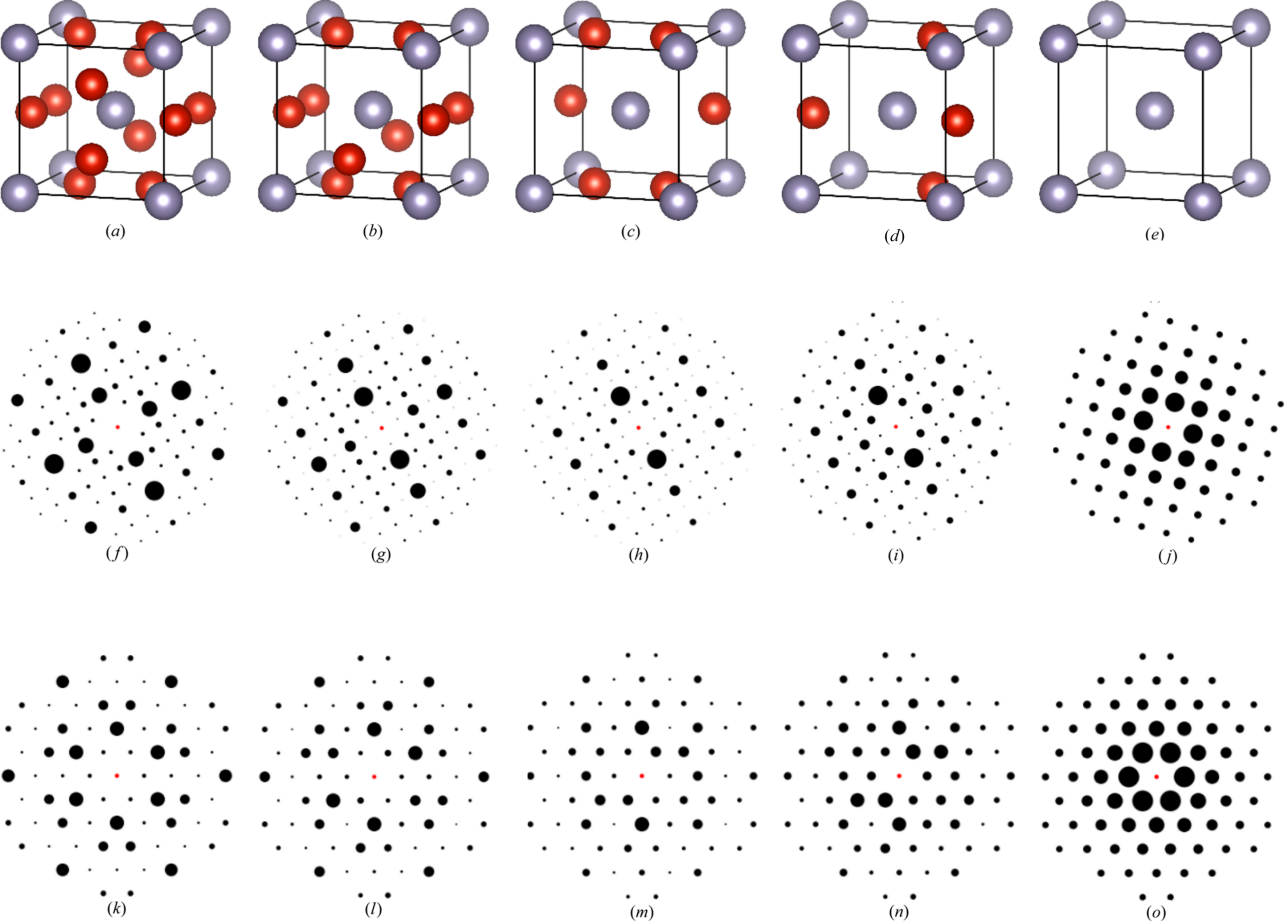
(*a*–*e*) The unit cell of the structure ICSD_ 106097 after applying defects. (*f*–*j*) 2D XRD patterns along the [100] zone axis under different defect percentages. (*k*–*o*) 2D XRD patterns along the [111] zone axis under different defect percentages. (*a*), (*f*), (*k*) 0% depletion; (*b*), (*g*), (*l*) 25% depletion; (*c*), (*h*), (*m*) 50% depletion; (*d*), (*i*), (*n*) 75% depletion; (*e*), (*j*), (*o*) 100% depletion. See Table 7 ▸ for further details.

**Figure 13:**
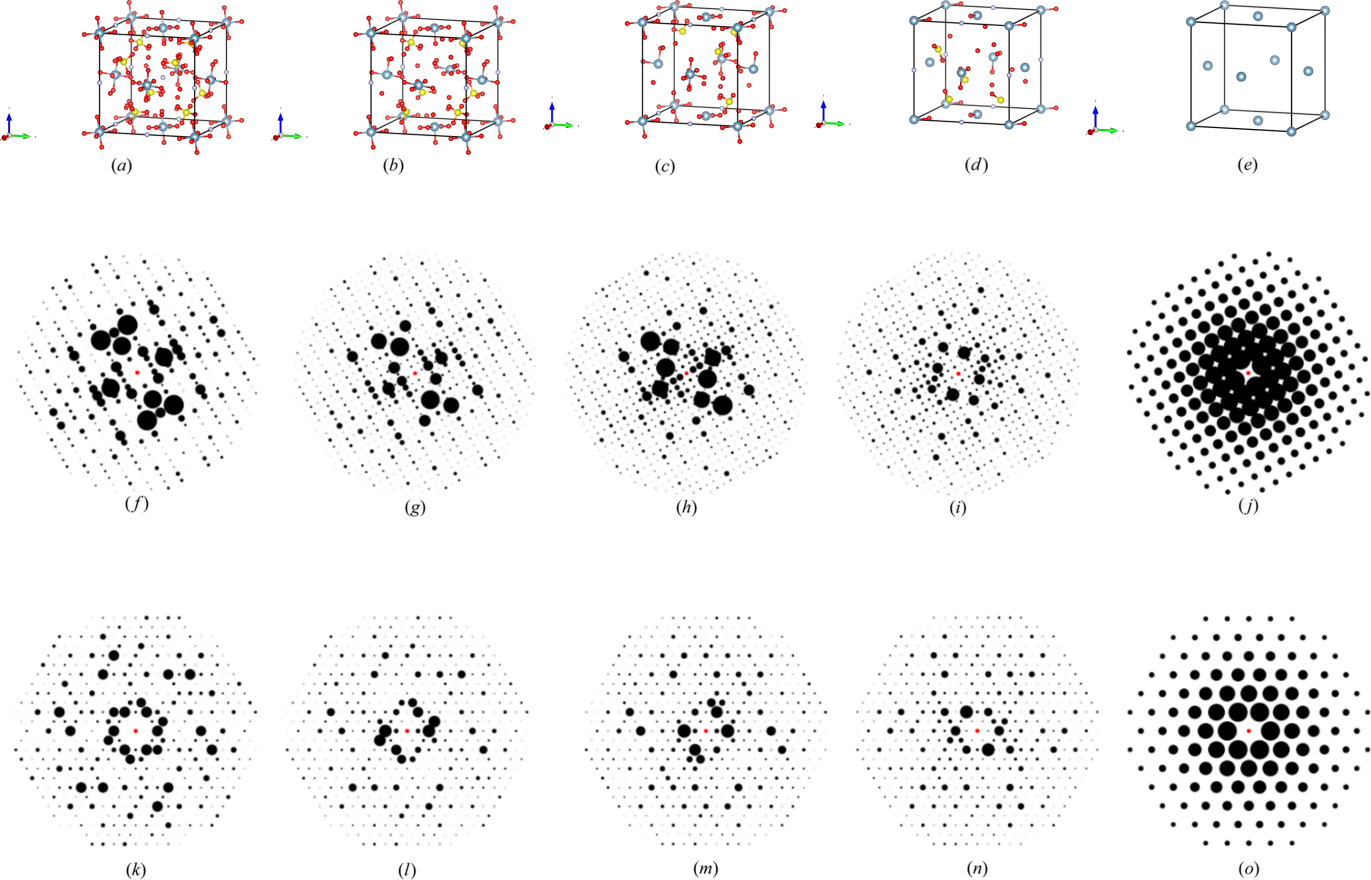
(*a*–*e*) The unit cell of the structure ICSD_ 014396 after applying defects. (*f*–*j*) 2D XRD patterns along the [100] zone axis under different defect percentages. (*k*–*o*) 2D XRD patterns along the [111] zone axis under different defect percentages. (*a*), (*f*), (*k*) 0% depletion; (*b*), (*g*), (*l*) 25% depletion; (*c*), (*h*), (*m*) 50% depletion; (*d*), (*i*), (*n*) 75% depletion; (*e*), (*j*), (*o*) 100% depletion. See Table 8 ▸ for further details.

Finally, we employed the ADP to generate synthetic 2D XRD patterns for each atomic depletion level (0%, 25%, 50%, 75% and 100%) under two distinct zone axes, [100] and [111]. We randomly selected five CIFs for the phase transition of non-b.c.c. to b.c.c. and five CIFs for non-f.c.c. to f.c.c., ensuring model-4 accurately classified the correct space groups at both 0% and 100% depletion. Additionally, model-4 classified the intermediate depletion levels (25%, 50% and 75%) throughout the phase transition. Figs. 12 ▸(*a*)–12 ▸(*e*) and Figs. 13 ▸(*a*)–13 ▸(*e*) illustrate structural changes within the unit cells at each atomic depletion step, while Figs. 12 ▸(*f*)–12 ▸(*o*) and Figs. 13 ▸(*f*)–13 ▸(*o*) present the corresponding synthetic 2D XRD patterns for the [100] and [111] zone axes at each depletion level.

Next, we employed *Phonopy*, an open-source Python package primarily designed for phonon calculations in materials science (Togo & Tanaka, 2015 ▸). Besides its phonon-related functionalities, *Phonopy* accurately analyzes crystal structure symmetry and determines the corresponding space group on the basis of complete 3D atomic structural information, including atomic positions, lattice parameters and symmetry operations (Togo & Tanaka, 2015 ▸). To utilize *Phonopy*, we first generated POSCAR (VASP format) files for structures at depletion levels of 0%, 25%, 50%, 75% and 100%. *Phonopy*’s space group prediction is derived directly from fully detailed 3D atomic structures, without dependence on zone axis orientation, making its predictions highly accurate. Model-4, which in contrast classifies space groups solely on the basis of 2D XRD patterns, achieved classification performance remarkably comparable to that of *Phonopy*.

In Tables 7 ▸ and 8 ▸, we compare the space group predictions obtained from *Phonopy* (using POSCAR files) with those from our trained model-4 (using synthetic 2D XRD patterns). Both *Phonopy* and model-4 correctly identify the space group with 0% deviation from the space group recorded in the CIFs. For analyzing the 100% depletion level in Tables 7 ▸ and 8 ▸, it is essential to first establish that, based on the 14 Bravais lattices, space group Nos. 197, 199, 204, 206, 211, 214, 217, 220, 229 and 230 correspond to b.c.c. (*I*), while space group Nos. 196, 202, 203, 209, 210, 216, 219, 225, 226, 227 and 228 correspond to f.c.c. (*F*) (He, 2018 ▸). Using this knowledge, we observe in Tables 7 ▸ and 8 ▸ that both *Phonopy* and model-4 correctly predicted b.c.c. and f.c.c. structures in the 100% depletion level.

However, at atomic depletion levels of 25%, 50% and 75% under both zone axes [100] and [111], model-4 predicted space groups outside the cubic range (space groups 195–230), indicating that the progressive removal of atoms disrupts symmetry operations. Similarly, *Phonopy* showed non-cubic predictions at these atomic depletion levels. These results show that even with a fixed cubic lattice the gradual removal of atoms can locally disrupt cubic symmetry, lowering the effective space group symmetry until full atomic order is recovered at complete depletion. This agreement between a diffraction-based classifier and a structure-based solver is precisely the behavior we intended to test.

For completeness, additional structures and related tables are provided in Figs. N to U, and in Tables E to L (supporting information).

From a conceptual viewpoint, this behavior can be interpreted through the Bragg–Williams long-range-ordering framework. In this context, atomic depletion acts as an order parameter η that quantifies the occupancy of the endpoint sublattice. As η increases, superlattice periodicities emerge, altering reflection conditions and momentarily reducing apparent symmetry before the system reaches the fully ordered *F*-cubic (f.c.c.) or *I*-cubic (b.c.c.) states. This analogy provides a clear physical interpretation of the observed symmetry evolution.

This experiment does not model any specific chemical ordering process, such as a Heusler-to-disordered-f.c.c. transition. Instead, it systematically varies atomic occupancy while keeping the key sublattice positions of the target f.c.c. or b.c.c. structures fixed, allowing us to examine how the diffraction-visible symmetry changes at each stage. Since an ideal f.c.c. or b.c.c. lattice yields identical reflection conditions for both the unit cell and its repeated supercell, analyzing the unit cell alone is sufficient for this study.

## 4. Code availability

The source code for the Auto Diffraction Pipeline, developed for this study to generate synthetic 2D XRD patterns, and the source code for the deep learning models, including the CNN architectures used for crystallographic classification tasks, are publicly available on GitHub at https://github.com/niaz60/DiffAI.

## 5. Discussion and conclusion

In this study, we demonstrated the use of synthetic 2D XRD patterns, derived from CIFs, as the primary input for training deep learning models on crystallographic classification tasks. On conversion of real-space lattice information from CIFs into reciprocal space, each crystallographic orientation (zone axis) produces distinctive spot patterns that capture the symmetry and atomic arrangement of a structure. These 2D XRD patterns serve as compact, information-rich representations of crystallographic data, enabling the deep learning models to learn spatial features such as spot positions, intensities and symmetry relationships.

A key component of this work is the development of the ADP. This Python-based framework automates the generation of synthetic 2D XRD patterns along predefined or user-selected zone axes. By offering fine control over lattice parameters, atomic positions and symmetry operations, the ADP facilitates data generation under diverse conditions, including atomic substitution, atomic depletion and mechanical loading. Critically, it can produce large and varied datasets without relying on time-consuming or costly experimental measurements. This capability enables the creation of balanced datasets encompassing thousands of structures and multiple crystallographic orientations, an essential factor in training robust machine learning models.

To effectively learn from these synthetic 2D XRD patterns, we employed a CNN architecture designed around the nature of the input data. The network’s convolution blocks extract key local features from the 2D spot patterns, while subsequent fully connected layers handle higher-level feature integration. Separate classification heads were used for the seven crystal systems and the 230 individual space groups, reflecting the different degrees of complexity in each classification task. The layered structure of the CNN, combined with max pooling and carefully tuned hyperparameters, helped the model capture subtle rotational and intensity differences among the 2D XRD patterns.

Nonetheless, real-world 2D XRD experiments introduce challenges that can limit the utility of purely manual analysis. Different zone axes, slight misalignments in sample orientation and diverse instrument settings can lead to discrepancies in the measured diffraction patterns. Furthermore, collecting high-quality, experimentally obtained 2D XRD data remains costly and time consuming, contributing to the scarcity of comprehensive datasets. Such limitations can impede researchers’ ability to develop and train robust models on real-world samples. The approach described here addresses these challenges by synthesizing 2D XRD patterns for a wide range of orientations and structural scenarios. This automated, data-driven framework reduces reliance on costly experimental campaigns, since patterns can be generated under different conditions. Consequently, the models gain exposure to a broad spectrum of diffraction signatures, improving their adaptability to real-world scenarios where orientation or experimental conditions may vary.

Throughout this study, the deep learning models demonstrated robust performance across a wide range of classification tasks. Initially, the training process focused on a single zone axis, but the training scope was progressively expanded to include four, ten and 20 zone axes. This incremental approach allowed the models to learn from increasingly diverse crystallographic orientations. The trained models were then rigorously tested on multiple scenarios, including previously seen and unseen zone axes, isotropic lattice scaling conditions, atomic substitution, and defect structures. Despite the inherent complexity of classifying 230 space groups, the models consistently achieved high accuracy, with even stronger performance observed when distinguishing among the seven crystal systems. Overall, these results highlight the robustness of our approach and suggest that the models are well equipped to handle a variety of practical crystallographic classification challenges. Although this study utilizes synthetic data, the model’s demonstrated resilience to significant intensity variations (atomic substitutions) and geometric perturbations (lattice scaling) indicates that the learned representations are robust to distribution shifts, satisfying a critical prerequisite for future application to experimental data.

To maintain computational efficiency, we limited our training set to integer zone axes. However, the model’s ability to accurately classify unseen axes confirms that it generalizes well to orientations that lie between these integers. This robustness is essential for experimental applications, where sample orientation varies continuously and cannot always be aligned to specific integer axes.

Furthermore, this automated 2D deep learning framework offers distinct advantages for high-pressure research, particularly in the analysis of textured polycrystals common in diamond anvil cell (DAC) experiments. While such data are frequently reduced to 1D profiles via azimuthal integration, this process averages signal intensity along diffraction rings, effectively erasing the directional intensity variations caused by preferred orientation. By analyzing the full 2D pattern, the CNN can utilize these textural nuances for classification. In our previous work, we demonstrated that 1D-based deep learning models achieve high accuracy for phase identification in standard untextured polycrystals or single crystals. However, given the unique challenges of preferred orientation, benchmarking the performance of 1D versus 2D models specifically on textured materials would serve as a valuable objective for future community research. Our model’s demonstrated robustness to isotropic lattice compression (Section 3.4) and symmetry-breaking atomic depletion (Section 3.6) further indicates its potential for tracking phase transitions and identifying structures under the extreme volumetric constraints typical of high-pressure environments.

In addition, geometric ambiguities can arise when different space groups produce nearly indistinguishable diffraction patterns along specific zone axes, particularly enantiomorphic or centrosymmetric counterparts, as illustrated in Fig. V (1–4) (supporting information). Because a single 2D projection provides only a partial view of reciprocal space, complete symmetry determination is under-constrained. To make this limitation explicit, we report both top-1 and top-3 accuracies, showing that the true space group commonly appears among the three highest-ranked predictions. Future work will extend the ADP to incorporate experimental-realism modules together with variable-resolution inputs, multi-axis fusion and 3D reciprocal-space reconstruction, thereby enhancing robustness and generalization to real measurements.

While the ADP provides a flexible and scalable data-generation framework, the present benchmark deliberately employs an idealized configuration to isolate the intrinsic representational capacity of 2D diffraction patterns. All simu­lated images are generated from CIF-derived structure factors with no added noise, background or detector imperfections, and are rendered at a fixed resolution of 321 × 322 pixels (100 d.p.i.). This controlled setup enables systematic evaluation of model capability but limits direct transferability to experimental data collected under varied detector geometries or noise conditions. Importantly, this simplification represents a modeling choice rather than an intrinsic limitation of the ADP framework. In our previous work on 1D powder XRD, we incorporated experimental realism through peak-shape variation, background modeling, Caglioti-type broadening and controlled noise injection to train CNNs robust to real measurements (Salgado *et al.*, 2023 ▸), providing the foundation for future adaptation of these realism modules to 2D diffraction data. To systematically bridge the domain shift between synthetic and real data, future implementation will employ a staged strategy: first generating datasets en­riched with these realistic artifacts, and subsequently utilizing transfer learning or mixed synthetic–experimental training on well characterized single-crystal standards. This hybrid training approach is grounded in our prior work on 1D XRD, where mixing experimental data with synthetic patterns was demonstrated to significantly improve model generalization to unseen experimental domains (Salgado *et al.*, 2023 ▸).

Despite these advancements, obtaining large, unified datasets for training comprehensive models is not always feasible, particularly in distributed development scenarios. In such cases, developers often train models independently using private datasets, which may vary significantly across domains and materials. This leads to models with divergent learned knowledge, potentially limiting their generalizability. Further research is needed to explore methods for integrating these independently trained models without requiring access to private data. Such approaches should aim to enhance performance on out-of-distribution data and improve overall model robustness, ensuring broader applicability in crystallographic analysis.

## Supplementary Material

Supporting information. DOI: 10.1107/S1600576726000099/yr5160sup1.pdf

## Figures and Tables

**Table 1:** Overview of the zone axes selected at various stages (one, four, ten and 20) to generate synthetic 2D XRD patterns for training and testing the space group and crystal system classification models, along with the corresponding number of simulated 2D XRD patterns for each model

Models	No. of zone axes	Selected zone axes	No. of simulated 2D XRD patterns
Model-1	1 zone axis	[100]	52191
Model-2	4 zone axes	[100], [010], [001], [111]	208764
Model-3	10 zone axes	[100], [010], [001], [111], [110], [101], [210], [012], [112], [121]	521910
Model-4	20 zone axes	[100], [010], [001], [111], [110], [101], [210], [012], [112], [121], [122], [212], [103], [310], [123], [312], [023], [302], [313], [331]	1043820

**Table 2:** Weighted and top-3 accuracies of model-1 trained on the zone axis [100] and tested on zone axes [111], [100], [010] and [001]

Classification	Performance metrics	Testing zone axes			
		[100]	[010]	[001]	[111]
7 crystal systems	Weighted accuracy	83.61%	71.93%	44.41%	19.04%
	Top-3 accuracy	98.49%	96.53%	82.84%	53.99%
230 space groups	Weighted accuracy	63.95%	35.62%	14.79%	1.73%
	Top-3 accuracy	76.11%	50.97%	21.13%	4.63%

**Table:** (*a*) Classifying seven crystal systems.

Models	Training zone axes	Category	Testing zone axes		
			4 zone axes	10 zone axes	20 zone axes
Model-2	4	Weighted accuracy	86.94%	54.88%	40.51%
		Top-3 accuracy	99.15%	80.17%	66.67%
Model-3	10	Weighted accuracy	85.39%	85.37%	59.61%
		Top-3 accuracy	99.07%	98.92%	84.52%
Model-4	20	Weighted accuracy	83.33%	83.46%	82.71%
		Top-3 accuracy	98.81%	98.70%	98.34%

**Table:** (*b*) Classifying 230 space groups

Models	Training zone axes	Category	Testing zone axes		
			4 zone axes	10 zone axes	20 zone axes
Model-2	4	Weighted accuracy	66.32%	30.91%	16.61%
		Top-3 accuracy	81.00%	40.45%	23.08%
Model-3	10	Weighted accuracy	61.05%	62.99%	35.03%
		Top-3 accuracy	78.42%	80.09%	47.80%
Model-4	20	Weighted accuracy	58.08%	60.81%	60.59%
		Top-3 accuracy	76.05%	78.39%	78.36%

**Table 4:** Weighted accuracy and top-3 accuracy achieved by model-4, showcasing classification performance for seven crystal systems and 230 space groups, including results for unseen zone axes [211], [011], [120] and [131]

Classification	Accuracy	Unseen zone axes			
		[131]	[211]	[011]	[120]
7 crystal systems	Weighted accuracy	41.55%	60.70%	62.14%	65.11%
	Top-3 accuracy	64.08%	81.41%	82.02%	84.03%
230 space groups	Weighted accuracy	15.78%	37.60%	38.10%	38.72%
	Top-3 accuracy	31.20%	54.57%	57.68%	57.42%

**Table 5:** Model accuracy for predicting seven crystal systems and 230 space groups under isotropic lattice scaling conditions Results are presented for tensile strains (+5%, +10%) and compressive strains (−5%, −10%) applied to cubic unit cells from the training dataset.

Zone axes	Classification	Deformation (compression and expansion %)				
		−10%	−5%	0	+5%	+10%
[100]	7 crystal systems	94.35%	97.40%	98.97%	97.33%	95.36%
	230 space groups	76.60%	89.50%	94.25%	89.78%	81.02%
[111]	7 crystal systems	96.85%	98.08%	99.19%	98.53%	98.23%
	230 space groups	66.76%	80.16%	86.47%	81.20%	70.14%

**Table:** (*a*) Atomic substitutions under the [100] zone axis.

Zone axis	Classification	Accuracy	Atomic substitutions %						
			0%	1%	2%	5%	10%	25%	50%
[100]	7 crystal systems	Weighted accuracy	87.66%	62.55%	62.33%	60.94%	60.49%	58.79%	57.32%
		Top-3 accuracy	99.64%	93.91%	93.89%	93.28%	92.45%	92.28%	91.53%
	230 space groups	Weighted accuracy	74.72%	17.40%	16.11%	14.48%	13.28%	11.31%	9.74%
		Top-3 accuracy	88.25%	28.90%	27.34%	26.04%	23.93%	20.68%	18.35%

**Table:** (*b*) Atomic substitutions under the [111] zone axis.

Zone axis	Classification	Accuracy	Atomic substitutions %						
			0%	1%	2%	5%	10%	25%	50%
[111]	7 crystal systems	Weighted accuracy	85.11%	65.69%	64.58%	62.87%	61.68%	59.83%	58.67%
		Top-3 accuracy	99.21%	95.32%	95.16%	94.89%	94.69%	94.59%	94.65%
	230 space groups	Weighted accuracy	71.61%	20.65%	18.49%	16.19%	13.70%	11.15%	9.17%
		Top-3 accuracy	87.77%	29.83%	28.03%	24.87%	22.22%	18.51%	15.67%

**Table 7:** The space group No. predictions of *Phonopy* and model-4 under different defect percentages in the sample structure ICSD_106097

Depletion	0% (non-b.c.c.)	25%	50%	75%	100% (b.c.c.)
Chemical component	V_6_Sn_2_	V_5_Sn_2_	V_3_Sn_2_	V_2_Sn_2_	Sn_2_
*Phonopy* classification	223	115	25	40	229
Model-4’s prediction – zone [100]	223	139	107	59	229
Model-4’s prediction – zone [111]	223	223	5	5	229

**Table 8:** The space group No. predictions of *Phonopy* and model-4 under different defect percentages in the sample structure ICSD_014396

Depletion	0% (non-f.c.c.)	25%	50%	75%	100% (f.c.c.)
Chemical component	Al_4_N_4_O_80_S_8_	Al_4_N_1_O_62_S_6_	Al_4_N_2_O_40_S_4_	Al_4_N_2_O_17_S_4_	Al_4_
*Phonopy* classification	205	1	1	1	225
Model-4’s predicttion – zone [100]	205	1	33	1	226
Model-4’s prediction – zone [111]	205	4	4	4	196

## Data Availability

The full crystallographic dataset is subject to licensing restrictions and is not publicly available. However, it can be accessed upon request from the Inorganic Crystal Structure Database (ICSD) at https://icsd.products.fiz-karlsruhe.de. The datasets of 2D XRD patterns generated in this study have been deposited in Zenodo and are available at https://doi.org/10.5281/zenodo.15455282. Additional data supporting the findings of this article are available from the corresponding author upon reasonable request.
