# Supplementary material for: Machine learning approaches for crystallographic classification from synthetic 2D X-ray diffraction data
Source: J Appl Crystallogr. 2026 Feb 1;59(Pt 1):206–24. doi: 10.1107/S1600576726000099 (PMC12871478; doi:10.1107/S1600576726000099)
Supplement: Supplementary file 1 [file j-59-00206-sup1.pdf]

JOURNAL OF APPLIED CRYSTALLOGRAPHY

Volume 59 (2026)

Supporting information for article:

Machine learning approaches for crystallographic classification from synthetic 2D X-ray diffraction data

Ayoub Shahnazari, Zeliang Zhang, Sachith E. Dissanayake, Chenliang Xu and Niaz Abdolrahim

Supplementary

Supplementary Figure A illustrates the distribution of 177,432 CIFs sourced from the Inorganic Crystal Structure Database (ICSD) (Belsky, Hellenbrandt et al. 2002) across 230 space groups, categorized into seven crystal systems: Cubic, Hexagonal, Trigonal, Tetragonal, Orthorhombic, Monoclinic, and Triclinic. The dataset was curated by removing incomplete or duplicated structures to ensure data integrity. Each space group is represented, with colors distinguishing the seven crystal systems. A legend accompanies the figure, quantifying the number of CIFs associated with each crystal system, providing a clear visual representation of the dataset's structural diversity and symmetry distribution.

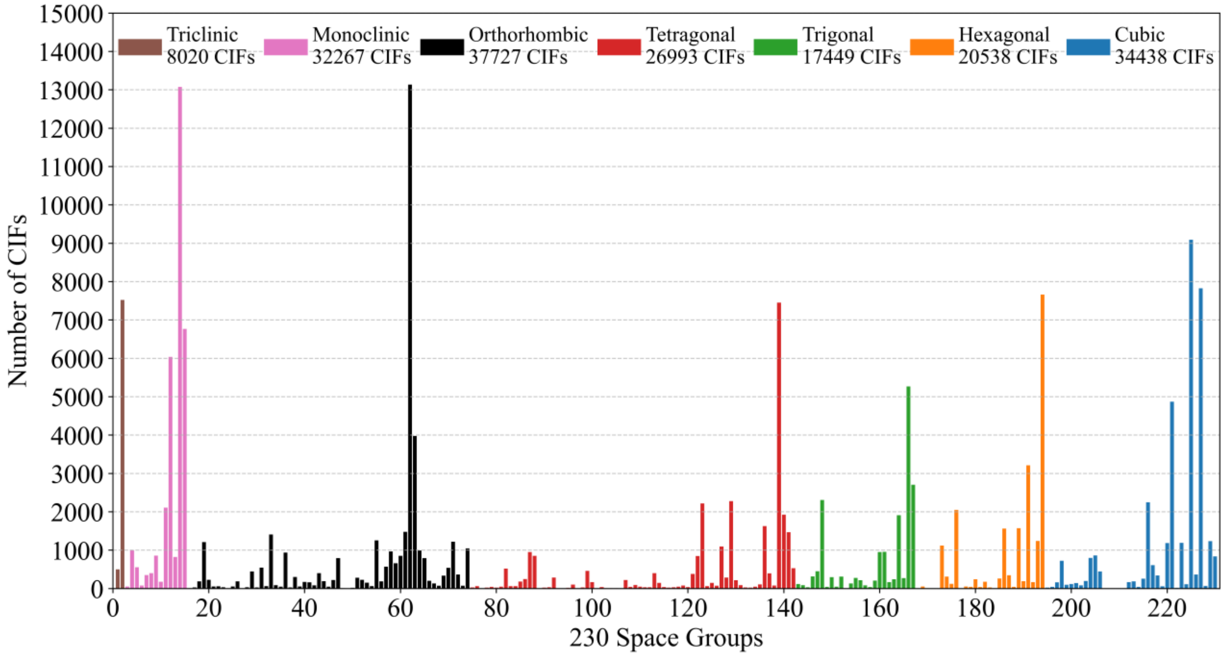

Supplementary Figure A depicts the initial distribution of 177,432 CIFs across 230 space groups from the ICSD dataset. The figure uses colors to represent the seven crystal systems (Cubic, Hexagonal, Trigonal, Tetragonal, Orthorhombic, Monoclinic, and Triclinic), and includes a legend indicating the number of CIFs corresponding to each crystal system.

To validate the geometric coverage of our approach, we performed a quantitative analysis of the distribution of the 20 selected zone axes used in Model-4. This analysis revealed an average nearest-neighbor angular separation of 13.03 degree, alongside a global average separation of 44.32 degree across all unique pairs. These metrics confirm that our strategic selection of low-index axes achieves a local sampling resolution comparable to that of a dense 15 degree grid, which would typically require approximately 216 distinct orientations.

This selection strategy represents a critical balance between sampling density and computational feasibility. While a uniform grid of 216 orientations would necessitate the generation and processing of over 11 million synthetic 2D XRD patterns, given our baseline of over 52k CIFs, our approach maintains the dataset at a manageable size of approximately 1 million patterns. This reduction allows for efficient training without sacrificing the capture of crystallographically significant features inherent to high-symmetry orientations. The pairwise angular relationships are visualized in Supplementary Figure B, which displays the heatmap matrix of angular separations matrix.

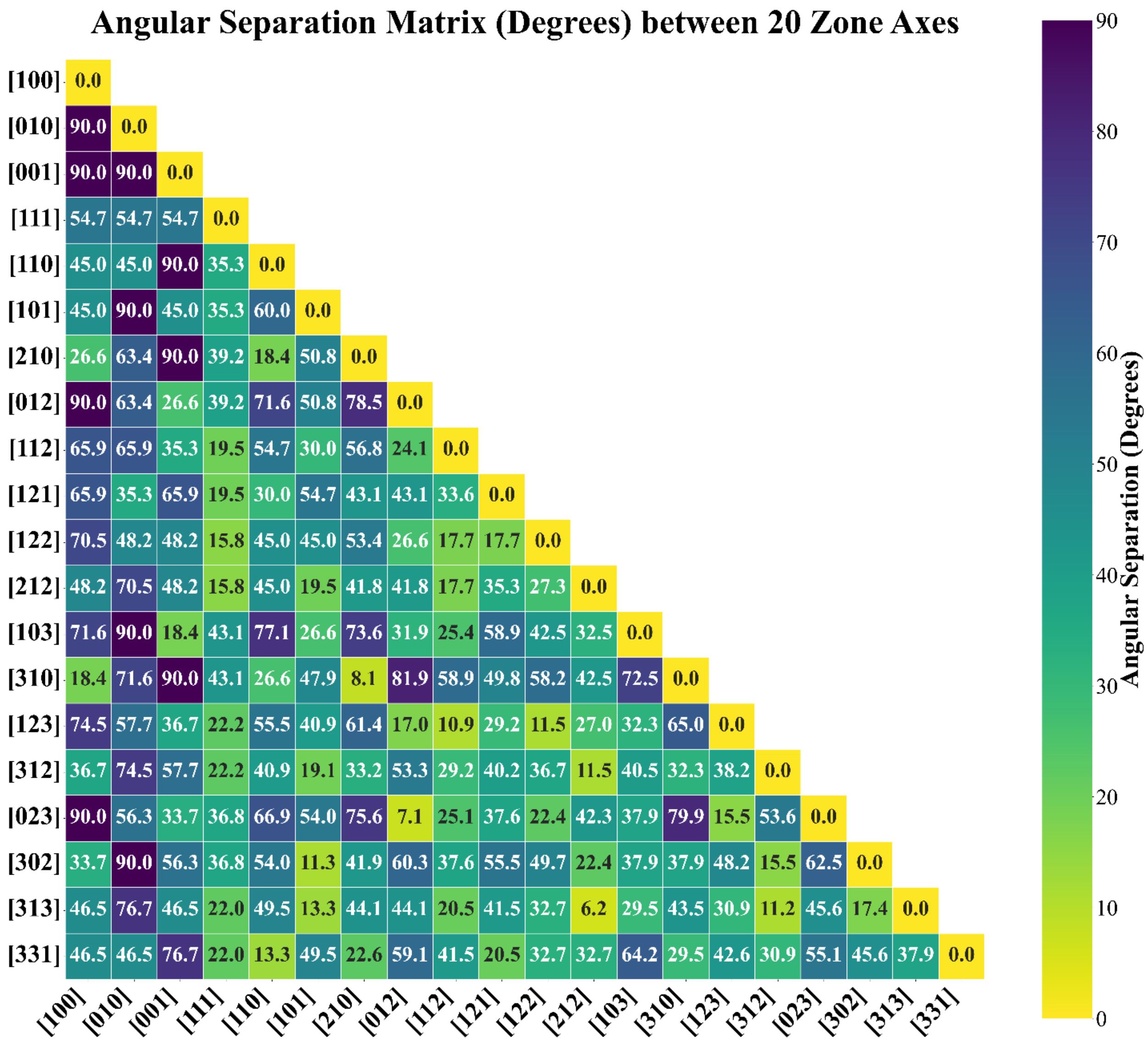

Supplementary Figure B Matrix of pairwise angular separations (in degrees) between the 20 crystallographic zone axes used for training Model-4. The color gradient indicates the magnitude of separation, with yellow representing small angles (high proximity) and dark purple representing large angles (orthogonality). The analysis reveals an average nearest-neighbor angular separation of 13.03°, confirming that the selected low-index axes achieve a local sampling resolution comparable to the 15° resolution threshold suggested for effective coverage.

Supplementary Figure C presents four confusion matrices illustrating Model-1's performance in classifying seven crystal systems (cubic, tetragonal, orthorhombic, monoclinic, triclinic, hexagonal, and trigonal) based on 2D XRD patterns. The model was trained on patterns from the [100] zone axis and tested across four zone axes: (1) [100], (2) [010], (3) [001], and (4) [111]. Each matrix displays accuracy percentages, with rows representing true crystal systems and columns indicating predicted ones. Diagonal cells show the percentage of correct classifications, while off-diagonal cells highlight misclassifications.

Supplementary Figure D complements Supplementary Figure C by presenting four confusion matrices for the same classification task, but instead of accuracy percentages, it reports the number of correct and incorrect predictions. Like Supplementary Figure C, the model was trained on [100] zone axis patterns and tested on the [100], [010], [001], and [111] zone axes, with rows and columns representing true and predicted crystal systems, respectively. Diagonal cells indicate the count of correct classifications, while off-diagonal cells show the count of misclassifications. Both figures reveal variations in classification performance, with reduced accuracy for the [010] and [001] zone axes and substantially lower accuracy for the [111] zone axis compared to [100], reflecting the influence of crystallographic symmetry on Model-1's effectiveness in crystal system classification.

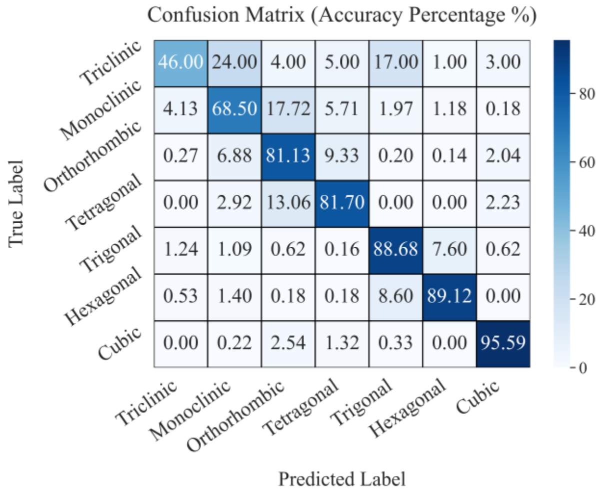

(1) Trained on one zone [100] and tested on [100] zone axis

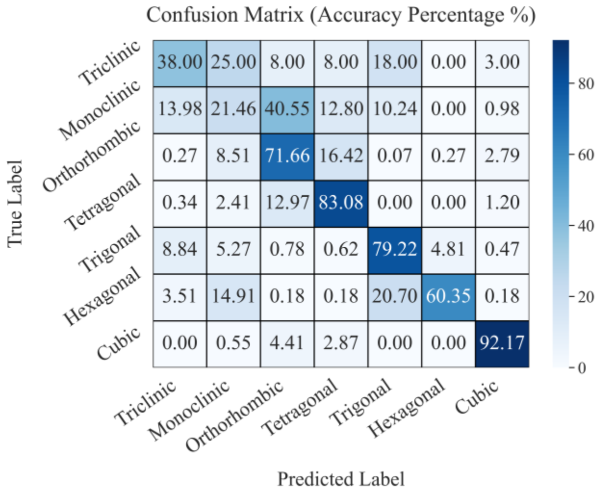

(2) Trained on one zone [100] and tested on [010] zone axis

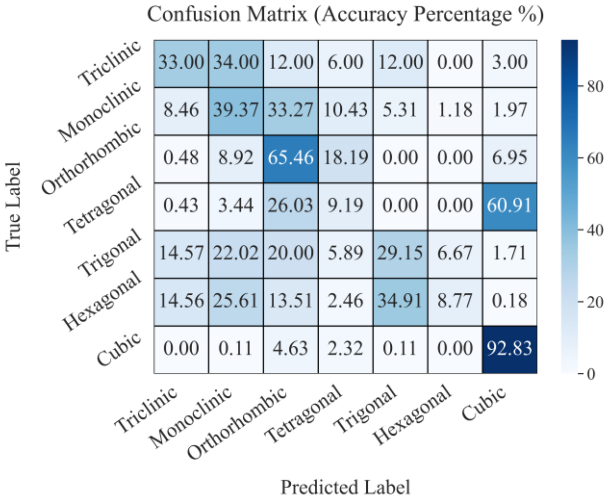

(3) Trained on one zone [100] and tested on [001] zone axis

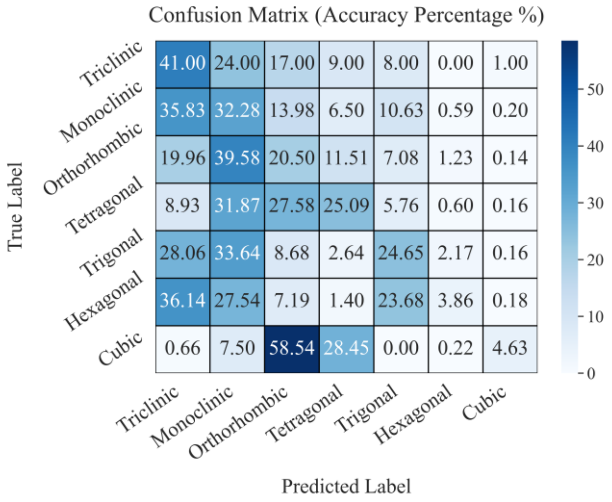

(4) Trained on one zone [100] and tested on [111] zone axis

Supplementary Figure C Confusion matrix based on accuracy percentage for classification 7 crystal systems, where the model was trained on zone axis [100] and tested on zone axes (1) [100], (2) [010], (3) [001], and (4) [111].

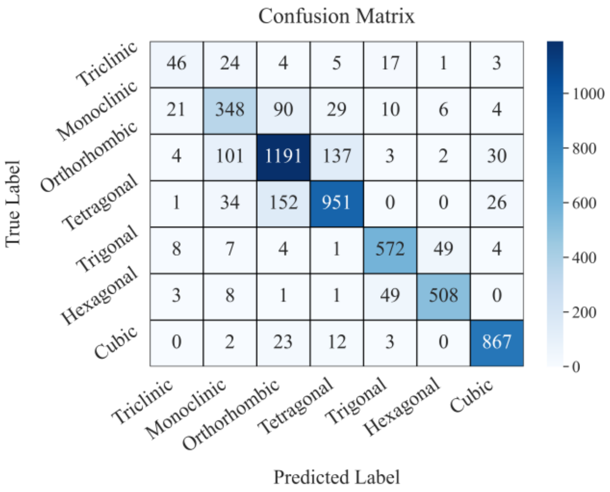

(1) Trained on one zone [100] and tested on [100] zone axis

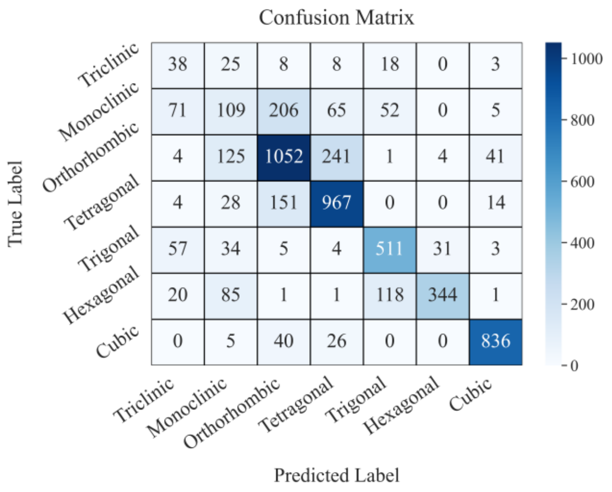

(2) Trained on one zone [100] and tested on [010] zone axis

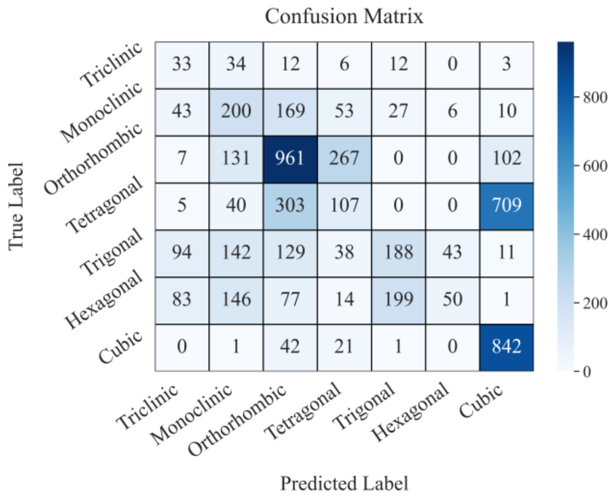

(3) Trained on one zone [100] and tested on [001] zone axis

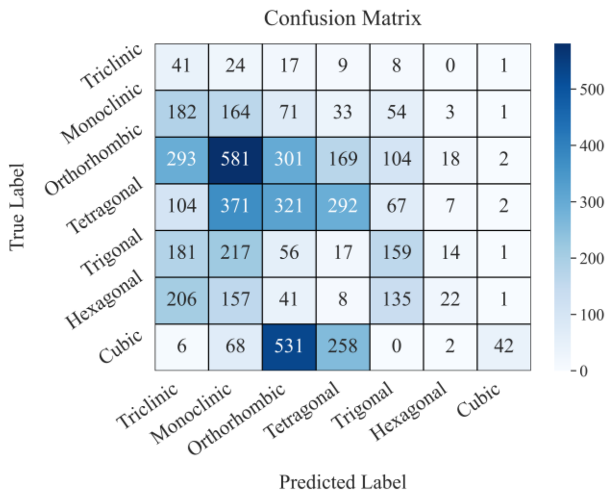

(4) Trained on one zone [100] and tested on [111] zone axis

Supplementary Figure D Confusion matrix based on the number of correct prediction for predicting 7 crystal systems, where the model was trained on zone axis [100] and tested on zone axes (1) [100], (2) [010], (3) [001], and (4) [111].

Supplementary Figure E illustrates the performance of Model-1 in classifying the 230 space groups, with accuracy reported for each space group. Unlike Supplementary Figure C and Supplementary Figure D, which utilize confusion matrices to depict classification performance for the seven crystal systems, this figure focuses on presenting the percentage of correct predictions across the 230 space groups without a full confusion matrix. The model was trained on diffraction patterns along the [100] zone axis and evaluated on patterns from multiple zone axes: (1) [100], (2) [010], (3) [001], and (4) [111]. This figure highlights the challenges of fine-grained classification among a large number of categories, as the task of predicting 230 space groups is inherently more complex than classifying the seven crystal systems.

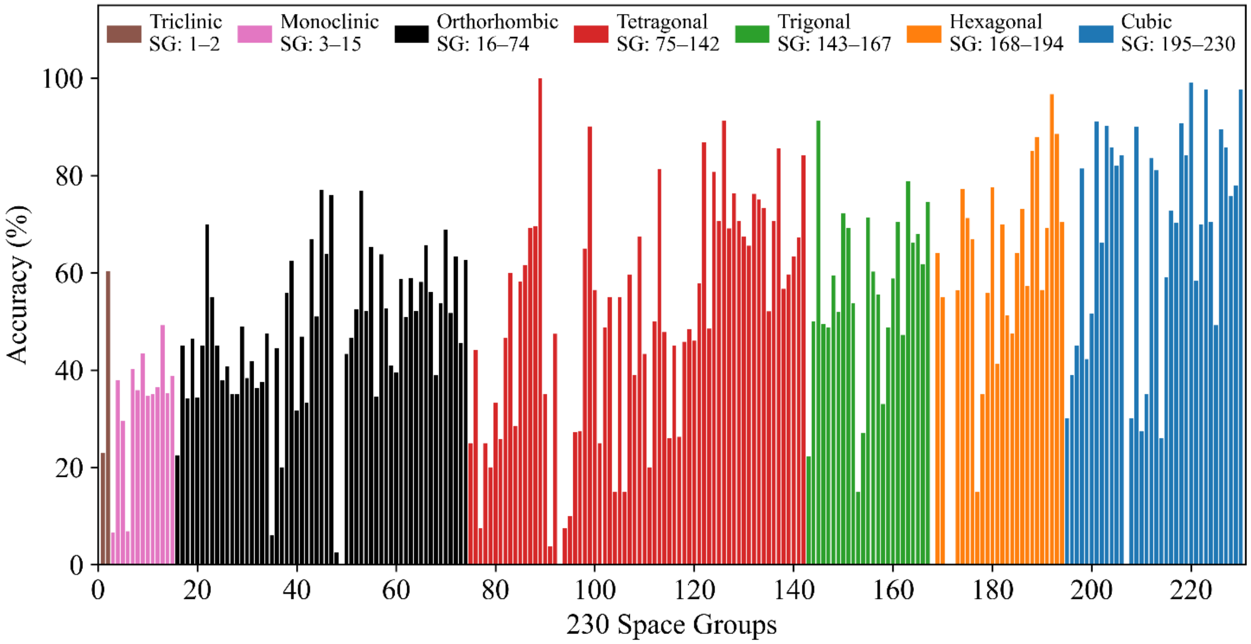

(1) Trained on one zone [100] and tested on [100] zone axis.

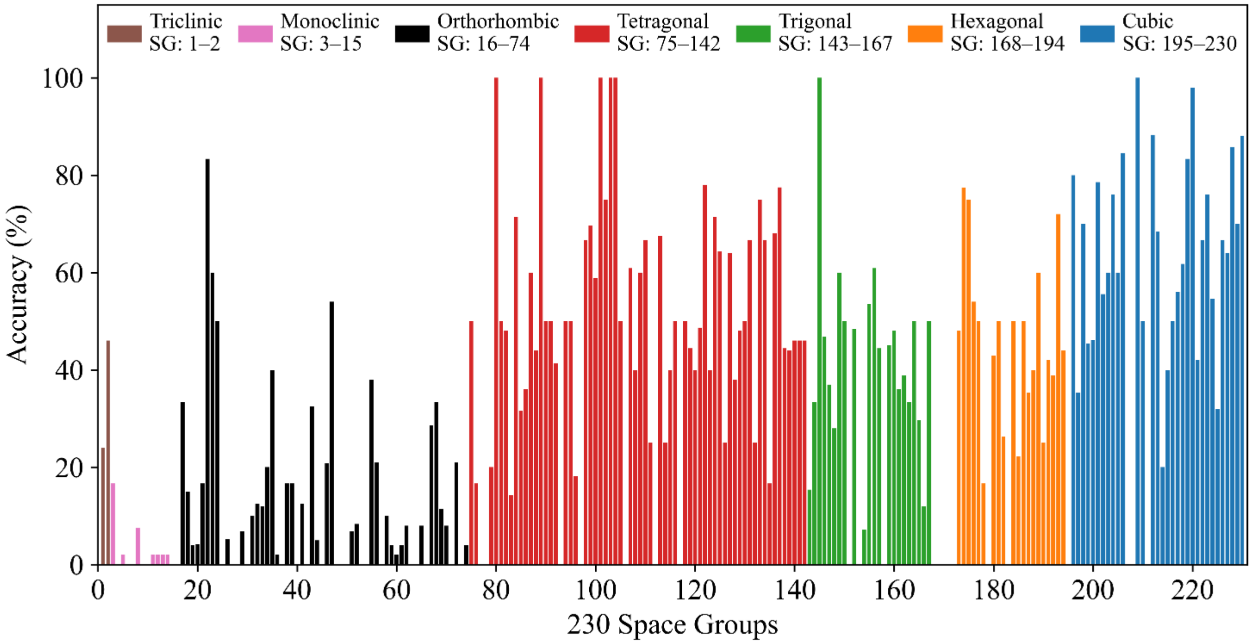

(2) Trained on one zone [100] and tested on [010] zone axis.

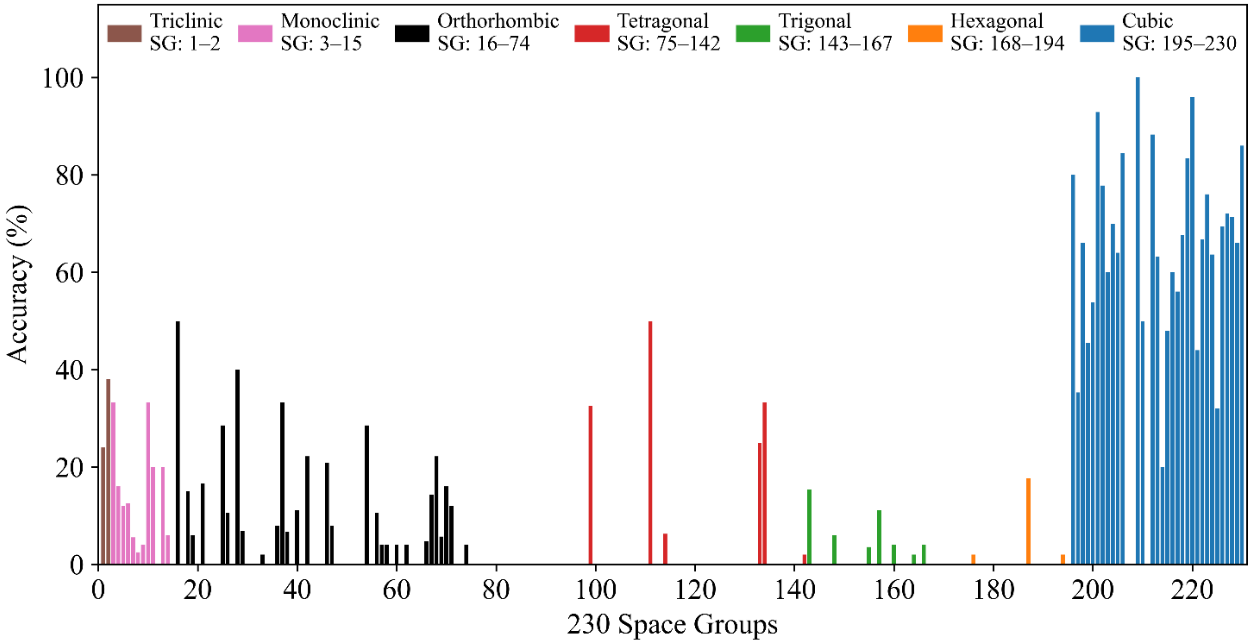

(3) Trained on one zone [100] and tested on [001] zone axis.

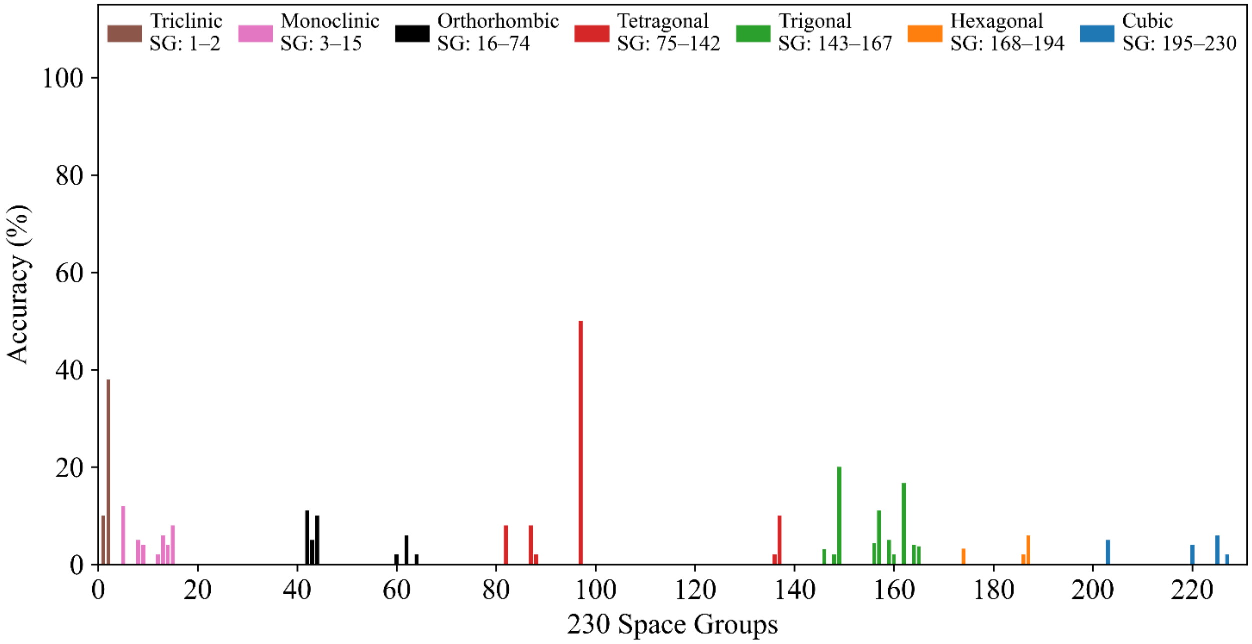

(4) Trained on one zone [100] and tested on [111] zone axis.

Supplementary Figure E The percentage of correct predictions vs. 230 space groups, where the model-1 was trained on zone axis [100] and tested (1) [100], (2) [010], (3) [001], and (4) [111]on zone axes

Supplementary Figure F shows confusion matrices for Model-4, assessing top-1 prediction accuracy across all 11 enantiomorphic space group pairs: (a) 76 vs. 78, (b) 91 vs. 95, (c) 92 vs. 96, (d) 144 vs. 145, (e) 151 vs. 153, (f) 152 vs. 154, (g) 169 vs. 170, (h) 171 vs. 172, (i) 178 vs. 179, (j) 180 vs. 181, and (k) 212 vs. 213. Each subfigure displays a confusion matrix assessing the model's ability to classify the two space

groups within each pair, based on 2D X-ray diffraction patterns collected from 20 zone axes. Enantiomorphic space groups, being chiral mirror images of each other, exhibit nearly identical diffraction patterns due to Friedel's Law, posing a significant challenge for accurate classification. The matrices quantify this difficulty, with diagonal elements representing the percentage of correct predictions and off-diagonal elements indicating misclassification rates between the paired space groups. These results highlight the model's tendency to confuse enantiomorphic counterparts.

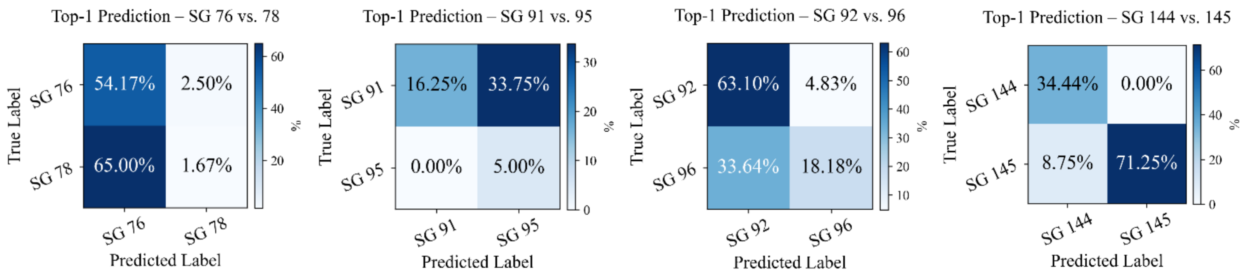

(a) Top-1 prediction, Space group 76_vs._78
(b) Top-1 prediction, Space group 91_vs._95
(c) Top-1 prediction, Space group 92_vs._96
(d) Top-1 prediction, Space group 144_vs._145

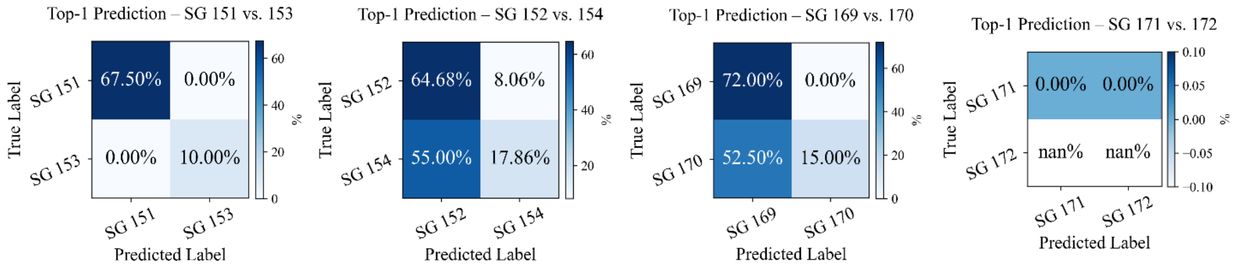

(e) Top-1 prediction, Space group 151_vs._153
(f) Top-1 prediction, Space group 152_vs._154
(g) Top-1 prediction, Space group 169_vs._170
(h) Top-1 prediction, Space group 171_vs._172.

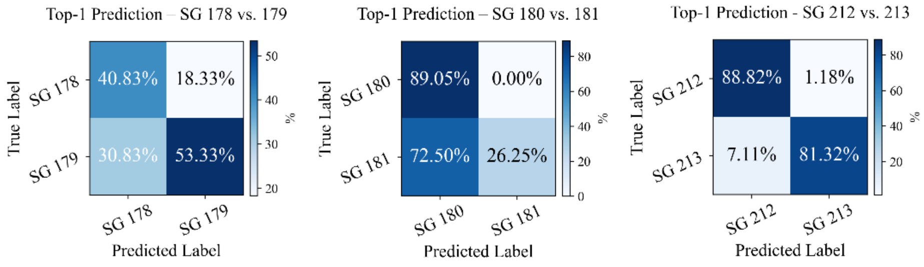

(i) Top-1 prediction, Space group 178_vs._179
(j) Top-1 prediction, Space group 180_vs._181
(k) Top-1 prediction, Space group 212 vs. 213

Supplementary Figure F Confusion matrix for Model-4, evaluating top-1 prediction performance across 11 enantiomorphic space group pairs: (a) 76 vs. 78, (b) 91 vs. 95, (c) 92 vs. 96, (d) 144 vs. 145, (e) 151 vs. 153, (f) 152 vs. 154, (g) 169 vs. 170, (h) 171 vs. 172, (i) 178 vs. 179, (j) 180 vs. 181, and (k) 212 vs. 213. The matrix highlights misclassification challenges attributed to chirality.

Supplementary Figure G presents confusion matrices for Model-4 evaluated on the 20 zone axis test dataset, assessing top-3 prediction accuracy across all 11 enantiomorphic space group pairs: (a) 76 vs. 78, (b) 91 vs.

95, (c) 92 vs. 96, (d) 144 vs. 145, (e) 151 vs. 153, (f) 152 vs. 154, (g) 169 vs. 170, (h) 171 vs. 172, (i) 178 vs. 179, (j) 180 vs. 181, and (k) 212 vs. 213. Each subfigure displays a confusion matrix evaluating the model's ability to classify the two space groups within each pair based on the top-3 predictions. Enantiomorphic space groups, which are chiral mirror images of each other, produce nearly identical diffraction patterns due to Friedel's Law, making accurate classification challenging. Compared to top-1 predictions, the top-3 prediction matrices exhibit lower misclassification rates, as indicated by higher diagonal element percentages (correct predictions) and reduced off-diagonal element values (misclassifications). These results demonstrate that, even when the model's first prediction is incorrect, it typically correctly identifies the true space group in its second or third predictions.

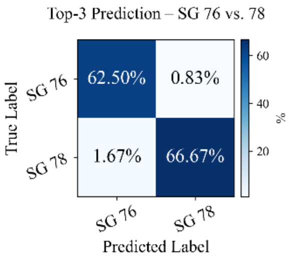

(a) Top-3 prediction,
Space group 76_vs._78

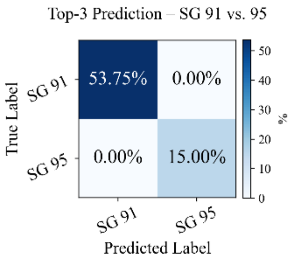

(b) Top-3 prediction,
Space group 91_vs._95

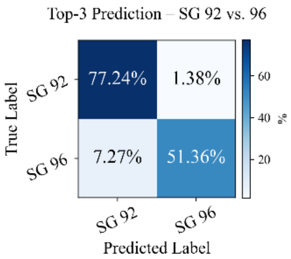

(c) Top-3 prediction,
Space group 92_vs._96

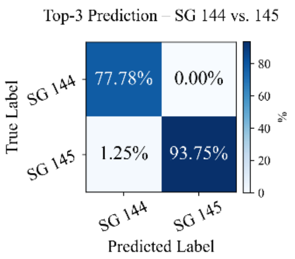

(d) Top-3 prediction,
Space group 144_vs._145

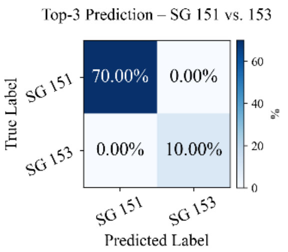

(e) Top-3 prediction,
Space group 151_vs._153

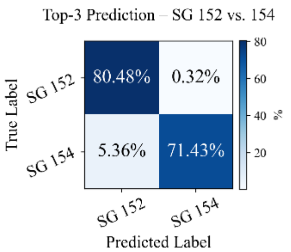

(f) Top-3 prediction,
Space group 152_vs._154

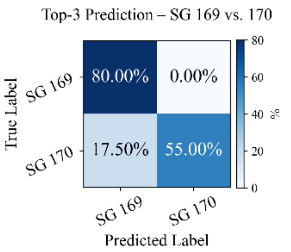

(g) Top-3 prediction,
Space group 169_vs._170

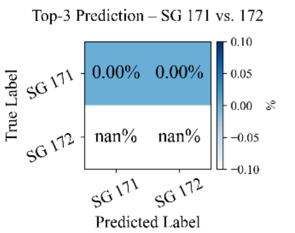

(h) Top-3 prediction,
171_vs._172.

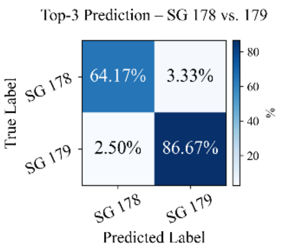

(i) Top-3 prediction,
Space group 178_vs._179

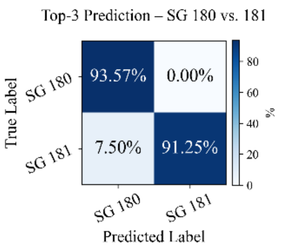

(j) Top-3 prediction,
Space group 180_vs._181

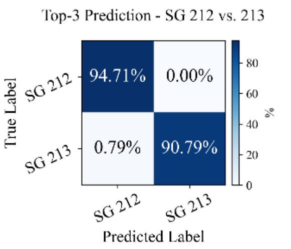

(k) Top-3 prediction,
Space group 212 vs. 213

Supplementary Figure G The confusion matrix for Model-4 illustrates the top-3 prediction performance across 11 enantiomorphic space group pairs: (a) 76 vs. 78, (b) 91 vs. 95, (c) 92 vs. 96, (d) 144 vs. 145, (e) 151 vs. 153, (f) 152 vs. 154, (g) 169 vs. 170, (h) 171 vs. 172, (i) 178 vs. 179, (j) 180 vs. 181, and (k) 212 vs. 213. The matrix highlights a reduction in misclassification.

Supplementary Figure H (1–9) presents a series of confusion matrices illustrating the percentage accuracy of classifying the seven crystal systems using Models 2, 3, and 4, trained on datasets with 4, 10, and 20 zone axes, respectively, and tested on corresponding test datasets with 4 (G1, G4, G7), 10 (G2, G5, G8), and 20 (G3, G6, G9) zone axes.

Higher classification accuracies are observed in G1, G4, G5, G7, G8, and G9, whereas G2, G3, and G6 exhibit lower accuracies with increased misclassification rates. This discrepancy is attributed to the presence of zone axes in the test datasets that were not included in the training datasets. For instance, in G2, Model-2 was trained on a dataset comprising 4 zone axes but tested on a dataset with 10 zone axes, resulting in 6 unseen zone axes during testing. Similarly, in G6, Model-3 was trained on 10 zone axes but evaluated on a 20-zone axis test dataset, introducing 10 unseen zone axes. These unseen zone axes directly contribute to the reduced classification performance in G2, G3, and G6, underscoring the models' dependence on comprehensive training data coverage and the necessity of aligning training and testing conditions for optimal performance.

Model-2

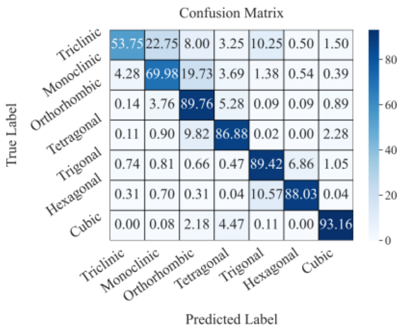

(1) trained on 4 zone and tested on 4 zone axes

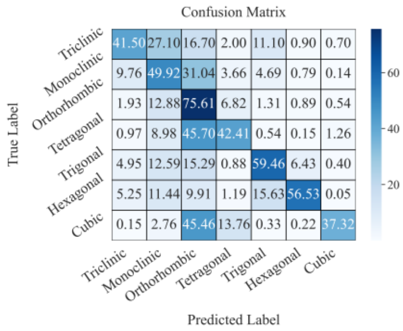

(2) trained on 4 zone and tested on 10 zone axes

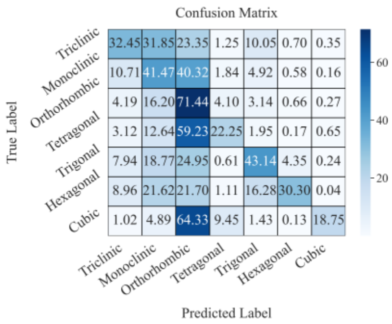

(3) trained on 4 zone and tested on 20 zone axes

Model-3

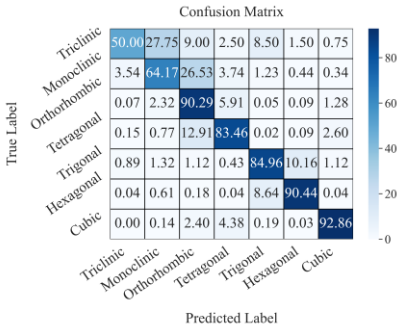

(4) trained on 10 zone and tested on 4 zone axes

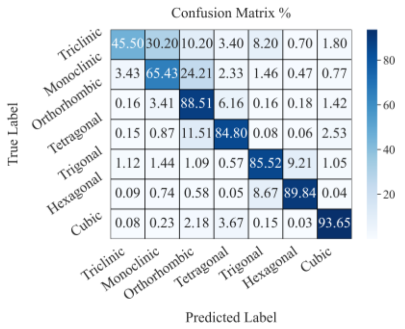

(5) trained on 10 zone and tested on 10 zone axes

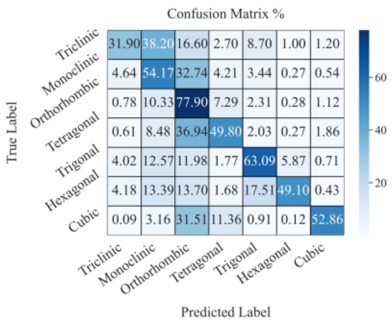

(6) trained on 10 zone and tested on 20 zone axes

Model-4

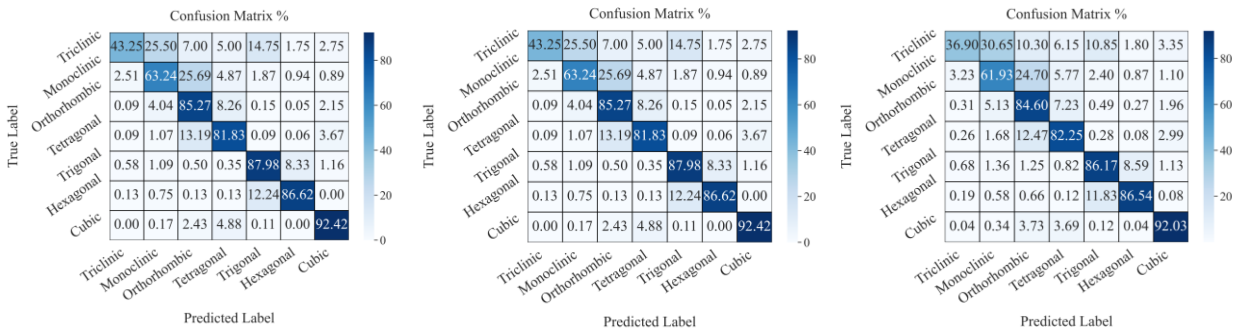

(7) trained on 20 zone and tested on 4 zone axes (8) trained on 20 zone and tested on 10 zone axes (9) trained on 20 zone and tested on 20 zone axes

Supplementary Figure H Confusion matrix based on the percentage of accuracy prediction for predicting 7 crystal systems, where the model was trained on 4, 10, and 20 zone axis (model 2, 3 and 4) and tested on 4 (H1,G 4 and G7), 10 (I2, G5 and G8) and 20 (J3, G6 and G9) zone test datasets.

Supplementary Figure I(1–9), similar to Supplementary Figure H(1-9), presents a series of confusion matrices for classifying the seven crystal systems using Models 2, 3, and 4, trained on datasets with 4, 10, and 20 zone axes, respectively, and tested on corresponding test datasets with 4 (H1, H4, H7), 10 (H2, H5, H8), and 20 (H3, H6, H9) zone axes. However, while Supplementary Figure H illustrates classification performance based on percentage accuracy, Supplementary Figure I is based on the number of correct and incorrect predictions, providing a quantitative perspective on model performance across varying training and testing conditions.

Model-2

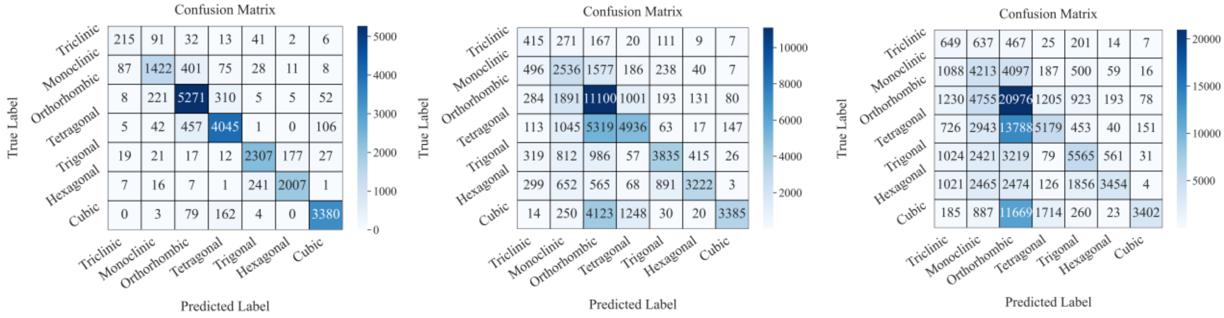

(1) trained on 4 zone and tested on 4 zone axis (2) trained on 4 zone and tested on 10 zone axis (3) trained on 4 zone and tested on 20 zone axis

Model-3

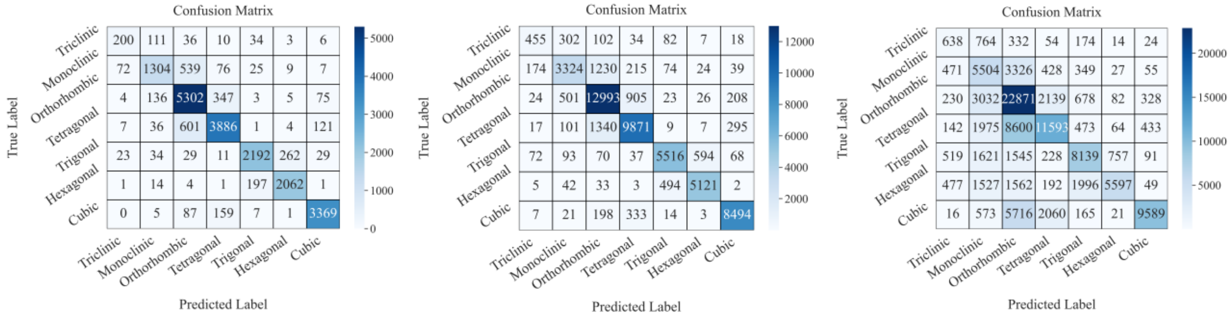

(4) trained on 10 zone and tested on 4 zone axis

(5) trained on 10 zone and tested on 10 zone axis

(6) trained on 10 zone and tested on 20 zone axis

Model-4

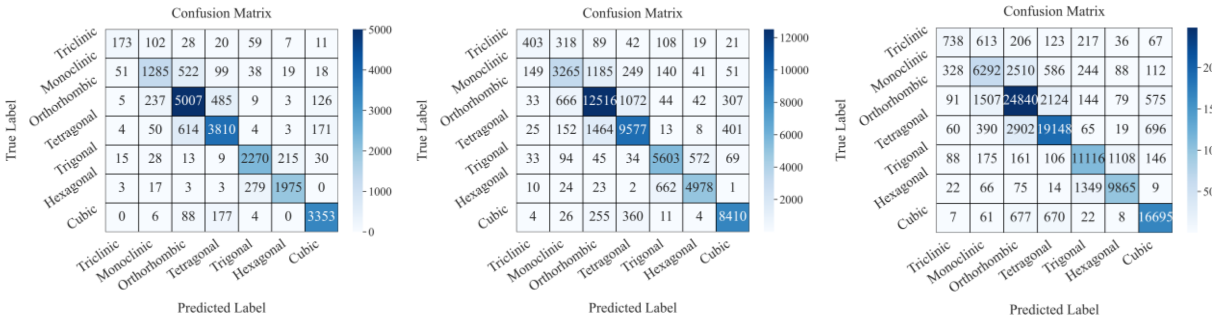

(7) trained on 20 zone and tested on 4 zone axis

(8) trained on 20 zone and tested on 10 zone axis

(9) trained on 20 zone and tested on 20 zone axis

Supplementary Figure I Confusion matrix based on the number of correct and incorrect prediction for predicting 7 crystal systems, where the models were trained on 4, 10, and 20 zone axis (model 2, 3 and 4) and tested on 4 (H1, H4 and H7), 10 (H2, H5 and H8) and 20 (H3, H6 and H9) zone test datasets.

Supplementary Table A and Supplementary Table B present the results of evaluating Model-4 on the test dataset comprising 20 zone axes for classifying 230 space groups. Supplementary Table A details the number of space groups containing fewer than or equal to 5, 10, 20, 30, 40, 50, 100, 150, and 200 CIFs, along with the average classification accuracy of these space groups in identifying the 230 space groups. Similarly, Supplementary Table B indicates the number of space groups with more than or equal to 300, 350, 400, and 450 CIFs, accompanied by their average accuracy in classifying the 230 space groups. This clearly shows that, generally, more CIFs per space group result in higher classification accuracy for the space group.

Supplementary Table A Number of space groups with fewer than or equal to 5, 10, 20, 30, 40, 50, 100, 150, and 200 CIFs and their average classification accuracy by Model-4 across 230 space groups, evaluated on a test dataset of 20 zone axes.

Number of CIFs <=	5	10	20	30	40	50	100	150	200
Number of Space Groups	10	21	30	44	55	70	98	110	129
Average accuracy	0.19%	25.24%	28.36%	32.61%	34.39%	37.32%	41.29%	42.83%	44.84%

Supplementary Table B Number of space groups with more than or equal to 300, 350, 400, and 450 CIFs and their average classification accuracy by Model-4 across 230 space groups, evaluated on a test dataset of 20 zone axes.

Number of CIFs >=	300	350	400	450
Number of Space Groups	83	76	69	66
Average accuracy	62.67%	62.88%	62.29%	62.37%

Supplementary Figure J depicts the classification performance of Models 2, 3, and 4 across the 230 space groups, with accuracy reported for each space group. These figures highlight the percentage of correct predictions for all 230 space groups. The models were trained on datasets comprising 4, 10, and 20 zone axes and evaluated using patterns from test datasets corresponding to 4 (L1, L4, L7), 10 (L2, L5, L8), and 20 (L3, L6, L9) zone axes. The figures illustrate the distribution of classification accuracy across the 230 space groups.

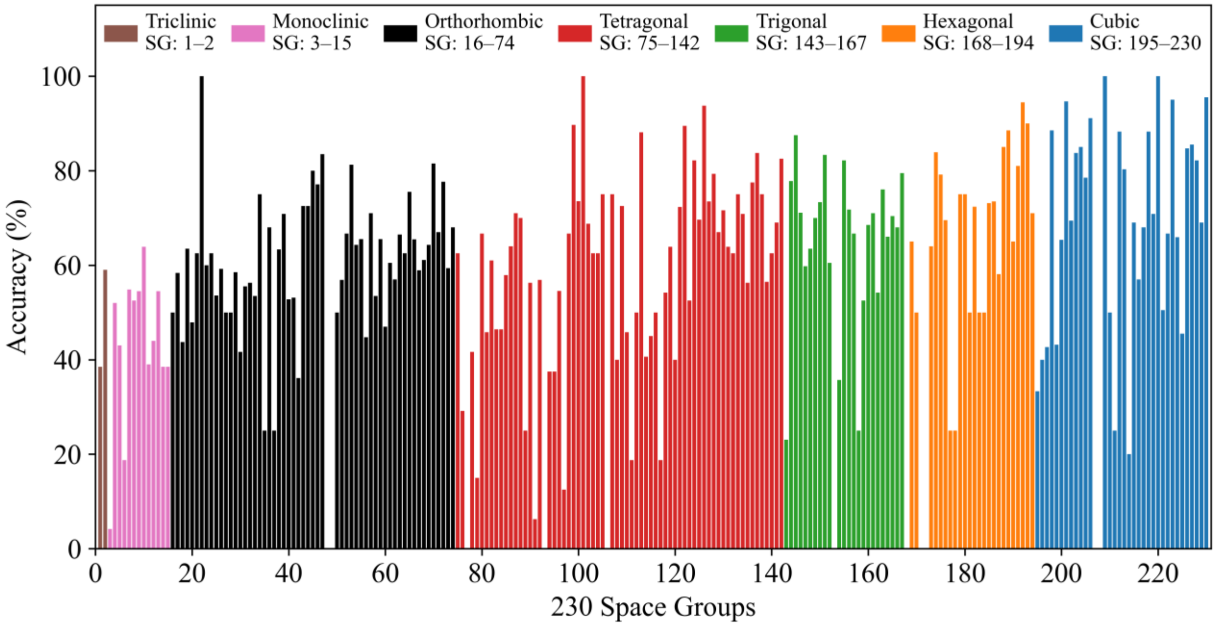

(1) model-2 trained on 4 zone and tested on 4 zone axes.

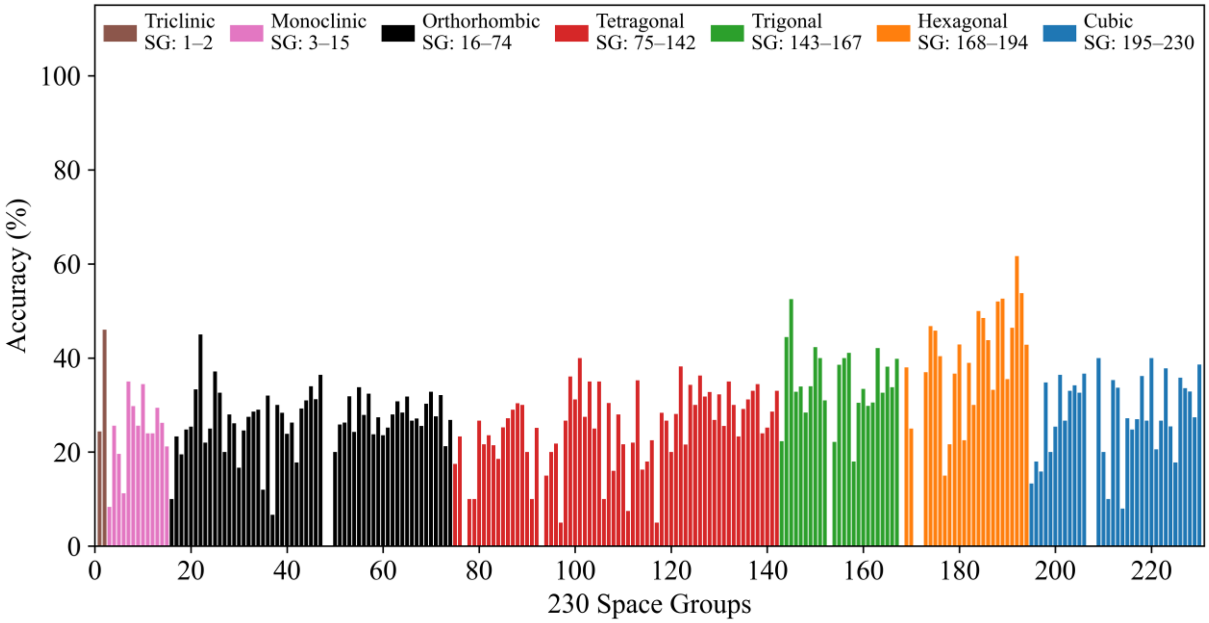

(2) model-2 trained on 4 zone and tested on 10 zone axes.

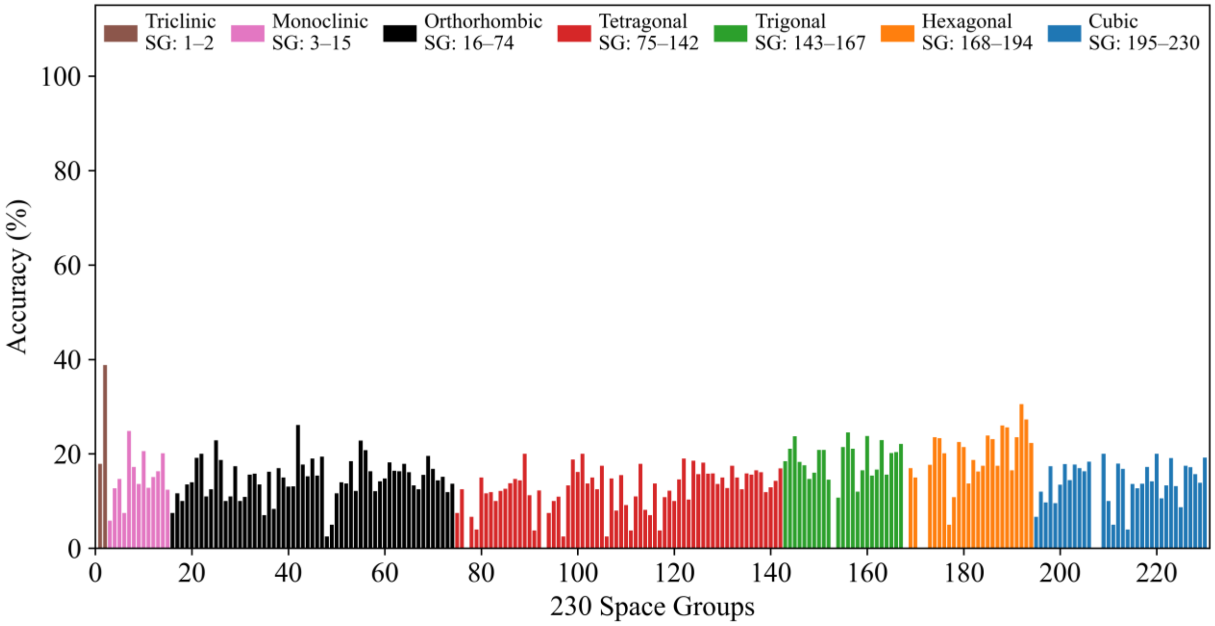

(3) model-2 trained on 4 zone and tested on 20 zone axes.

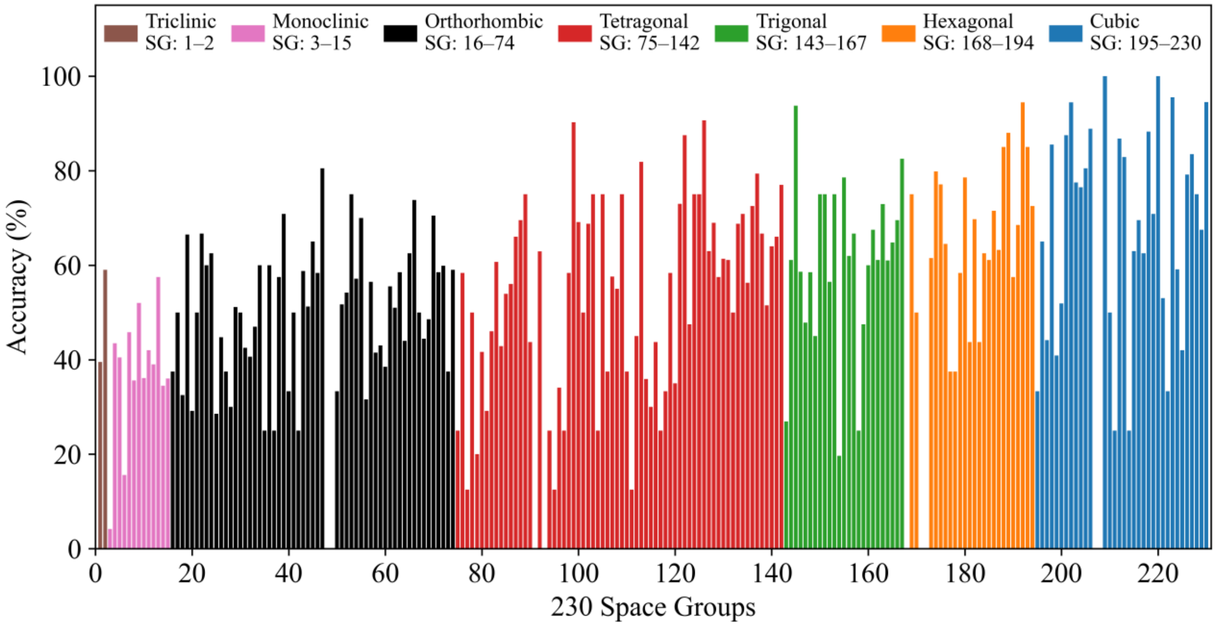

(4) model-3 trained on 10 zone and tested on 4 zone axes.

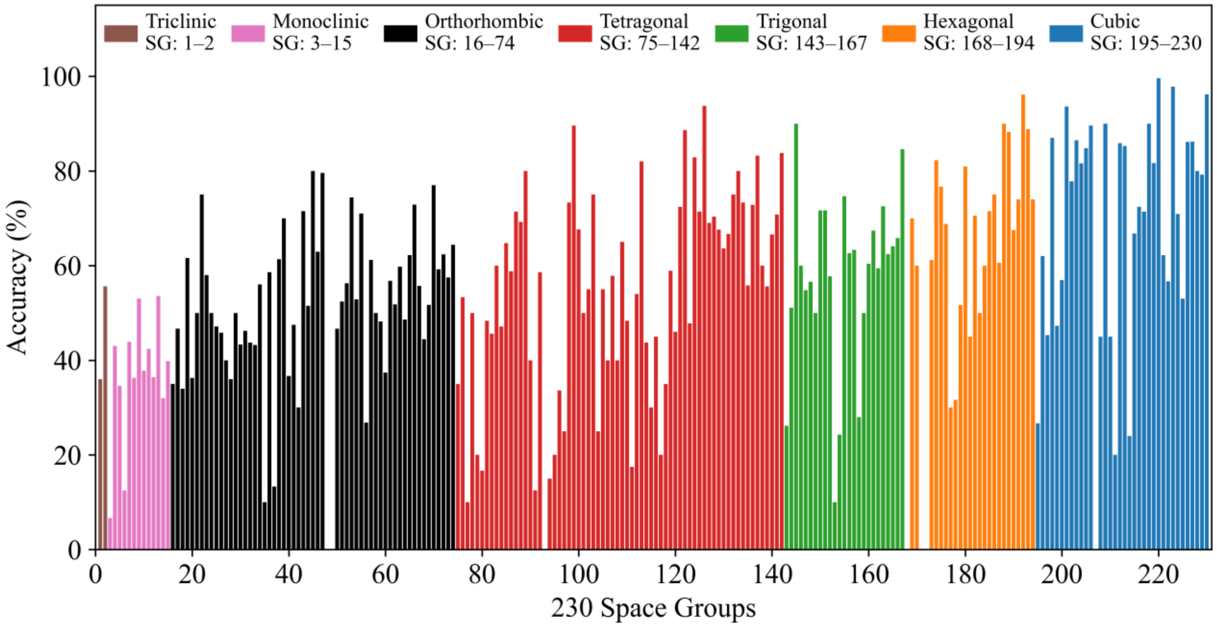

(5) model-3 trained on 10 zone and tested on 10 zone axes.

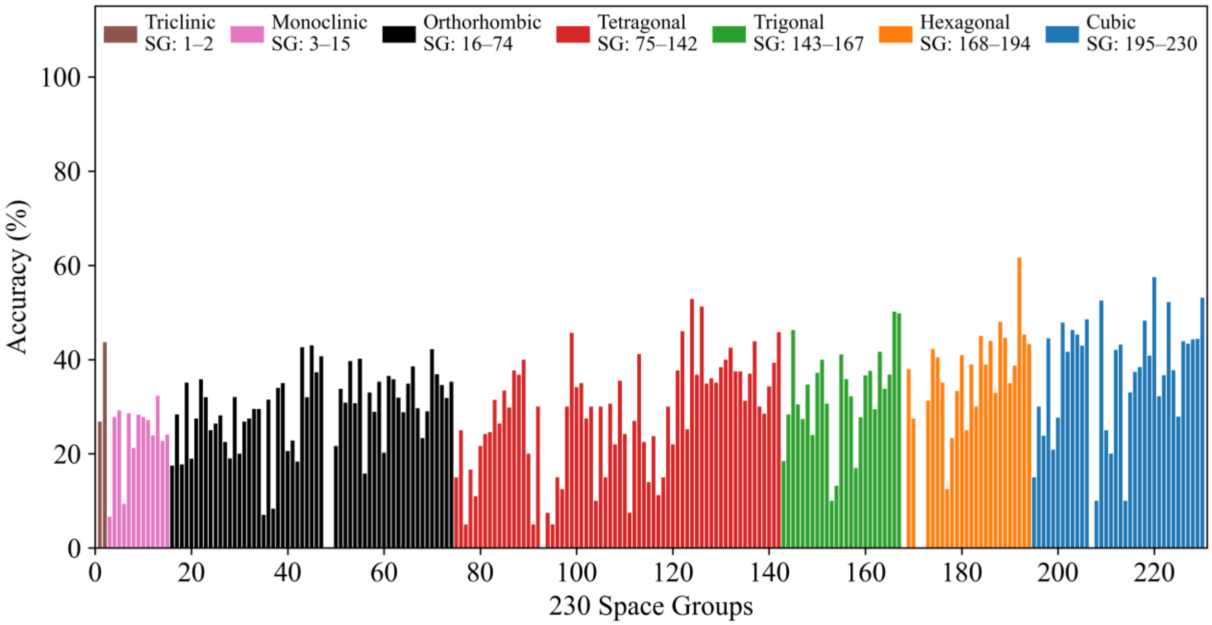

(6) model-3 trained on 10 zone and tested on 20 zone axes.

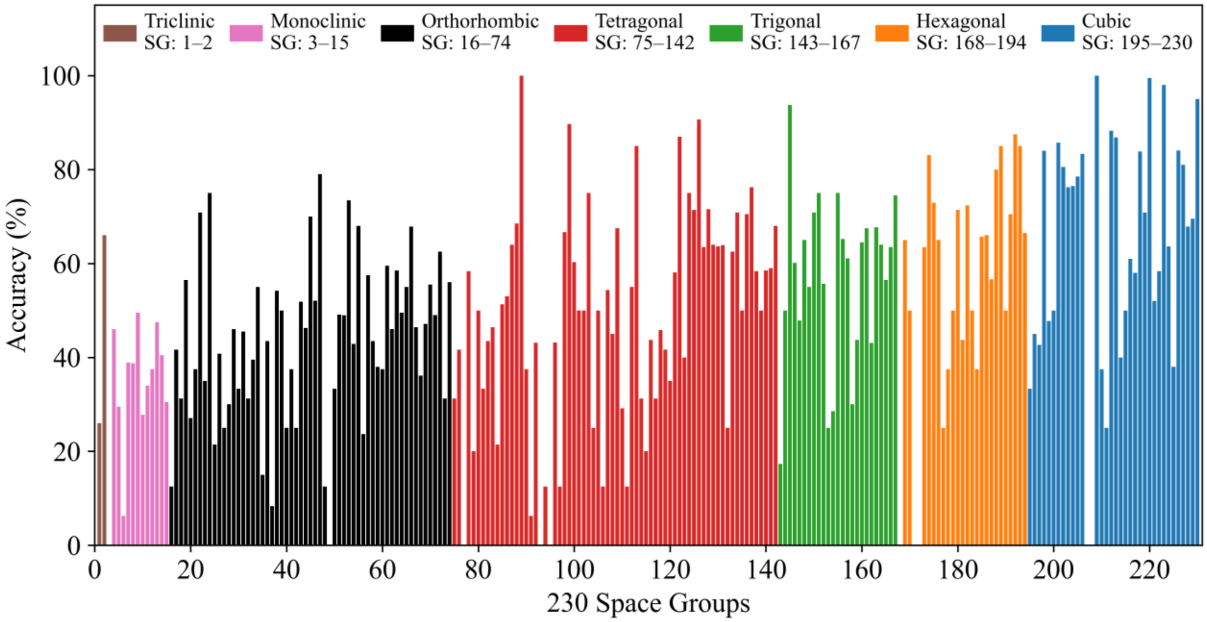

(7) model-4 trained on 20 zone and tested on 4 zone axes.

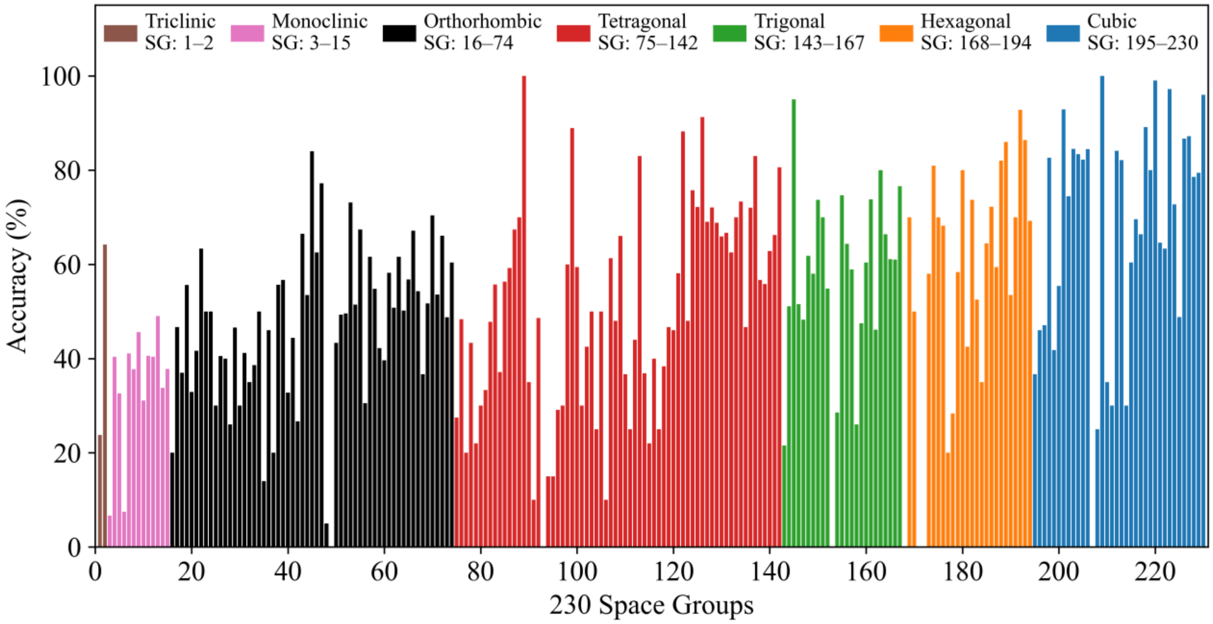

(8) model-4 trained on 20 zone and tested on 10 zone axes.

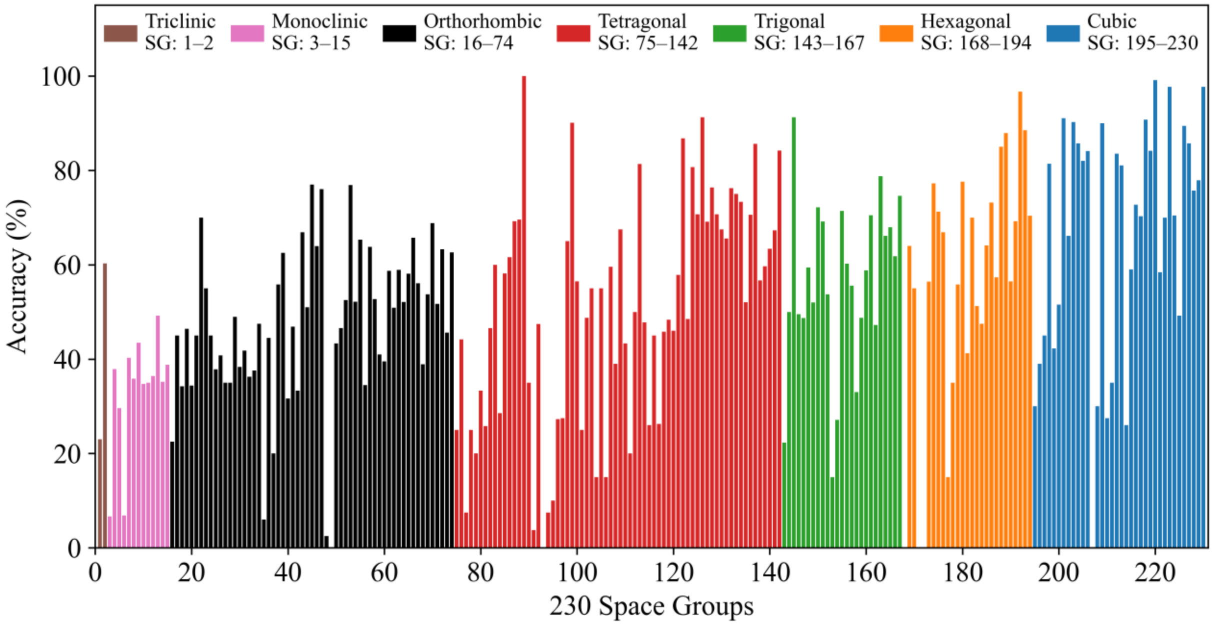

(9) model-4 trained on 20 zone and tested on 20 zone axes.

Supplementary Figure J(1-9) presents the classification accuracy across all 230 space groups. The evaluation is performed on test datasets comprising 4, 10, and 20 zone axes, using models trained on corresponding datasets with 4, 10, and 20 zone axes, respectively.

Supplementary Table C presents the classification accuracy of Model-4 for identifying seven crystal systems and 230 space groups based on 10,000 2D XRD patterns generated under the [100] zone axis. These patterns were derived from randomly selected CIFs from the training dataset, with one set comprising the original patterns and the other set featuring patterns subjected to random in-plane rotation around the center. The classification accuracy results indicate that Model-4 achieved 89.28% for crystal systems and 73.88% for space groups in the original patterns, establishing its baseline performance on the training data. For the patterns with random rotation, the classification accuracy slightly decreased to 86.32% for crystal systems and 71.21% for space groups, demonstrating the model's robustness to rotational variations commonly observed in experimental setups or sample orientations.

Supplementary Table C Classification accuracy of Model-4 for seven crystal systems and 230 space groups on 10,000 2D XRD patterns under the [100] zone axis, comparing non-rotated patterns (training dataset) with patterns subjected to in-plane rotation around the center.

Zone Axis	Classification	10,000 patterns without rotation (originally used in the training dataset)		10,000 patterns with random rotation	
		Number of correct predictions	Accuracy percentage	Number of correct predictions	Accuracy percentage
[100]	7 crystal systems	8928	89.28%	8632	86.32%
	230 space groups	7388	73.88%	7121	71.21%

Supplementary Table D presents the same family of zone axes, including the unseen zones [211], [011], [120], and [131], classified according to the seven crystal systems. Zone axes denoted with an asterisk (*) indicate those included in the training dataset.

Supplementary Table D we observe the same family of zone axes, including the unseen zones [211, 011, 120, and 131], categorized based on the seven crystal systems. The zone axes marked with an asterisk (*) indicate those that are present in our training dataset.

7 Crystal System	The same family of zone axes			
	Unseen zone [120]	Unseen zone [211]	Unseen zone [011]	Unseen zone [131]
Triclinic	120	211	011	131
Monoclinic	120	211	011	131
Orthorhombic	120	211	011	131
Tetragonal	120<br>210*	121*<br>211	011<br>101*	131<br>311
Trigonal	1 2 0<br>2 1 0*	1 2 1*<br>2 1 1	101*<br>011	311<br>131
Hexagonal	120<br>210*	121*<br>211	101*<br>011	311<br>131
Cubic	120<br>021<br>210*<br>102<br>201<br>012*	112*<br>121*<br>211	101*<br>110*<br>011	131<br>113<br>311

To evaluate the generalization capability of Model-4 beyond its training dataset, we conducted an experiment using four unseen zone axes: [131], [211], [011], and [120]. These zone axes were deliberately excluded from the training datasets to ensure that the model was tested on unfamiliar crystallographic orientations. The test dataset was derived from the test subset of the 52k CIFs dataset, which constitutes 10% of the total dataset (5,362 CIFs). Using the ADP, we generated 2D XRD patterns for each of the four

unseen zone axes. This process yielded a total of 21,448 unique 2D XRD patterns, calculated as 5,362 test CIFs multiplied by the four zone axes.

Supplementary Figure K illustrates that the classification accuracy for space groups corresponding to the unseen zone axis [131] is significantly lower than that for the zone axes [211], [011], and [120]. This discrepancy arises because none of the 20 zone axes used in training Model-4 belong to the same zone family (i.e., symmetrically equivalent directions) as the unseen zone axis [131]. In contrast, the zone axes [211], [011], and [120] have equivalent zone families represented in the training dataset. These findings suggest that incorporating a more diverse set of zone axes in the training dataset could improve classification accuracy for unseen zone axes by increasing the probability of including symmetrically equivalent zone families.

As shown in Supplementary Figure K and detailed in Supplementary Table D, the zone axes [211], [011], and [120] for the cubic crystal system exhibit two symmetrically equivalent directions, a higher number compared to other crystal systems. Consequently, space groups associated with the cubic crystal system (space groups 95 to 230) generally demonstrate higher classification accuracy. This enhanced accuracy is attributed to the increased presence of symmetrically equivalent directions in the training dataset for these zone axes.

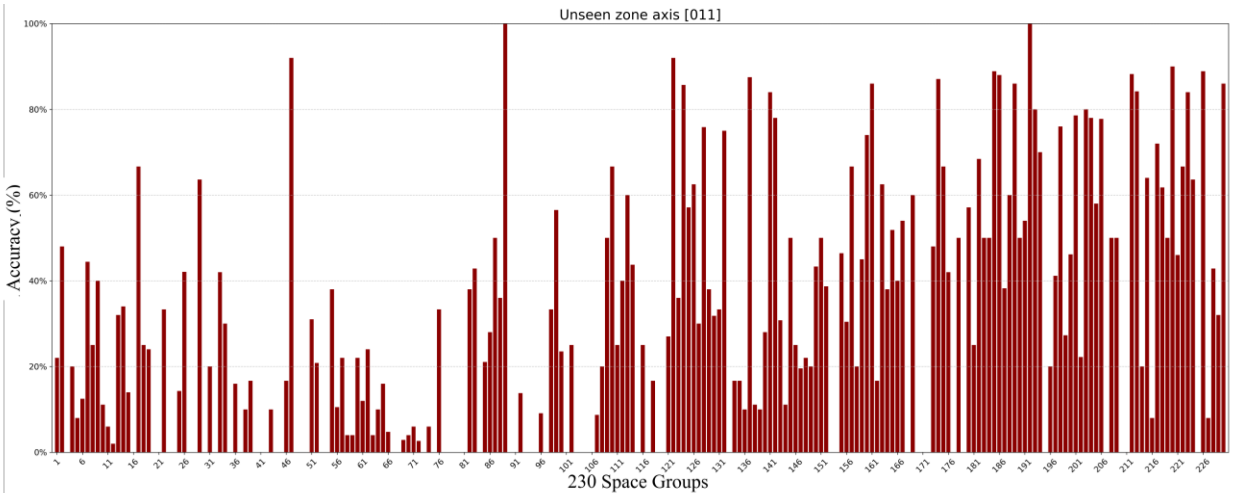

(1) Model-4 trained on 20 zone and tested on unseen zone axis [011]

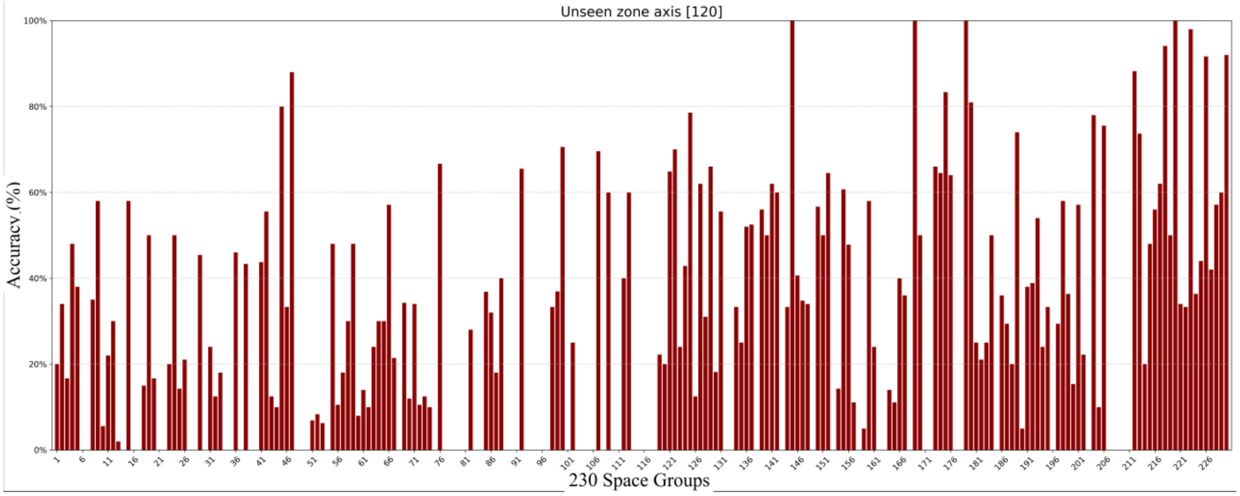

(2) Model-4 trained on 20 zone and tested on unseen zone axis [120]

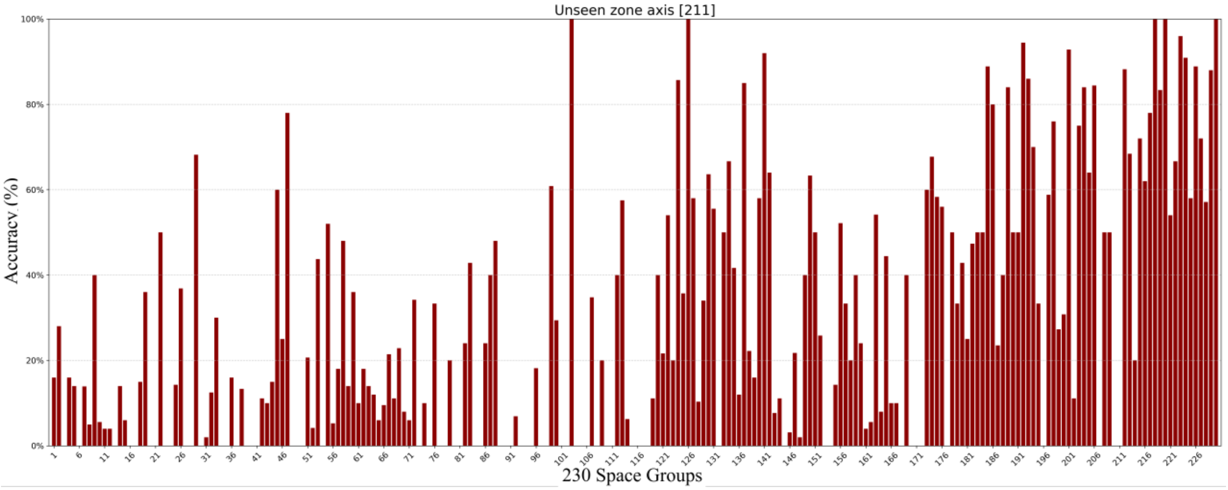

(3) Model-4 trained on 20 zone and tested on unseen zone axis [211]

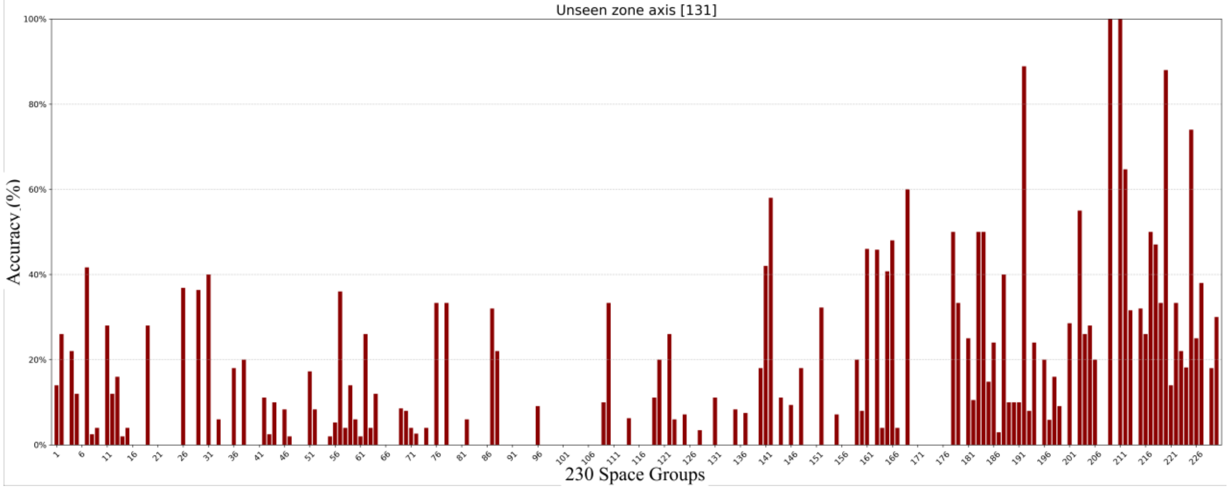

(4) Model-4 trained on 20 zone and tested on unseen zone axis [131]

Supplementary Figure K Classification accuracy across 230 space groups under unseen zone axes (1) [011], (2) [120], (3) [211], and (4) [131].

To assess the robustness of Model-4, trained on the 20 zone axes dataset, we investigated its performance under atomic substitution within the unit cell. Atomic substitutions modify the crystal's structure factor, altering the intensity of diffraction spots in 2D XRD patterns and introducing noise or perturbations. This evaluation aims to quantify the impact of such perturbations on the model's predictive performance.

For this experiment, we curated test datasets by selecting 6,305 CIFs from the training subset of the 52K dataset, encompassing all classes. To ensure sufficient atomic sites for substitution, only CIFs with unit cells containing more than 100 atoms were included. Random atomic substitutions were performed at levels of 0%, 1%, 2%, 5%, 10%, 25%, and 50% of the atoms in these structures. Replacement elements were randomly chosen from the following subset of the periodic table: H, He, Li, Be, B, C, N, O, F, Ne, Na, Mg, Al, Si, P, S, Cl, Ar, K, Ca, Sc, Ti, V, Cr, Mn, Fe, Co, Ni, Cu, Zn, Ga, Ge, As, Se, Br, Kr, Rb, Sr, Y, Zr, Nb, Mo, Tc, Ru, Rh, Pd, Ag, Cd, In, Sn, Sb, Te, I, Xe, Cs, Ba, La, Ce, Pr, Nd. This selection ensures a diverse range of chemical environments to robustly test the model's resilience to structural perturbations.

The ADP was utilized to generate 2D XRD patterns along the [100] and [111] zone axes for each substituted structure. This process produced a total of 88,270 synthetic 2D XRD patterns for testing, calculated as 6,305 CIFs × 2 zone axes × 7 substitution levels.

Supplementary Figure L illustrates the classification accuracy across seven crystal systems along the [100] and [111] zone axes under varying levels of atomic substitutions (1%, 2%, 5%, 10%, 25%, and 50%). Similarly, Supplementary Figure M depicts the classification accuracy for 230 space groups along the same zone axes at the specified substitution levels.

Supplementary Figure L presents the classification accuracy for the seven crystal systems, highlighting the influence of noise on Model-4's predictions. Although the model demonstrates adequate accuracy in classifying the seven crystal systems, the results indicate that systems such as cubic and hexagonal do not consistently exhibit the highest accuracy under conditions of noise and intensity variations induced by atomic substitutions.

In contrast, Supplementary Figure M, which depicts the classification accuracy for the 230 space groups, reveals a more pronounced effect of noise. The perturbations caused by noise and intensity changes significantly disrupt the symmetry operations within the structures, leading to greater challenges in accurately predicting the correct space groups compared to the classification of the seven crystal systems.

Zone Axis [100]

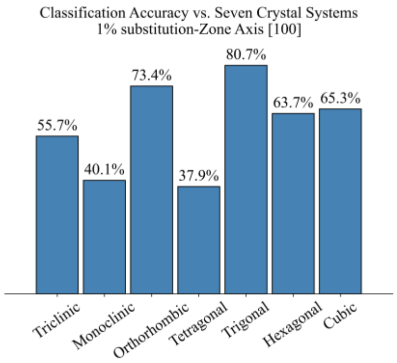

(1) 1% substitution, zone [100]

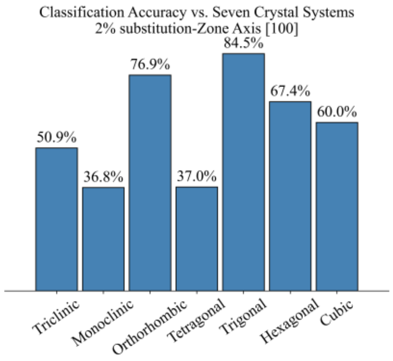

(2) 2% substitution, zone [100]

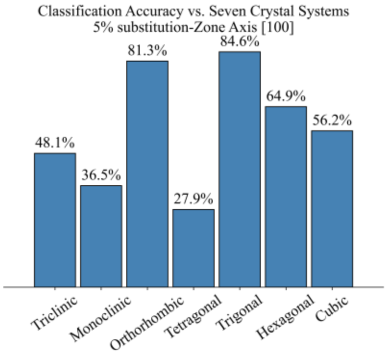

(3) 5% substitution, zone [100]

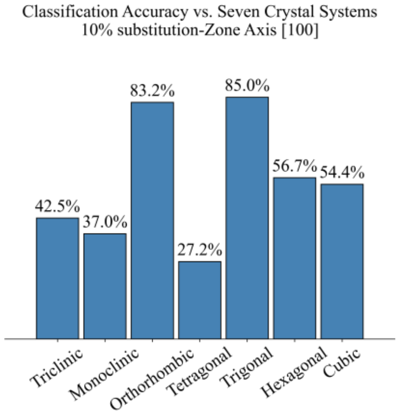

(4) 10% substitution, zone [100]

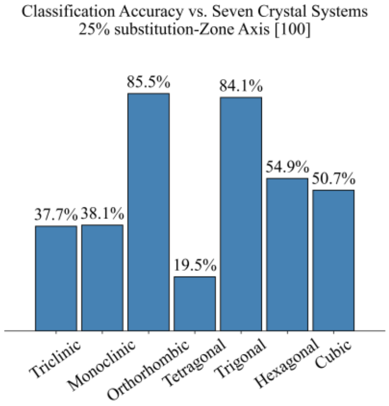

(5) 25% substitution, zone [100]

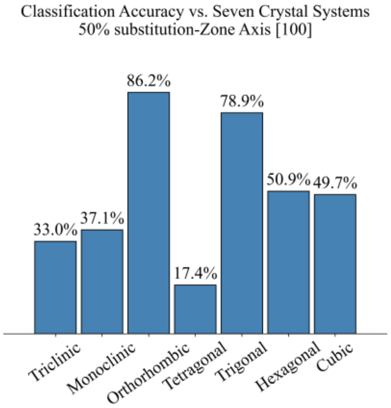

(6) 50% substitution, zone [100]

Zone Axis [111]

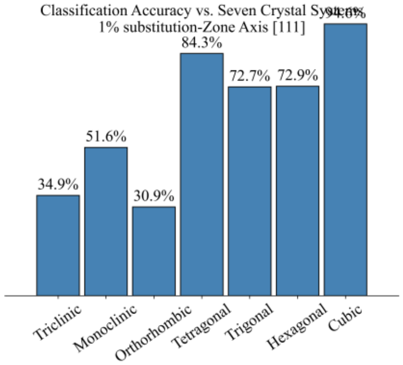

(7) 1% substitution, zone [111]

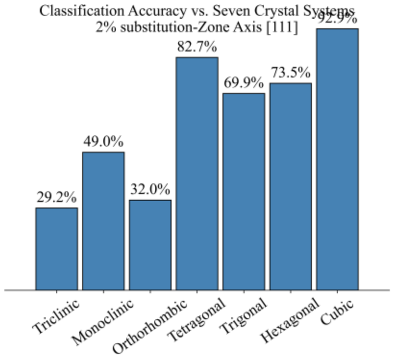

(8) 2% substitution, zone [111]

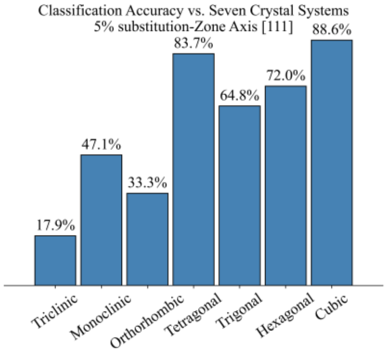

(9) 5% substitution, zone [111]

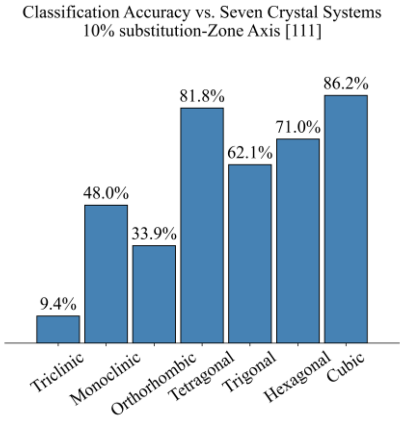

(10) 10% substitution, zone [111]

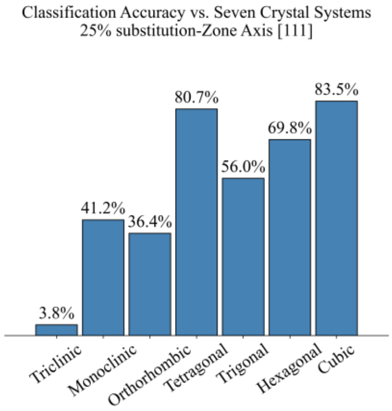

(11) 25% substitution, zone [111]

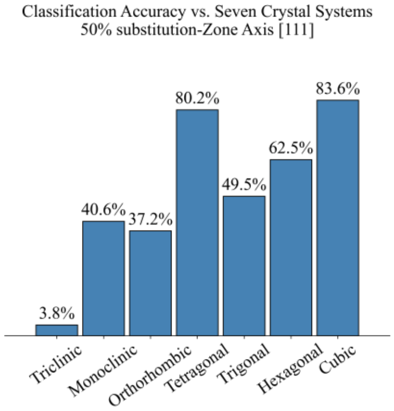

(12) 50% substitution, zone [111]

Supplementary Figure L Classification accuracy of Model 4 for each crystal system along the [100] zone axes (1–6) and the [111] zone axes (7–12), measured at 1%, 2%, 5%, 10%, 25% and 50% atomic substitution.

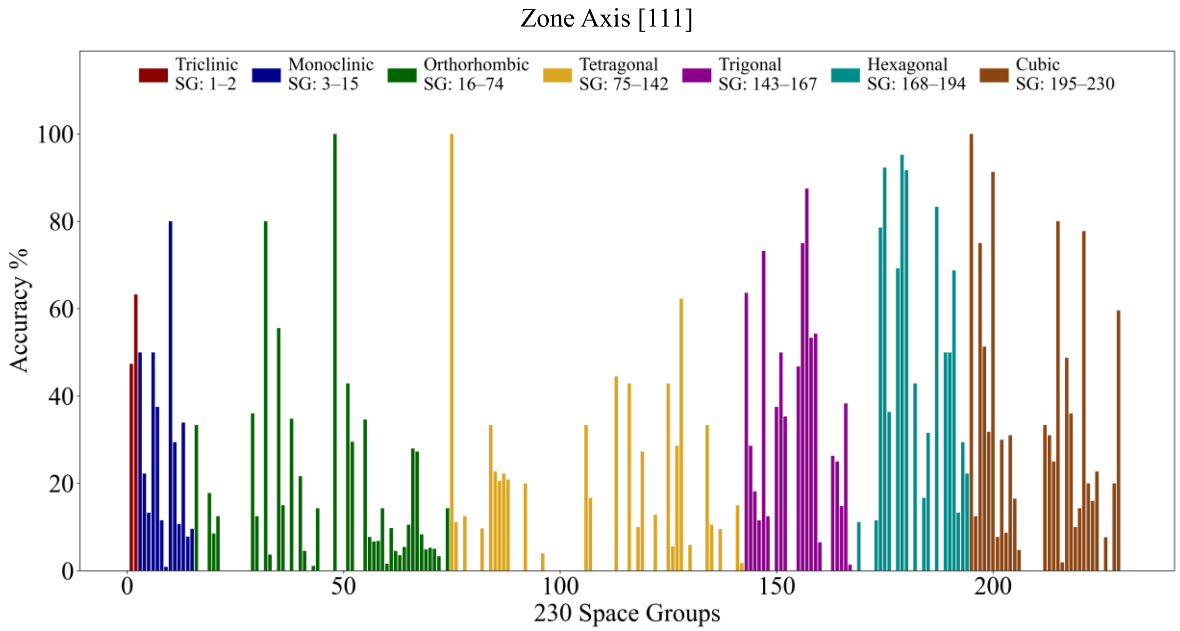

(1) 1% substitution, zone axis [100]

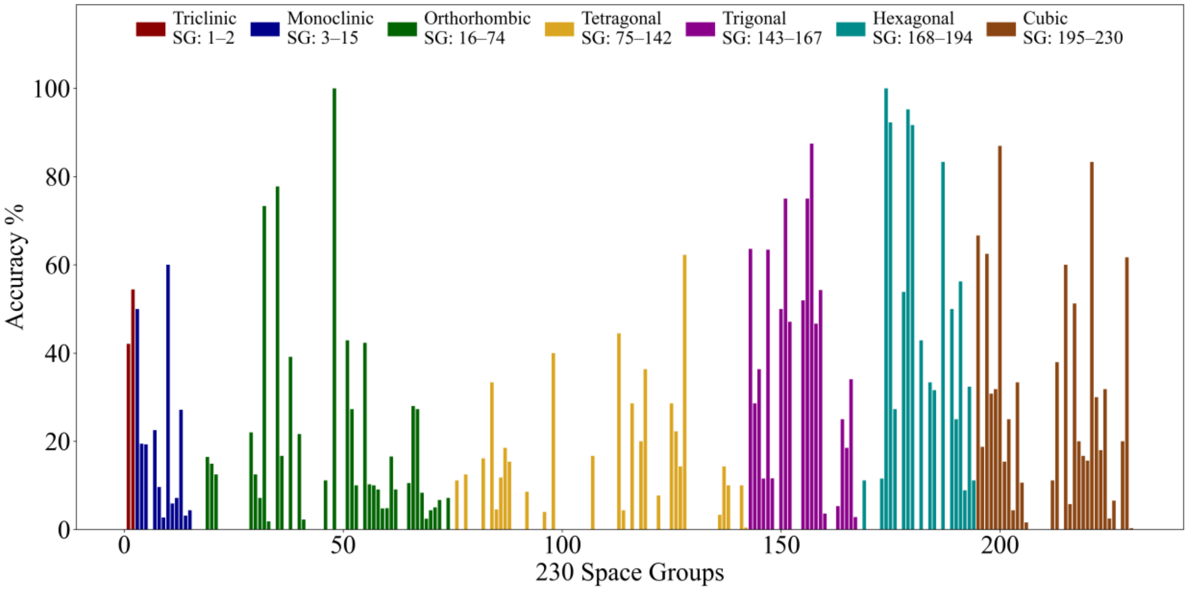

(2) 2% substitution, zone axis [100]

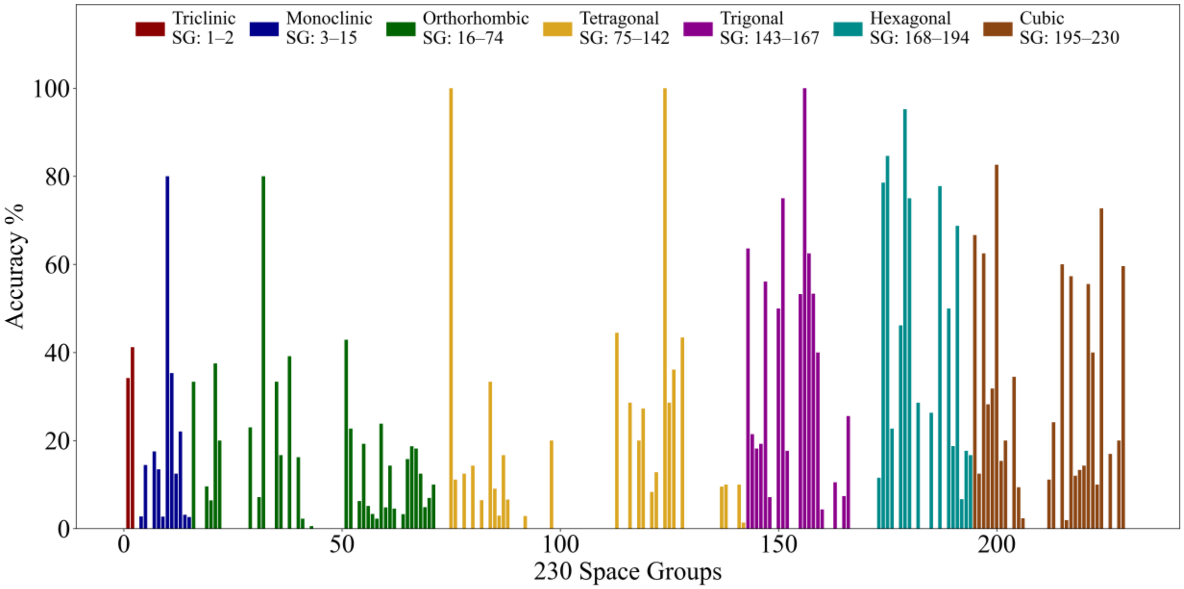

(3) 5% substitution, zone axis [100]

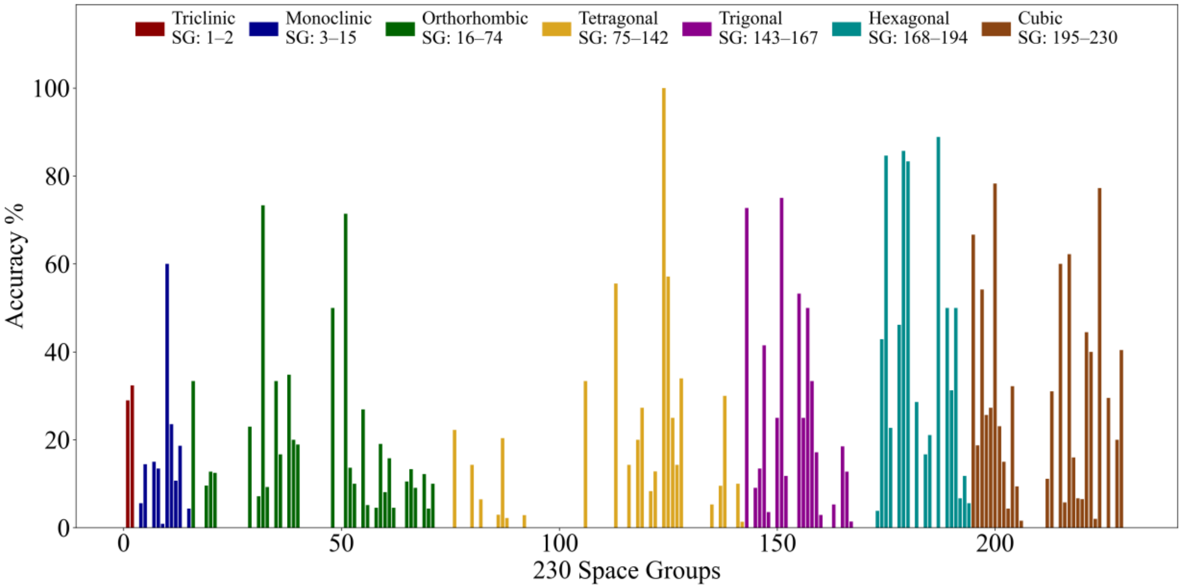

(4) 10% substitution, zone axis [100]

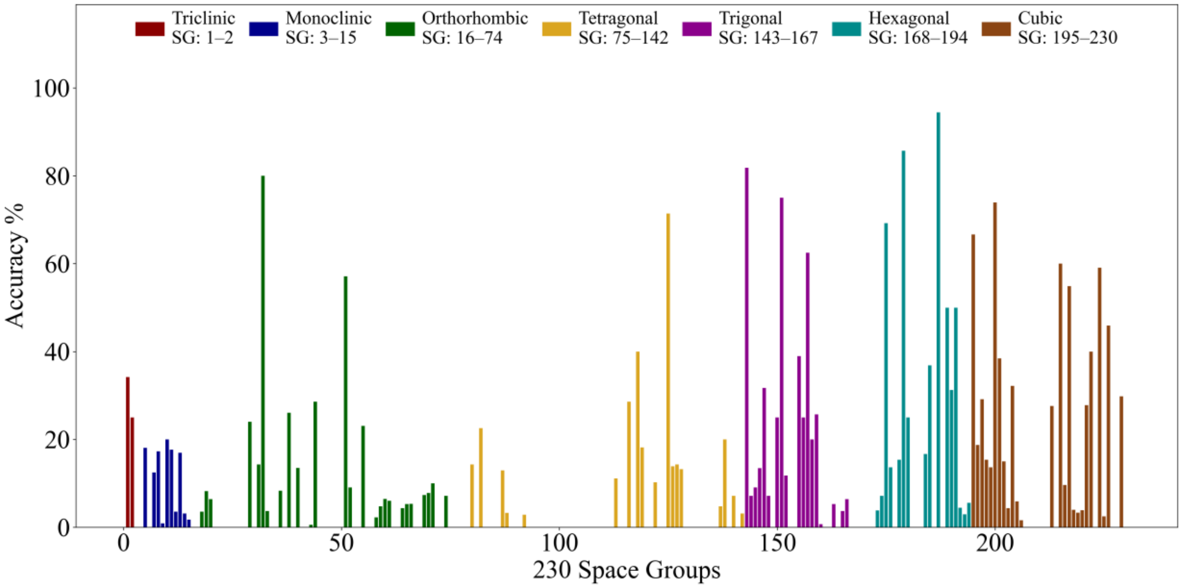

(5) 25% substitution, zone axis [100]

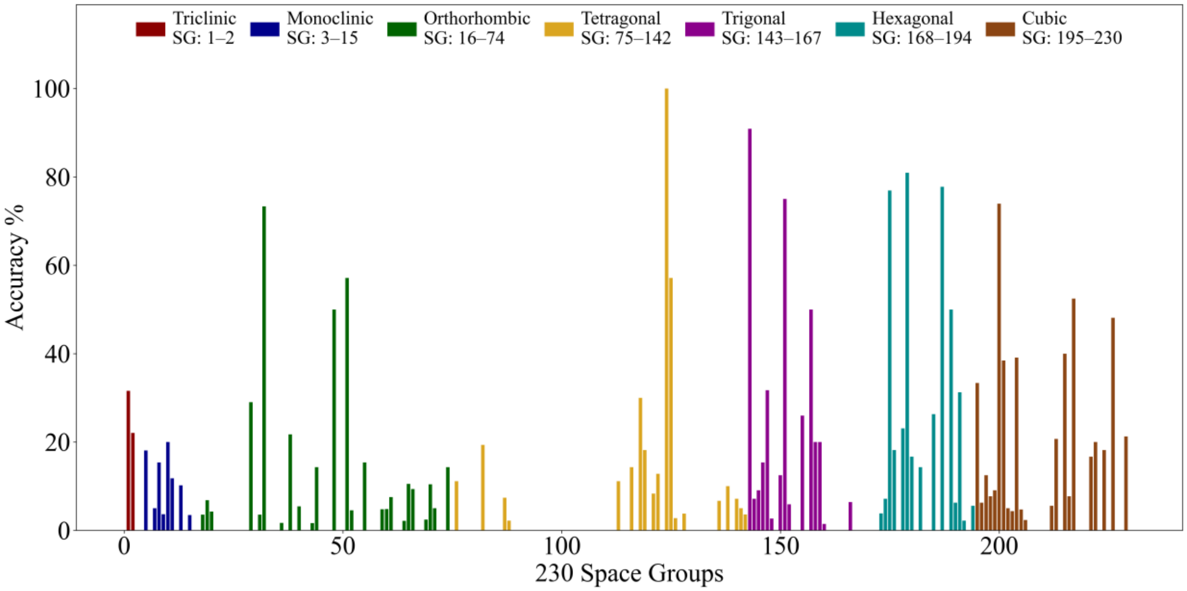

(6) 50% substitution, zone axis [100]

Zone Axis [111]

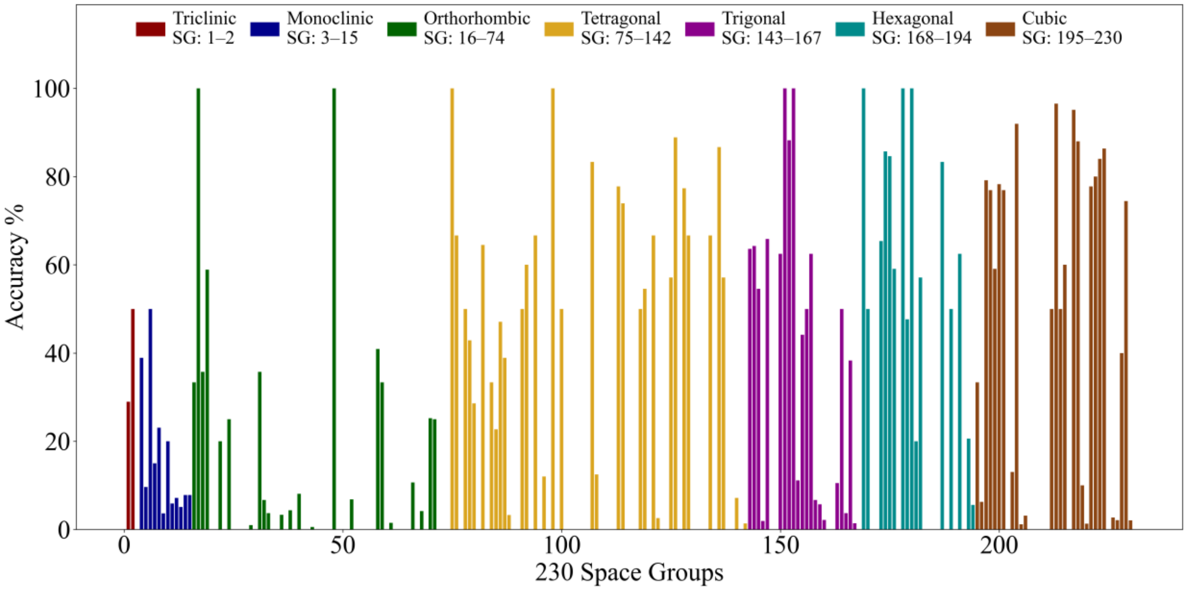

(7) 1% substitution, zone axis [111]

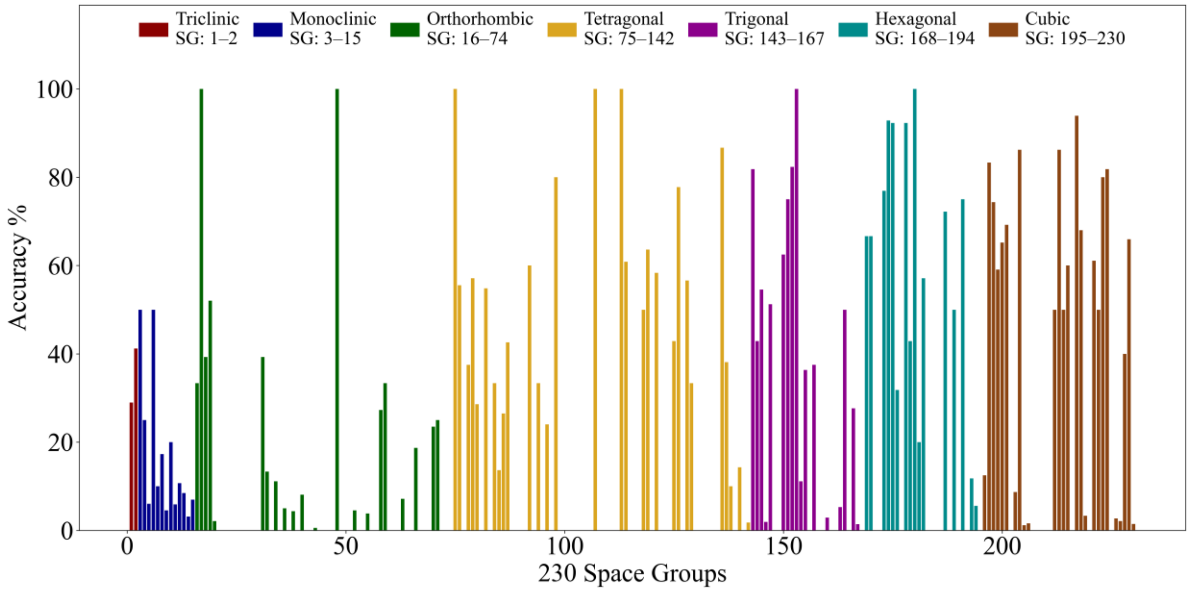

(8) 2% substitution, zone axis [111]

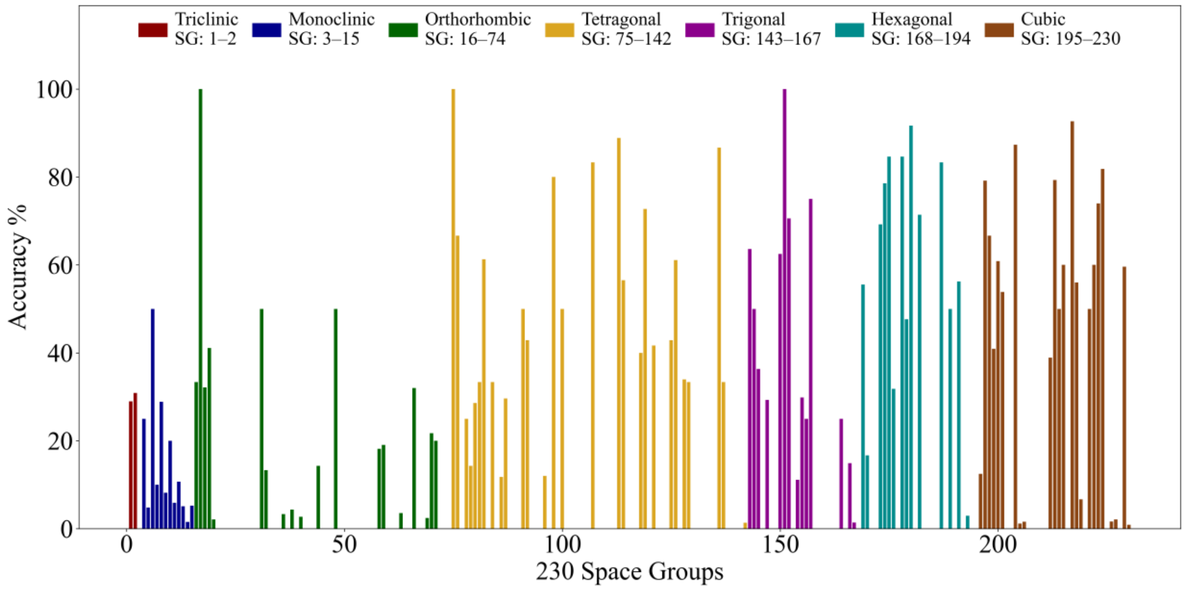

(9) 5% substitution, zone axis [111]

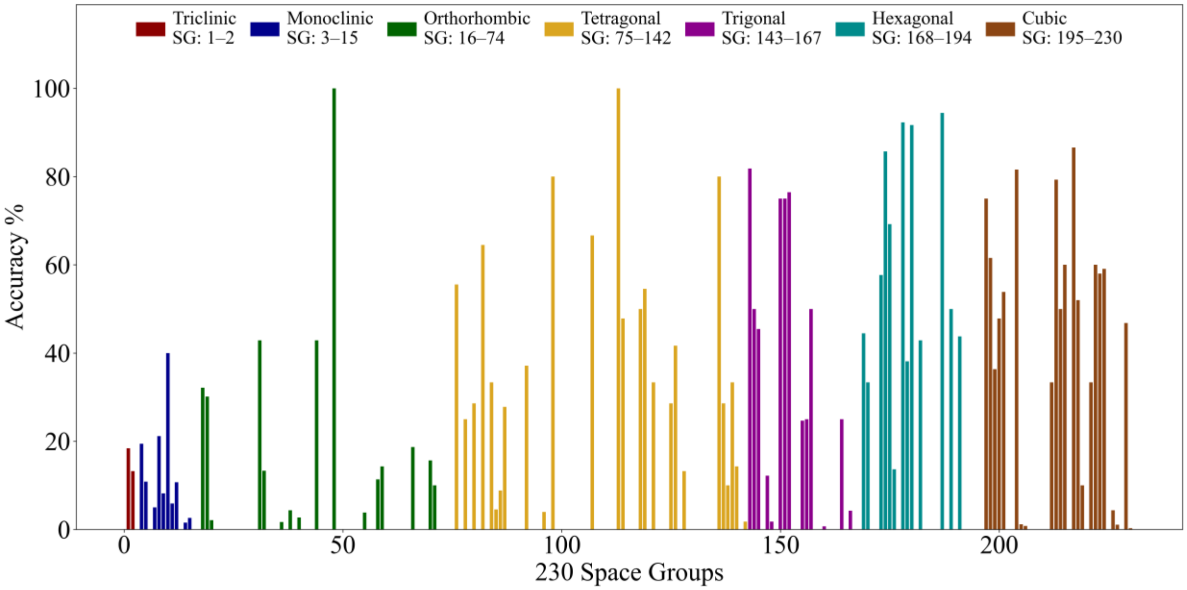

(10) 10% substitution, zone axis [111]

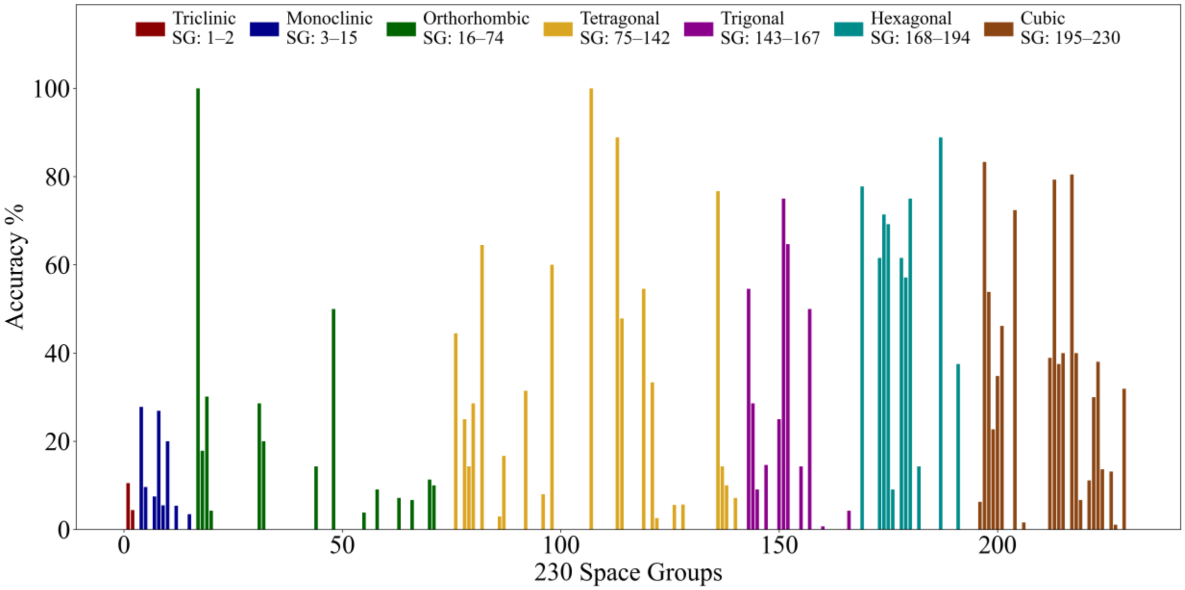

(11) 25% substitution, zone axis [111]

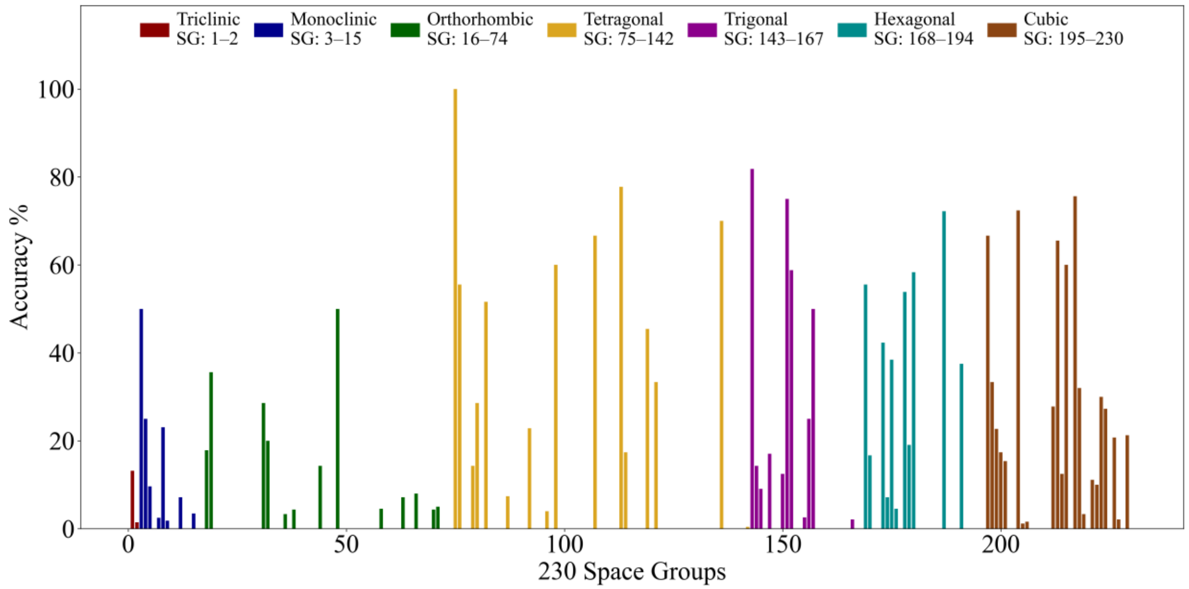

(12) 50% substitution, zone axis [111]

Supplementary Figure M Classification accuracy of Model 4 for 230 space groups along the [100] and [111] zone axes (indices 1–6 and 7–12, respectively), evaluated under 1%, 2%, 5%, 10%, 25%, and 50% atomic substitution.

In this experience, we investigated phase transition processes involving the transformation of a non-body-centered cubic (non-BCC) structure into a BCC structure and a non-face-centered cubic (non-FCC) structure into an FCC structure. To characterize these transitions, we generated synthetic 2D XRD patterns corresponding to atomic depletion levels of 0%, 25%, 50%, 75%, and 100% along two zone axes, [100] and [111]. For each phase transition (non-BCC to BCC and non-FCC to FCC), five CIFs were randomly selected. Supplementary Figure N through Supplementary Figure U (1–5) depict the structural evolution within the unit cells at each depletion stage, while Supplementary Figure N through Supplementary Figure U (6–10) display the associated synthetic 2D XRD patterns for the [100] and [111] zone axes at each depletion level.

In Supplementary Table E through Supplementary Table L, we evaluated space group predictions derived from Phonopy (based on POSCAR files) against those from our trained Model-4 (based on synthetic 2D XRD patterns). At 0% and 100% depletion levels, both Phonopy and Model-4 accurately identified the correct space groups, based on the 14 Bravais lattices. However, at intermediate depletion levels (25%, 50%, and 75%) for both [100] and [111] zone axes, Model-4 predicted space groups outside the cubic range (space groups 195–230), suggesting that progressive atomic depletion disrupts crystal symmetry. Similarly, Phonopy's predictions at these depletion levels indicated non-cubic space groups.

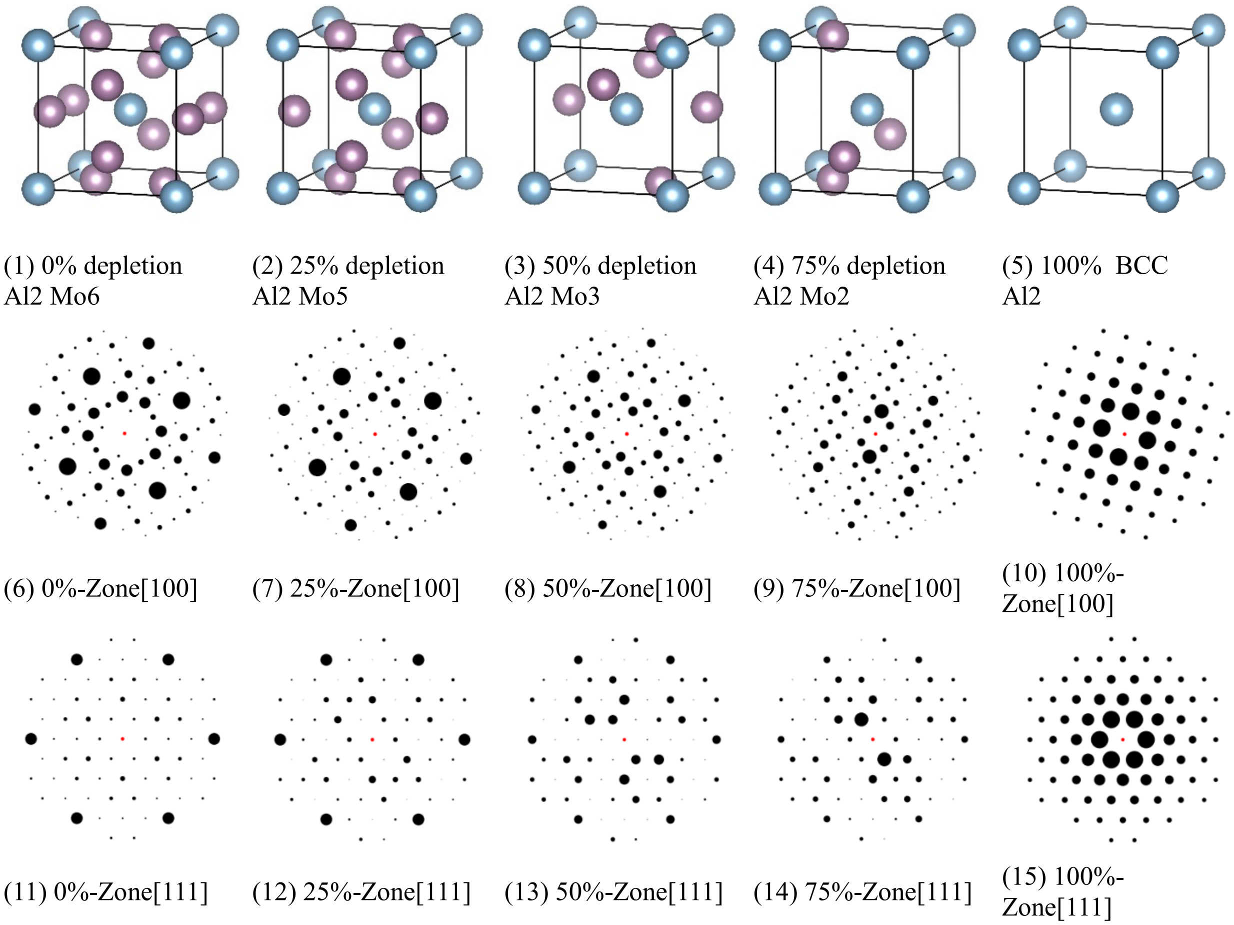

Supplementary Figure N (a–e) The unit cell of the structure (ICSD_057999) after applying defects. (f–j) 2D XRD patterns along the [100] zone axis under different defect percentages. (k–o) 2D XRD patterns along the [111] zone axis under different defect percentages.

Supplementary Table E The predictions of Phonopy and Model-4 under different defect percentages in the sample structure (ICSD_057999).

Depletion	0%	25%	50%	75%	100%
Chemical component	Al2 Mo6	Al2 Mo5	Al2 Mo3	Al2 Mo2	Al2
Phonopy classification	223	115	155	40	229
Model-4's predict - Zone 100	223	216	140	82	229
Model-4's predict - Zone 111	223	223	131	40	229

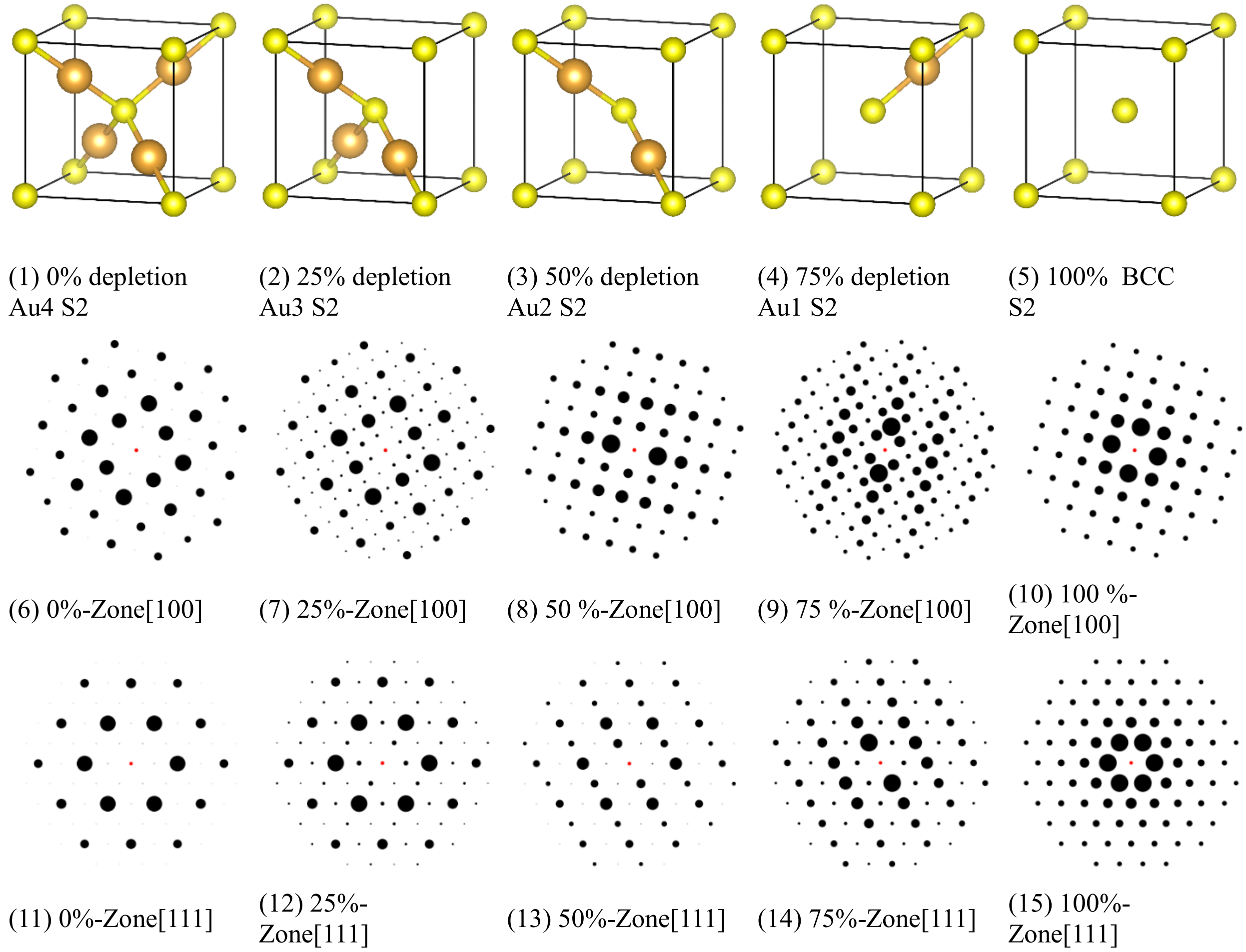

Supplementary Figure O (a–e) The unit cell of the structure (ICSD_ 078718) after applying defects. (f–j) 2D XRD patterns along the [100] zone axis under different defect percentages. (k–o) 2D XRD patterns along the [111] zone axis under different defect percentages.

Supplementary Table F The predictions of Phonopy and Model-4 under different defect percentages in the sample structure (ICSD_078718).

Depletion	0%	25%	50%	75%	100%
Chemical component	Au4 S2	Au3 S2	Au2 S2	Au1 S2	S2
Phonopy classification	224	166	67	166	229
Model-4's predict - Zone 100	224	215	139	70	229
Model-4's predict - Zone 111	224	216	99	225	229

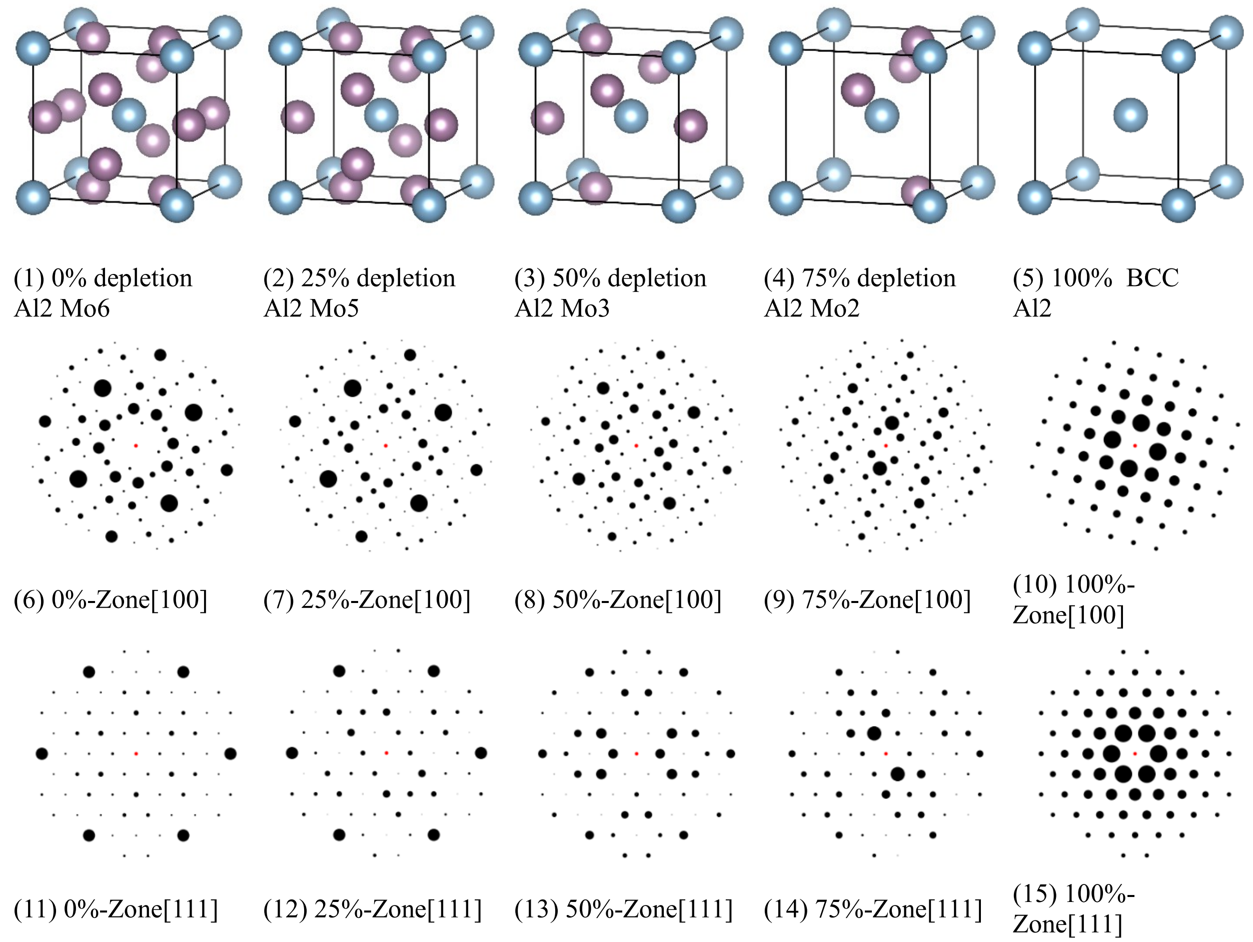

Supplementary Figure P (a–e) The unit cell of the structure (ICSD_ 608584) after applying defects. (f–j) 2D XRD patterns along the [100] zone axis under different defect percentages. (k–o) 2D XRD patterns along the [111] zone axis under different defect percentages.

Supplementary Table G The predictions of Phonopy and Model-4 under different defect percentages in the sample structure (ICSD_608584).

Depletion	0%	25%	50%	75%	100%
Chemical component	Al2 Mo6	Al2 Mo5	Al2 Mo3	Al2 Mo2	Al2
Phonopy classification	223	115	155	40	229
Model-4's predict - Zone 100	223	216	121	82	229
Model-4's predict - Zone 111	223	223	131	40	229

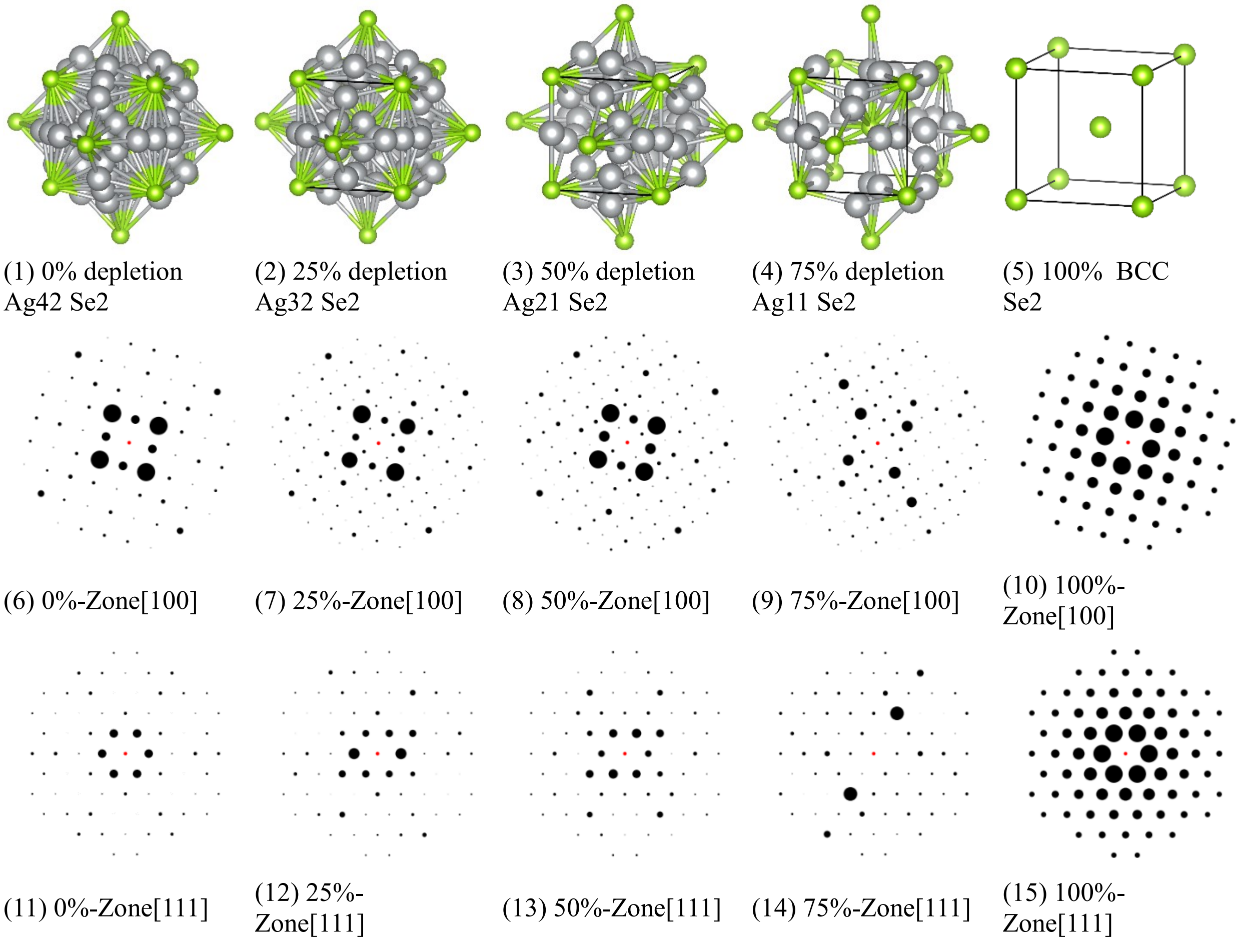

Supplementary Figure Q (a–e) The unit cell of the structure (ICSD_ 033627) after applying defects. (f–j) 2D XRD patterns along the [100] zone axis under different defect percentages. (k–o) 2D XRD patterns along the [111] zone axis under different defect percentages.

Supplementary Table H The predictions of Phonopy and Model-4 under different defect percentages in the sample structure (ICSD_033627).

Depletion	0%	25%	50%	75%	100%
Chemical component	Ag42 Se2	Ag32 Se2	Ag21 Se2	Ag11 Se2	Se2
Phonopy classification	229	1	1	1	229
Model-4's predict - Zone 100	229	41	216	5	229
Model-4's predict - Zone 111	229	1	1	1	229

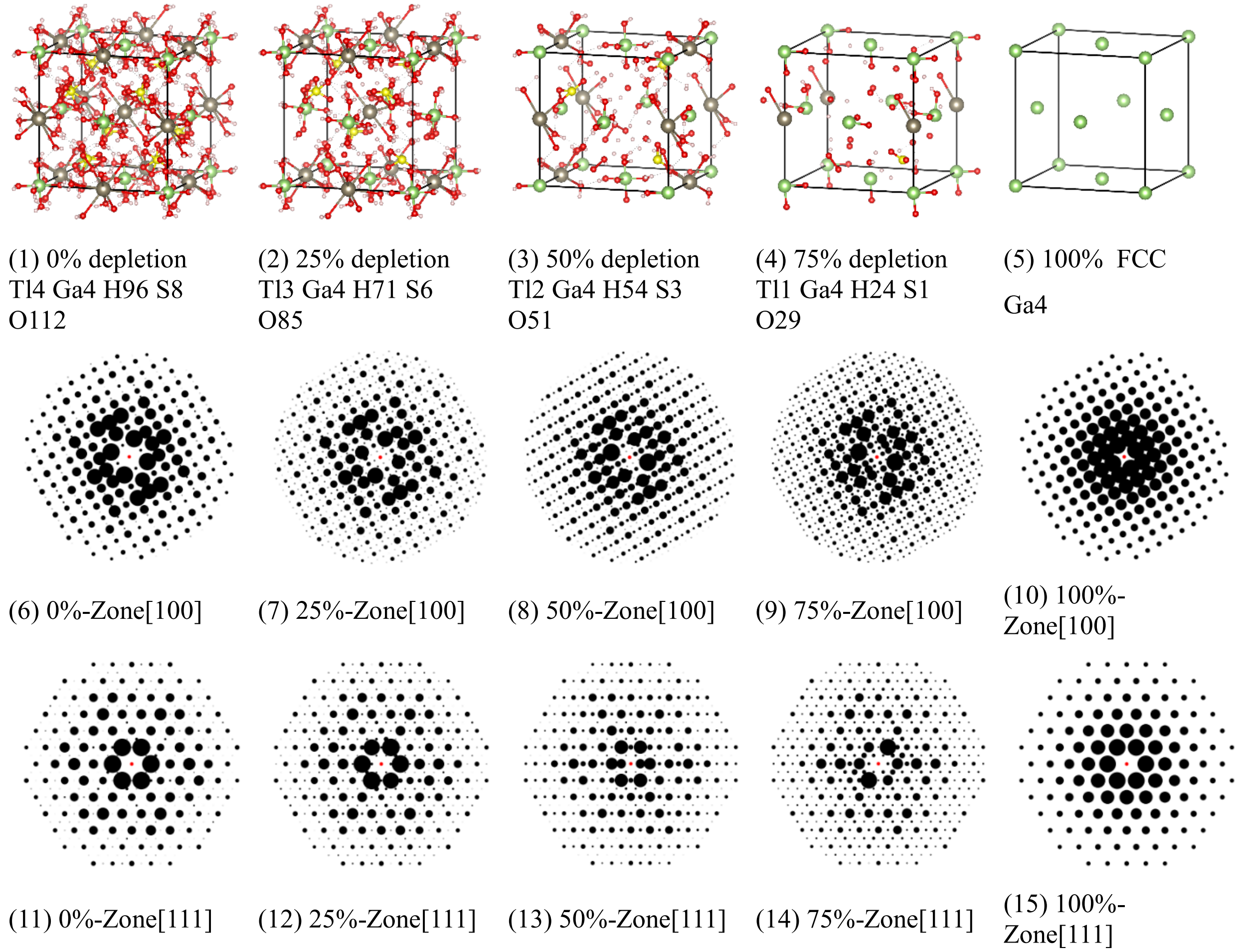

Supplementary Figure R (a–e) The unit cell of the structure (ICSD_280554) after applying defects. (f–j) 2D XRD patterns along the [100] zone axis under different defect percentages. (k–o) 2D XRD patterns along the [111] zone axis under different defect percentages.

Supplementary Table I The predictions of Phonopy and Model-4 under different defect percentages in the sample structure (ICSD_280554).

Depletion	0%	25%	50%	75%	100%
Chemical component	Tl4 Ga4 H96 S8 O112	Tl3 Ga4 H71 S6 O85	Tl2 Ga4 H54 S3 O51	Tl1 Ga4 H24 S1 O29	Ga4
Phonopy classification	205	1	1	1	225
Model-4's predict - Zone 100	205	29	2	29	226
Model-4's predict - Zone 111	205	198	87	97	225

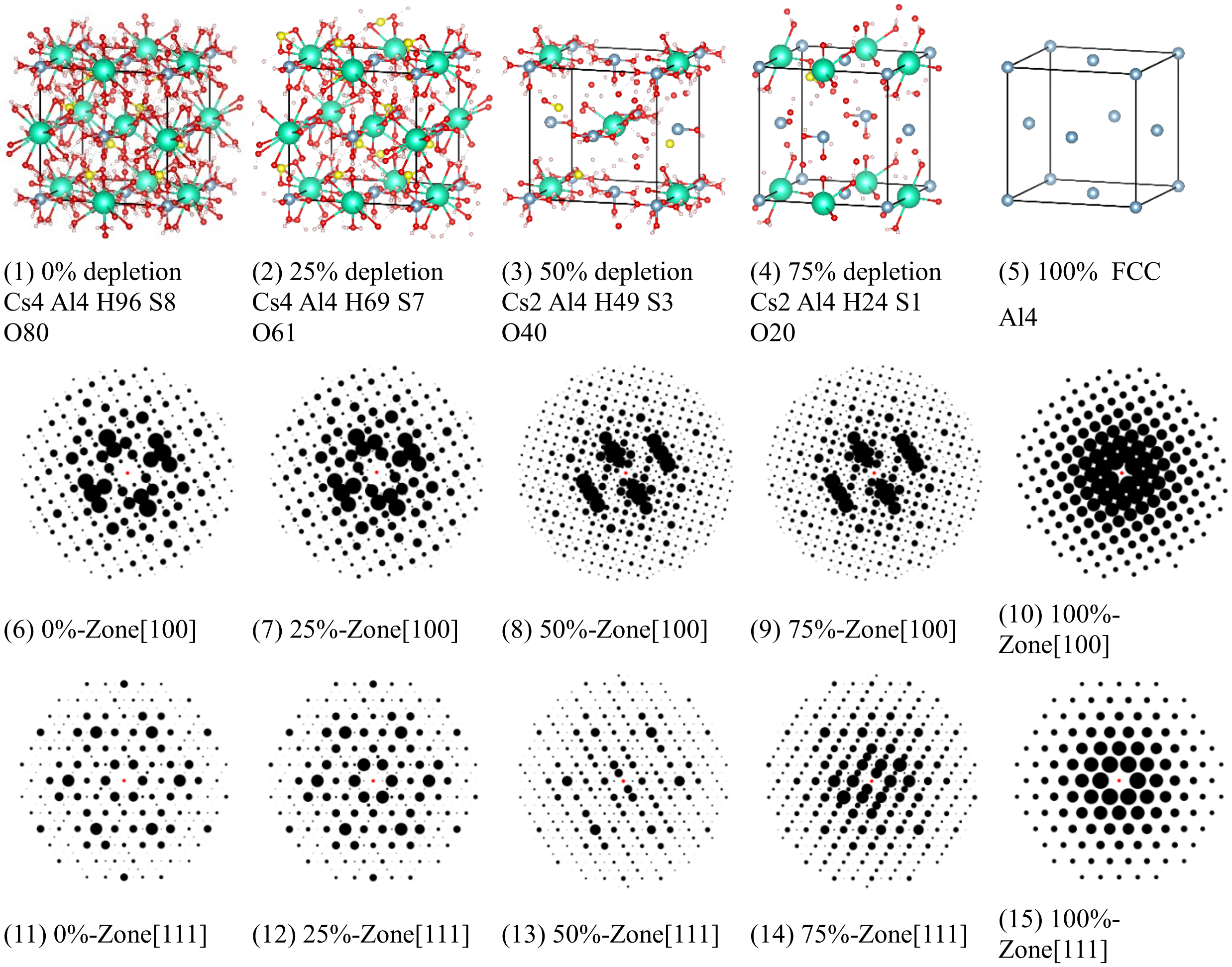

Supplementary Figure S (a–e) The unit cell of the structure (ICSD_ 201215) after applying defects. (f–j) 2D XRD patterns along the [100] zone axis under different defect percentages. (k–o) 2D XRD patterns along the [111] zone axis under different defect percentages.

Supplementary Table J The predictions of Phonopy and Model-4 under different defect percentages in the sample structure (ICSD_201215).

Depletion	0%	25%	50%	75%	100%
Chemical component	Cs4 Al4 H96 S8 O80	Cs4 Al4 H69 S7 O61	Cs2 Al4 H49 S3 O40	Cs2 Al4 H24 S1 O20	Al4
Phonopy classification	205	1	1	1	225
Model-4's predict - Zone 100	205	198	2	2	226
Model-4's predict - Zone 111	205	228	87	87	196

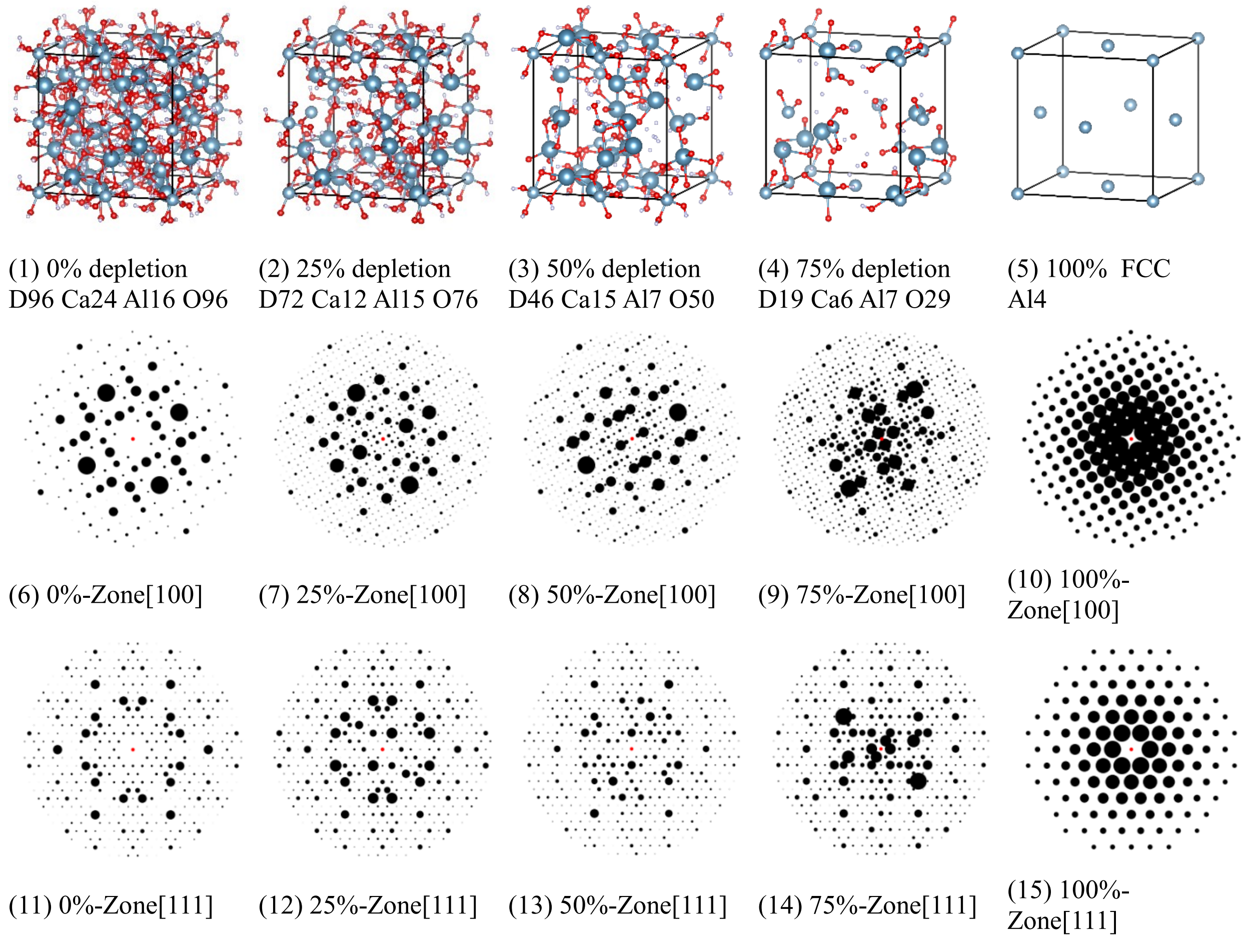

Supplementary Figure T (a–e) The unit cell of the structure (ICSD_ 171830) after applying defects. (f–j) 2D XRD patterns along the [100] zone axis under different defect percentages. (k–o) 2D XRD patterns along the [111] zone axis under different defect percentages.

Supplementary Table K The predictions of Phonopy and Model-4 under different defect percentages in the sample structure (ICSD_171830).

Depletion	0%	25%	50%	75%	100%
Chemical component	D96 Ca24 Al16 O96	D72 Ca12 Al15 O76	D46 Ca15 Al7 O50	D19 Ca6 Al7 O29	Al4
Phonopy classification	230	1	1	1	225
Model-4's predict - Zone 100	230	119	70	29	226
Model-4's predict - Zone 111	230	198	82	82	196

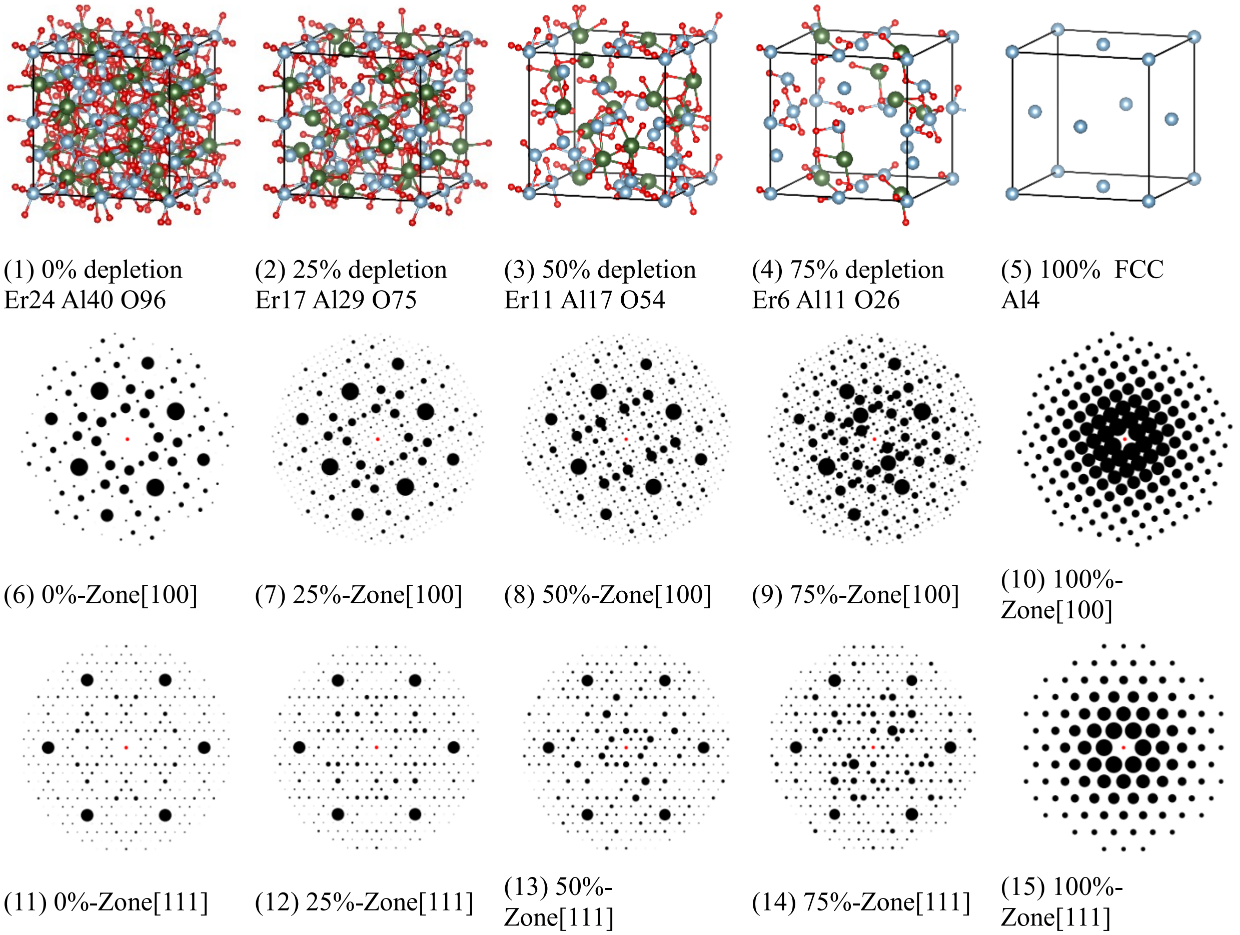

Supplementary Figure U (a–e) The unit cell of the structure (ICSD_170146) after applying defects. (f–j) 2D XRD patterns along the [100] zone axis under different defect percentages. (k–o) 2D XRD patterns along the [111] zone axis under different defect percentages.

Supplementary Table L The predictions of Phonopy and Model-4 under different defect percentages in the sample structure (ICSD_170146).

Depletion	0%	25%	50%	75%	100%
Chemical component	Er24 Al40 O96	Er17 Al29 O75	Er11 Al17 O54	Er6 Al11 O26	Al4
Phonopy classification	230	1	1	1	225
Model-4's predict - Zone 100	230	204	119	8	226
Model-4's predict - Zone 111	230	218	119	82	196

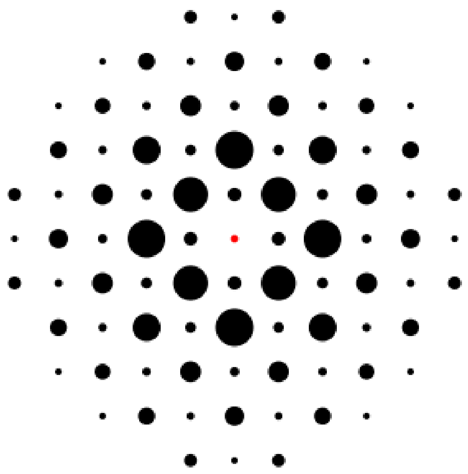

(1) icsd_168253
zone axis [100]
space group 99

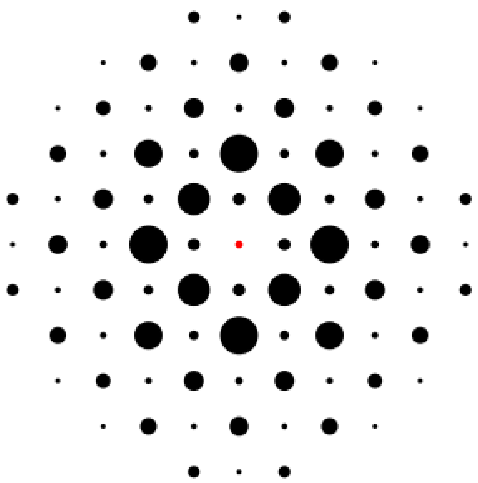

(2) icsd_028591
zone axis [100]
space group 123

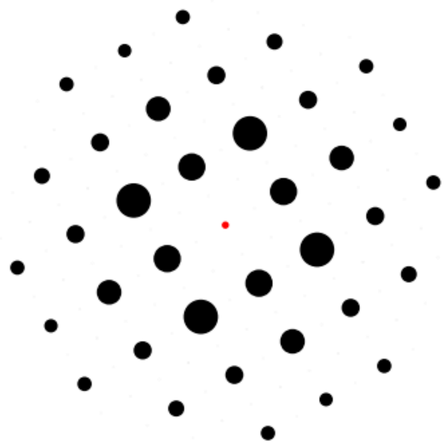

(3) icsd_084806
zone axis [100]
space group 216

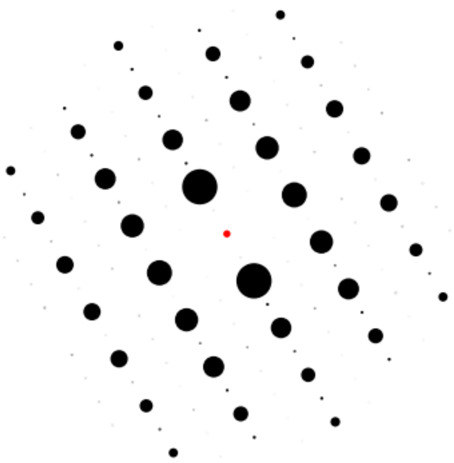

(4) icsd_174304
zone axis [100]
space group 14

Supplementary Figure V (1–4) illustrates that different CIFs from multiple space groups can produce highly similar 2D XRD patterns. This high degree of visual similarity can lead to model confusion and reduces overall prediction accuracy
